# Genetic legacy of state centralization in the Kuba Kingdom of the Democratic Republic of the Congo

**DOI:** 10.1073/pnas.1811211115

**Published:** 2018-12-24

**Authors:** Lucy van Dorp, Sara Lowes, Jonathan L. Weigel, Naser Ansari-Pour, Saioa López, Javier Mendoza-Revilla, James A. Robinson, Joseph Henrich, Mark G. Thomas, Nathan Nunn, Garrett Hellenthal

**Affiliations:** ^a^University College London Genetics Institute, University College London, London WC1E 6BT, United Kingdom;; ^b^Centre for Mathematics and Physics in the Life Sciences and Experimental Biology, University College London, London WC1E 6BT, United Kingdom;; ^c^Department of Economics, Bocconi University, Milan 20100, Italy;; ^d^Department of International Development, London School of Economics, London WC2A 2AE, United Kingdom;; ^e^Faculty of New Sciences and Technologies, University of Tehran, Tehran, Iran;; ^f^Cancer Institute, University College London, WC1E 6DD London, United Kingdom;; ^g^Laboratorios de Investigación y Desarrollo, Facultad de Ciencias y Filosofía, Universidad, Peruana Cayetano Heredia, Lima, Peru;; ^h^Unit of Human Evolutionary Genetics, Institut Pasteur, 75015 Paris, France;; ^i^Harris School of Public Policy, University of Chicago, Chicago, IL 60637;; ^j^Department of Human Evolutionary Biology, Harvard University, Cambridge, MA 02138;; ^k^Department of Genetics, Evolution and Environment, University College London, London WC1E 6BT, United Kingdom;; ^l^Department of Economics, Harvard University, Cambridge, MA 02138

**Keywords:** population genetics, demographic inference, anthropology, history

## Abstract

Few phenomena have had as profound or long-lasting consequences in human history as the emergence of large-scale centralized states in the place of smaller scale and more local societies. This study examines a fundamental, and yet unexplored, consequence of state formation: its genetic legacy. We studied the genetic impact of state centralization during the formation of the eminent precolonial Kuba Kingdom of the Democratic Republic of the Congo (DRC) in the 17th century. We analyzed genome-wide data from over 690 individuals sampled from 27 different ethnic groups from the Kasai Central Province of the DRC. By comparing genetic patterns in the present-day Kuba, whose ancestors were part of the Kuba Kingdom, with those in neighboring non-Kuba groups, we show that the Kuba today are more genetically diverse and more similar to other groups in the region than expected, consistent with the historical unification of distinct subgroups during state centralization. We also found evidence of genetic mixing dating to the time of the Kingdom at its most prominent. Using this unique dataset, we characterize the genetic history of the Kasai Central Province and describe the historic late wave of migrations into the region that contributed to a Bantu-like ancestry component found across large parts of Africa today. Taken together, we show the power of genetics to evidence events of sociopolitical importance and highlight how DNA can be used to better understand the behaviors of both people and institutions in the past.

## Significance

State centralization occurs when previously separate communities are united, forming a single political system often associated with economy, trade, warfare, and culture. One example is the precolonial Kuba Kingdom of the Democratic Republic of the Congo (DRC). Using genetic data from over 690 individuals from the DRC, we compared individuals whose ancestors were part of the Kingdom to individuals from other neighboring groups. We found a genetic legacy of state formation that can be explained by the joining and subsequent mixing of groups at the time of state centralization, as well as evidence of gene flow facilitated by the Kingdom’s infrastructure. We characterize the genetic history of this region and show the power of DNA to reveal information on societal systems where few written records exist.

Prominent theories of comparative economic development note the importance of state formation for specialization of production, for trade, for innovation, and for warfare (1–3). Scholars have documented enduring legacies of state centralization on economic activity (4–7), politics (8, 9), corruption (10), violence (11), civil society (12–14), linguistics (15), and culture (16–19). It is not clear whether states promote genetic diversity or constrain it. On the one hand, states may increase genetic diversity by facilitating movement among previously disparate groups of people; on the other hand, they may decrease genetic diversity by reducing exchange with external populations. In addition, increased political, economic, and social stratification could decrease the genetic diversity within, but increase diversity between, strata, and elite dominance could decrease overall genetic diversity (20–23). We examine the effect of one episode of state centralization—the early 17th century formation of the Kuba Kingdom in the central Democratic Republic of the Congo (DRC)—on subsequent patterns of genetic diversity and consider what genetic analyses can add to our understanding of historical events.

**Fig. 1.**
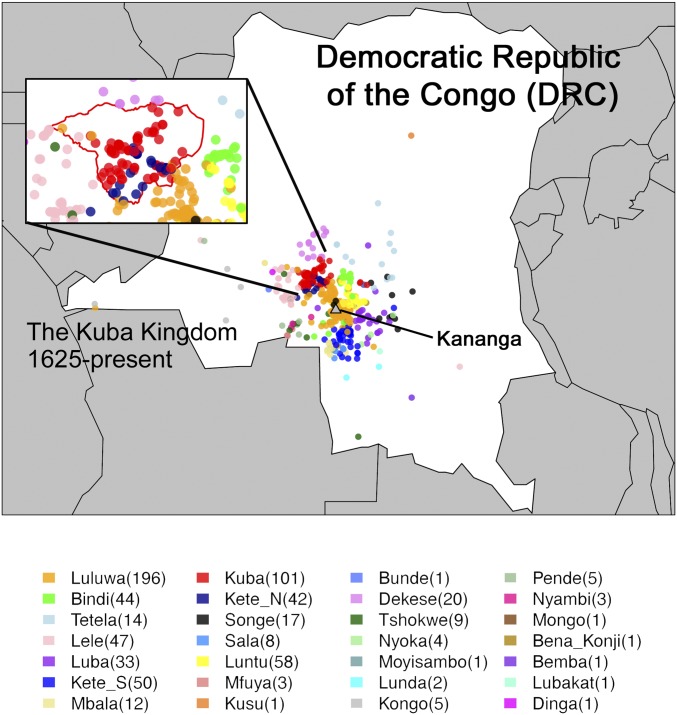
Sampled individuals from the DRC. Each of 693 sampled individuals is colored according to self-identified ethnicity and placed on the map according to village of residence. The boundaries of the Kuba Kingdom at its largest are depicted in red in the expanded box, with Kete split into northern (“Kete_N”) and southern (“Kete_S”) groups based on genetic clustering. The legend at *Bottom* gives the number of individuals per group in parentheses.

According to archaeological evidence and oral histories, the Kasai Central Province of the DRC was settled through migration of multiple waves of Bantu-speaking peoples before 500 BCE (24–26), followed by a mass migration of Mongo speakers that populated the region during the 13th to 15th centuries (27, 28). These were initially organized in small-scale societies, with no political hierarchy beyond the village level. This was the case until the early 17th century, when, according to oral history, a foreign trader named Shyaam unified the villages into a politically centralized state that became known as the Kuba Kingdom (Fig. 1). The Kingdom expanded rapidly, uniting all villages that fell within its natural borders, which were defined by the river network of the Kasai, the Sankuru, and the Lulua Rivers (2, 29–31). The Kingdom comprised both groups who had recently migrated to the region and populations that had previously resided in the area. Villages on the other sides of these river boundaries were never incorporated into the Kingdom and remained small-scale societies with no political authority above the village chief (29, 32, 33). The descendants of the group who had recently migrated to the region but lived to the west of the Kasai River became known as the Lele. The common origins of the Kuba and Lele are consistent with their speaking different dialects of the same language today (30, 32, 33).

In stark contrast to the nearby villages, the Kuba Kingdom had many institutions associated with modern centralized states, such as a national capital, division of political authority, an oral constitution, a tiered legal system, a professional police force, a standing army, universal taxation, and public goods provision (*SI Appendix*, section S1). As a result, the economic, political, and social organization of the Kuba Kingdom has been of great interest to historians, anthropologists, political scientists, and economists (2, 16, 29, 30, 33–35). Scholars have compared the Kuba Kingdom to the world’s most sophisticated historical states, including Augustan Rome, Imperial Japan, and Ancient Egypt (34, 35). The Kingdom continues to exist today, though with diminished stature following the arrival of Belgian colonists in the early 20th century. Although it has witnessed a weakening of many of its traditional institutional structures (much like other ethnic groups in the DRC), the Kingdom continues to exist today, having survived Congo’s history of colonial rule and postcolonial economic stagnation (30, 36).

Here, we use genomic data to investigate the genetic legacy of the Kuba Kingdom and the history of the central DRC as a whole. We collected genome-wide genotype data from individuals living in Kananga, the capital of Kasai Central Province (Fig. 1 and *SI Appendix*, section S2). We analyzed 250,000 to 600,000 autosomal SNPs in 693 individuals representing 27 different self-declared groups. In 542 of these individuals, we also genotyped 1,149 Y chromosome and 405 mitochondrial (mtDNA) variable sites. The dataset includes 101 self-identified members of the Kuba, the best available indicator that an individual’s ancestors lived in the Kuba Kingdom. These 101 individuals include 1 to 47 individuals from each of the 16 Kuba subgroups that were historically part of the state.

To understand the genetic impact of the Kuba Kingdom, we compared descendants of people that were part of the Kuba Kingdom (Kuba) to descendants of the other neighboring stateless groups (non-Kuba). We addressed four major questions: Is the level of genetic diversity among Kuba significantly higher, lower, or indistinguishable from neighboring non-Kuba? Is genetic differentiation among non-Kuba groups greater or less than that between Kuba and non-Kuba? Can the patterns observed be explained by the historical formation of the Kuba Kingdom, and, if so, how can they inform us about the effects of state centralization? More generally, what is the ancestral history of the present-day peoples of Kasai Central Province?

## Results

To examine the genetic diversity of Kuba relative to non-Kuba, we compared individuals from the six largest groups sampled in our dataset, each of which had more than 40 members: Bindi, Kete, Kuba, Lele, Luluwa, and Luntu. We focused on groups with more than 40 individuals because results were inconsistent when reducing to fewer individuals (discussed in *SI Appendix*, section S7). We assigned haplogroups and measured diversity in each of the mtDNA, which is inherited from mother to offspring, and the nonrecombining portion of the Y chromosome, which is inherited from father to son. Diversity levels in mtDNA haplogroups were similar across all six groups while only the Lele differed from the others in Y-chromosome diversity (*SI Appendix*, section S3). In particular, the Lele showed twofold lower genetic diversity on the Y chromosome, consistent with a relatively lower number of breeding males and perhaps reflecting their known practice of polygamy (32, 33, 37).

**Fig. 2.**
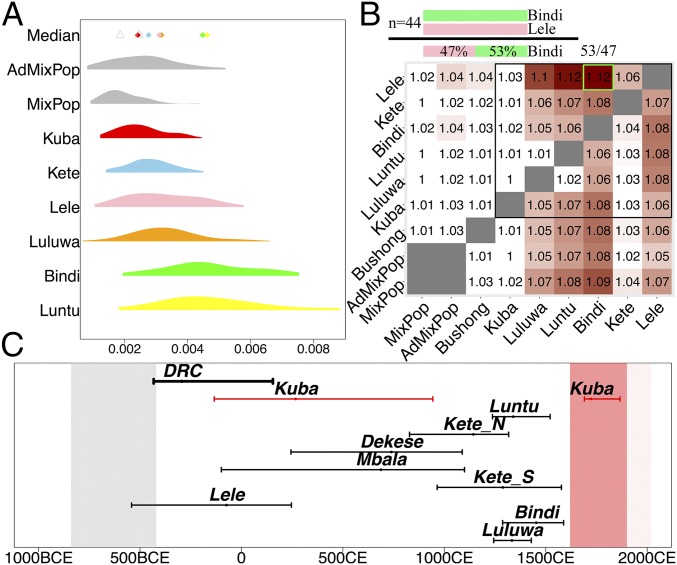
Kuba are less genetically isolated relative to other DRC groups. (*A*) Average lengths (cM) of tracts shared identical-by-descent (IBD) among pairs of individuals within each group and two simulated populations, MixPop and AdMixPop. Points along the top row provide the median value per group colored as in Fig. 1. The differences between the Kuba and Kete are significantly different following permutation-based resampling. (*B*) Average factor increase in the proportion by which members of a given ethnicity (column) share most recent ancestors with other individuals from their own group versus individuals from a different group (row), as illustrated in the *Top* schematic and green box. All groups are subsampled to contain *n* = 44 individuals to adjust for sample size effects. (*C*) Inferred dates (and 95% CIs) of admixture events in each DRC ethnicity when using all sampled groups as surrogate admixing sources, with vertical bands depicting the time periods of the Kuba Kingdom before Belgian colonization (red; ∼1620 to 1900) and the earliest local iron-working sites (gray; ∼840 BCE to 420 BCE). The inferred date when analyzing all DRC individuals jointly, using only non-DRC surrogate admixing sources, is shown in bold black.

For the remainder of this study, we focused on autosomal DNA, which is inherited equally from both sexes and contains many thousands of times more information than mtDNA and Y-chromosome data. We inferred the genetic diversity of Kuba and neighboring groups using two techniques that measure the lengths of autosomal DNA segments that share a recent common ancestor among individuals from the same group, with longer matching segments implying more recent shared ancestry (38, 39) (*SI Appendix*, section S4). The Kuba were consistently inferred to have the highest relative average amount of genetic diversity (Fig. 2*A*). This finding is statistically significant, with a permutation-based *P* value of <0.005 across each comparison of Kuba to the five non-Kuba groups tested (*SI Appendix*, Table S7). This cannot be explained by sampling biases since the Kuba are a minority group in the city of Kananga (16), are not our largest sampled ethnicity, and the origins of Kuba participants span a smaller geographic area than most other study groups (Fig. 1 and *SI Appendix*, Table S2).

To explore genetic similarity among DRC individuals, we measured the autosomal genetic distance between each pair of individuals using total variation distance (TVD) (31), exploiting correlations among neighboring SNPs to increase power (*SI Appendix*, section S5 and Fig. S4) (39, 40). Reflecting the fact that the recorded origins of the 693 individuals span a small geographic area (Fig. 1), we observed relatively high levels of autosomal genetic similarity among them (F_ST_ < 0.0018) (*SI Appendix*, Table S8). Despite this, on average, individuals from different groups were more genetically different from individuals from the same group (permutation-based *P* values of <0.005 for all pairwise comparisons) (*SI Appendix*, Tables S9 and S10). Notably, on average, the genetic distance between individuals from different groups tended to be smaller when one of those individuals identified as Kuba (*SI Appendix*, Figs. S5 and S6). For example, the program fineSTRUCTURE (39), which clusters people based solely on patterns of genetic similarity, clustered Kuba with individuals of a different label more often than it clustered non-Kuba with individuals of a different label (*SI Appendix*, section S6).

To formally address the question of whether Kuba are more genetically similar to non-Kuba than non-Kuba are to other groups, we quantified the relative degree of genetic isolation between pairings of groups. To do so, for each individual in each pairwise comparison of the six ethnic groups for which we have more than 40 samples, we inferred the proportion of autosomal DNA for which the individual shares a most recent ancestor with an individual from their own label versus an individual from the other label (Fig. 2*B*, *Top*). We expected this proportion to be >1 for any particular comparison, reflecting more recent shared ancestry within than between groups. Indeed this was the case (Fig. 2*B*). However, the Kuba had notably smaller proportions across all comparisons. This observation was not symmetric—each of the five non-Kuba groups had a notably higher ratio of recent ancestry matching to members of their own label versus their matching to Kuba. This consistent asymmetry, which only occurred in comparisons with Kuba, demonstrates that the Kuba are relatively more genetically similar to people from neighboring ethnicities in the region. This pattern was also seen for Bushong, which represent the only subgroup of Kuba with a sample size >40 (Fig. 2*A* and *SI Appendix*, section S7).

We now explore potential explanations for the Kuba’s increased genetic diversity and increased genetic similarity to non-Kuba, compared with the analogous measures in non-Kuba groups. Possible drivers of these genetic patterns include the following: (*i*) The Kuba descend from genetically differentiated groups that were unified during the formation of the Kuba Kingdom. (*ii*) Kuba individuals are descended from a population that had higher genetic diversity before the formation of the Kuba Kingdom: e.g., due to a higher effective population size rather than the institutions of the Kingdom. (*iii*) The social structures and transport networks established by the Kuba Kingdom encouraged gene flow from outside sources during this period.

There is no a priori evidence to suggest that explanation *ii* is true. To test whether explanation *i* is sufficient to explain our observations without requiring *ii*, we mimicked *i* by making two artificial populations of 44 people. The first (“MixPop”) consists of the genetic variation data from randomly selected individuals from six ethnicities: Dekese, Songe, Tetela, Mbala, Tshokwe, and Sala, merged into a single population. The second (“AdMixPop”) assumes people from these groups not only merged but also intermixed 10 generations ago (∼220 to 330 y ago, around the time of the formation of the Kuba Kingdom), mimicking a scenario where the Kuba Kingdom facilitated gene flow between the groups it united (*SI Appendix*, section S8). We repeated our analyses, but now including each of these artificial populations, and found that patterns in each showed good concordance with those observed in the Kuba today (Fig. 2 *A* and *B*), while pointing toward some degree of intermixing among the unified groups (*SI Appendix*, Fig. S11). These comparisons suggest that the consolidation of peoples during the formation of the Kuba Kingdom, with similar levels of intergroup diversity as observed in the DRC today, is sufficient to explain contemporary genetic patterns in Kuba, and explanation *ii* is not necessary. This is in agreement with ethnographic reports documenting high geographic mobility and marriage between the ethnic units that originally comprised the Kuba Kingdom (29, 30).

To assess evidence for explanation *iii*, we applied GLOBETROTTER (41) to test for signals of the intermixing of groups (i.e., admixture) in our DRC sample, using a dataset also including people from across Africa as potential surrogates to the mixing groups (*SI Appendix*, section S9). We dated a unique admixture event in the Kuba to ∼1720 CE (95% CI: 1667 to 1891 CE), more recent than any other inferred event, and involving the mixing of sources that are genetically similar to other sampled DRC ethnicities. This date closely brackets the period of the formation of the Kuba Kingdom to after the early 17th century and before Belgian colonization (Fig. 2*C*). This provides evidence for a migration of neighboring groups into the Kuba Kingdom, with a lack of clear evidence for migration elsewhere in the region during this time period (at least that led to detectable levels of intermixing). Overall, these findings suggest that the political framework of the Kingdom supported outside migration in and that this peaked between around 1660 to 1895 CE.

**Fig. 3.**
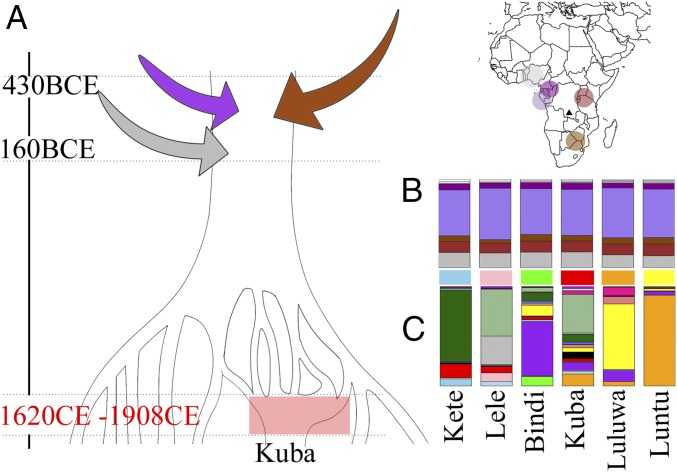
Proposed demographic history for the Kuba. (*A*) Simplified demographic history of sampled groups consistent with genetic patterns. All individuals share an admixture event, dated to 430 BCE to 160 CE, involving sources best represented by the present day Yoruba of Nigeria (gray), Nzebi of Gabon (purple), and Bantu speakers of East (LWK) and Southern (SEBantu) Africa (maroon/brown). Subsequently, groups were isolated from each other, after which the establishment of the Kuba Kingdom, bordered in red, consolidated some groups. (*B*) SOURCEFIND inferred ancestry proportions matching to the non-DRC sources highlighted in the *Top Right* map, across six DRC groups with >40 individuals, suggested shared ancestral histories. Contributions <3% are colored white. (*C*) SOURCEFIND inferred ancestry proportions matching to both DRC and non-DRC sources, colored as in Fig. 1, for the same groups, reflecting more recent genetic differentiation among them.

To gain insights into the history of the Kasai Central Province before the formation of the Kuba Kingdom, we used a Bayesian mixture model (42) to infer the average proportion of DNA for which sampled individuals from each DRC group share most recent ancestry with non-DRC peoples (*SI Appendix*, sections S10 and S11). Because people living nearby typically share more recent ancestry, we would expect the most recent common ancestors shared between a DRC person and a non-DRC person to have lived farther back in time relative to those shared among two people who are both from the DRC. Consistent with this, on average, DRC individuals matched shorter genetic segments to non-DRC people [∼0.65 centimorgans (cM)] relative to other DRC people (∼1.76 cM) (*SI Appendix*, section S11). We can also assess whether DRC groups likely experienced distinct admixture events from external populations, because such a scenario would likely lead to different matching patterns among the groups under this analysis (43). Instead, we found proportions of matching to non-DRC people to be very similar across all DRC groups, with relatively large proportions matching to individuals from Gabon, Nigeria, and Kenya (Fig. 3*B*). This contrasted with an alternative analysis aimed at inferring the proportion of DNA for which each DRC group’s individuals share most recent ancestors with all samples, including the DRC (Fig. 3*C*), which captures the sharing of ancestors at more recent timescales. In this alternative analysis, there were large differences in average matching across DRC ethnic groups, highlighting how some are more closely related to each other. Overall, these patterns were consistent with the subtle genetic differences among contemporary DRC groups being due to relatively recent isolation rather than varying degrees and/or sources of introgression from non-DRC sources. In particular, detectable genetic differences between DRC groups demonstrate that they have become isolated from one another at some point in the past (Figs. 2 *A* and *B* and 3*C* and *SI Appendix*, section S11). On the other hand, the similarity in which the DRC peoples, regardless of ethnicity, relate genetically to non-DRC peoples (Fig. 3*B*) suggests that this isolation has occurred only in the recent past.

We sought to place an upper bound on the date of isolation among DRC groups. If the mixture proportions in Fig. 3*B* are attributable, in part, to admixture between sources represented by our non-DRC surrogates, a parsimonious explanation for the similarity in proportions among groups is that their ancestors were not isolated from one another when this admixture occurred. We note an alternative, but less parsimonious, possibility is that the same outside sources intermixed separately with the ancestors of each modern DRC group and that each source contributed roughly the same proportion of DNA during each such independent admixture event. Assuming the former scenario, we combined all of our DRC samples into a single population and tested for admixture in this population, using non-DRC groups as surrogates. We inferred a single date of admixture occurring over 2,000 y ago (*SI Appendix*, Table S21). This inferred event (290 BCE; 95% CI: 430 to 160 BCE) predates the formation of the Kuba Kingdom and predominantly involves sources related to Bantu-speaking peoples from the northwest, south, and central Africa (*SI Appendix*, Figs. S16 and S17), with these three distinct sources intermixing at around the same time. Therefore, a likely demographic scenario explaining DRC group genetic patterns (Fig. 3*A*) involves their becoming isolated from each other less than ∼2,300 y ago, consistent with the relatively high genetic similarity observed among them today (*SI Appendix*, sections S5 and S11). Our inferred date and sources for this >2,000-y-old admixture event are consistent with a late wave of migrations into the region that corresponds well with evidence of iron working in the Congo Basin around 300 BCE (24, 44, 45). In additional analyses testing for admixture in 36 neighboring African groups using all others (including the DRC) as surrogates for the admixing sources, we found evidence of >5% introgression from a source partially matching to the DRC in 14 cases (*SI Appendix*, section S13). When excluding the DRC, these proportions are replaced by other Bantu-speaking populations, including southeastern Bantu speakers (SEBantu) and Nzebi of Gabon (Nzebi_Gab), suggesting that our DRC samples act as a good representative for ancestry relating to the migrations of Bantu-speaking peoples (46, 47).

## Discussion

Our genetic analyses provide concrete evidence supporting existing accounts of the social consequences of the Kuba Kingdom (16), which, until now, have been based almost solely on oral evidence since written sources are unavailable before European contact (29). Genetic evidence supports the view that the formation of the Kuba Kingdom resulted in greater mobility across space within the Kingdom. In part, this was because people regularly moved to the capital city, which was the hub of market and politics. But it was also because the Kingdom facilitated greater specialization of production and with it greater trade (29, 33). It also enabled greater upward social and economic mobility, which led to more spatial mobility, as well as mixing across social and ethnic groups (29, 31, 33). Our inferred date of admixture in Kuba to 1660 to 1895 CE is remarkably consistent with the time line derived from oral histories that was ingeniously pieced together by Jan Vansina, who dated the beginning of the rule of the first King of the Kingdom, King Shyaam, to 1620. This was dated using the list of previous kings, combined with mentions of a solar eclipse (that occurred in 1680) and the observation of Halley’s comet in 1835 (29).

Our analyses show that the present-day Kuba and Lele are genetically differentiable, suggesting a period of isolation, despite both descending from the same 13th to 15th century migrant wave. Because the Lele were excluded from the subsequent Kingdom, researchers have compared present-day Lele and Kuba members to study the long-term effects of state formation, as reported in the anthropology (32, 33), history (29), and economics (16) literatures. For example, Lowes et al. (16) compared long-run differences in the psychology of Kuba and Lele. However, a key assumption of their analysis is that there was not significant movement and mixing between the Kuba and Lele after the formation of the Kuba Kingdom. We provide empirical support for this assumption.

By comparing descendants of the Kuba Kingdom to descendants of neighboring groups, we illustrate how the unification and consolidation of a centralized state shaped the genetic diversity of present-day peoples. Importantly, our findings showcase the potential of DNA to reconstruct past population dynamics without prior knowledge. This is of particular relevance, for example in this case, when historical reconstructions have been based largely on oral narratives which may be subject to biases (48). Here, we used only DNA and modern ethnic labels in the DRC to infer that ancestors of these groups likely separated from one another within the past 2,300 y, after which the ancestors of one ethnicity (Kuba) had relatively more genetic interactions with surrounding people and hence increased their genetic diversity. Thus, our study demonstrates the potential of genomic data from present-day peoples to unearth polities in areas where historical records are nonexistent or limited.

## Materials and Methods

### Datasets.

DNA was extracted from saliva samples from three separate collections in the city of Kananga (Fig. 1). Informed verbal or written consent was obtained for the analysis of genetic data obtained from participants. All experiments involving human subjects were approved by the Harvard Internal Review Board (IRB00000109; Protocol 24087). The ethnic group of individuals was self-reported and cross-validated as detailed in *SI Appendix*, section S2. DNA from the first 2013 collection was genotyped by ftDNA (https://www.familytreedna.com/), and DNA from the two subsequent collections (2014/2015) was genotyped by the personal genetics company 23andMe, Inc. (https://www.23andme.com/en-int/). For the cohort genotyped by 23andMe, data were also generated for the mtDNA and NRY uniparental systems. For the autosomal data, we performed dataset merges that differed in the number of individuals, the number of SNPs, and the inclusion of worldwide populations, though with substantial overlap. We refer to these as DRC-only, DRC-world, and DRC-all-world, and each dataset is described in full in *SI Appendix*, sections S2 and S9. For all analyses focused on exploring genetic diversity within the DRC, we used the DRC-only dataset. For admixture analyses and those focused on how DRC individuals relate genetically to global populations, we used the DRC-all-world dataset, with some analyses replicated with the DRC-world dataset as indicated. For the uniparental markers, 405 SNPs were genotyped on the mtDNA and 1,149 SNPs on the NRY for 542 individuals genotyped by 23andMe. Of these, we analyzed 540 individuals that had accompanying data for group identity and gender. Raw genotype data cannot be made available due to restrictions imposed by the ethics approval.

### Uniparental Marker Analyses.

Haplogroups in the nonrecombining region of the Y chromosome (NRY) were assigned to all male individuals using a maximum likelihood approach implemented in Yfitter (49). mtDNA haplogroups were assigned to all individuals using HaploGrep v2 (50). NRY and mtDNA haplogroup diversities were estimated using Arlequin v3.1 (51) (*SI Appendix*, section S3).

### Chromosome Painting.

Autosomal datasets were first phased using SHAPEITv2 (52) with default parameters and using build 37 genetic maps. Then, to explore patterns of shared ancestry to DRC samples and to non-DRC worldwide groups, we performed two analyses implemented in CHROMOPAINTER (39): (*i*) All-donors—recipient individuals are matched to all individuals from the DRC and all other worldwide groups (excluding themselves). (*ii*) Non-DRC-donors—recipient individuals are matched to individuals from all worldwide groups except all DRC samples (and themselves).

CHROMOPAINTER infers a haplotype “sharing profile” for each individual that consists of the inferred proportion of contiguous DNA segments (i.e., haplotypes) that the individual matches to members of each donor group, with groups defined using population labels. The raw CHROMOPAINTER coancestry matrices are provided in Dataset S1. As well as using haplotype information, we additionally ran CHROMOPAINTER using the “unlinked” model, which considers all SNPs as independent, to explore how much information is gained through using haplotype information in this dataset.

### Inferring Within-Group Genetic Diversity.

We employed two approaches to explore within-group genetic diversity (*SI Appendix*, section S4). First, we applied fastIBD (38) to groups sampled in the DRC-only dataset. For each chromosome of each group with >30 individuals, fastIBD was run for 10 independent runs, with an identity-by-descent (IBD) threshold of 10^−10^ for every pairwise comparison of individuals within each ethnicity. For each pairwise comparison of groups, we used 1,000 permutations of labels to assess whether the mean within-group fastIBD values differed significantly between the two groups. Second, we inferred the total expected number of haplotype segments that each Kuba individual shares with other Kuba using CHROMOPAINTER, comparing this to the amount individuals from each other ethnic group with >30 individuals share with other individuals of the same label (23). When comparing the Kuba to each other group, we matched for sample size since this can affect the inferred segment lengths. In particular, when comparing to group B, we painted each Kuba individual across their genome using *X* = *min*(*n_A_* − 1,*n_B_* − 1) other Kuba and each group B individual using *X* other individuals from group B, where *n*_*A*_ is the number of sampled Kuba and *n*_*B*_ is the number of sampled individuals from group B (B = Luluwa, Bindi, Luntu, Kete N, Kete S, Kete, Lele, and Luba). Then, for each comparison between Kuba and group B, we used a two-sample *t* test to assess whether the mean segment count under this painting protocol was higher in the Kuba relative to group B. These *t* test values are indicative only as significance is challenging to assess here given that the inferred paintings for individuals include overlapping donors and hence are not independent, and permutations are impractical given the computational expense of this analysis. To test for consistency across Kuba subgroups, these analyses were repeated, replacing the Kuba with the three Kuba subgroups with largest sample size: Bushong (*n* = 47), Ngeende (*n* = 19), and Pyang (*n* = 11). We also explored sample size effects, by using random samples of 11 and 19 Bushong (matching the sample size of the Pyang) and subsampling of all groups to 33 individuals (matching the sample size of the Luba).

### Genetic Distance Between Individuals/Groups.

For the autosomes, F_ST_ between groups was calculated using PLINK v1.9 (53), and differences between groups’ sharing profiles were assessed using TVD (40). When reporting the TVD between two groups, we first averaged sharing profiles across individuals within each of the two groups, and then reported TVD between these averages. For each pair of groups, under each of the All-donors and Non-DRC-donors analyses, we tested whether the groups’ sharing profiles were significantly different by randomly permuting labels among individuals from the two groups and calculating the groupwise-TVD between these redefined (permuted) “groups.” For empirical *P* values, we report the proportion out of 1,000 permutations for which this groupwise-TVD was greater than the observed groupwise-TVD for the nonpermuted data (*SI Appendix*, section S5).

### Haplotype-Based Clustering.

Haplotype-based clustering was implemented in fineSTRUCTURE (39), as described in *SI Appendix*, section S6. We then assessed whether each individual should be reassigned based on which individuals they cluster with across all MCMC samples, using the procedure described in Leslie et al. (40).

### Inferring Ratios of Most Recent Ancestor Sharing Between Groups.

We measured the proportion of haplotype segments for which individuals share most recent ancestry with members of their own group relative to individuals from other groups. To do so, we pairwise compared all DRC groups that contained of >40 individuals, for which the Bindi (44 samples) was the smallest group. We restricted to >40 individuals because our results suggest that a smaller sample size may result in not fully capturing the full genetic diversity of each group (*SI Appendix*, section S7). When comparing individuals from group A to those in group B, we first randomly sampled *n* + 1 individuals from group A and *n* individuals from group B, where *n* = *min*(*n*_*A*_*−*1*,n*_*B*_) and *n*_*A*_ and *n*_*B*_ are the number of individuals in groups A and B, respectively. We then used CHROMOPAINTER with default settings to paint each of the *n* + 1 individuals from group A using the other 2*n* sampled individuals from groups A and B as donors, dividing the total proportion of genome-wide DNA each individual matches to group A by the amount they match to group B, as schematized in the *Top* of Fig. 2*B*. Fig. 2*B* shows the mean of this ratio across all *n* + 1 individuals in group A for each pairwise comparison, after reducing the sample size of all groups to 44 to match the sample size of the Bindi, to account for potential sample size effects. Under this sample-size matched analysis, for each group B, we used a two-sample *t* test to assess whether each group’s mean ratio of recent ancestry matching to individuals from their own ethnicity versus individuals from group B was significantly less than that of the other groups (*SI Appendix*, Table S14 and Dataset S2). However, these *t* test results are for comparison only as the inferred paintings for individuals include overlapping donors and hence are not independent.

### Simulations.

To explore whether patterns observed in the present-day Kuba are well explained by the union of genetically distinct groups, we created two artificial mixed populations and repeated our above analyses (*SI Appendix*, section S8). The first artificial population, MixPop, consisted of 44 individuals, to match the sample size of the Bindi, comprised of individuals randomly sampled from each of six groups: Dekese, Songe, Tetela, Mbala, Tshokwe, and Sala. We selected these groups as they exhibit differing levels of genetic diversity to one another, while also having the next largest sample sizes beyond the six ethnicities (Luluwa, Luntu, Lele, Kete, Bindi, and Luba) we compare with Kuba throughout. This artificial population is designed to mimic a scenario whereby several populations, with genetic diversity similar to that observed in the Kasai Central Province today, were unified at the time of state centralization. The second artificial population, AdMixPop, also consisted of 44 individuals using the Dekese, Songe, Tetela, Mbala, Tshokwe, and Sala, but now assuming that these six groups intermixed with each other from the time they merged. In particular, we assumed that an instantaneous admixture event occurred 10 generations ago, roughly coinciding with the start of the Kuba Kingdom, with respective contributions of 25%, 20%, 17%, 15%, and 6% from the six groups. This was followed by random mating among the admixed individuals until the present day. To do this, we used the simulation approach of Price et al. (54) where each haploid of the 44 simulated individuals is generated as a mosaic of haplotype blocks, with the size (in morgans) of each block sampled from an exponential distribution of rate 10, and with the genetic data from each such block matching that of a randomly sampled haploid among the 82 total sampled individuals from these six ethnicities. This produced an artificial admixed population comprising 88 such simulated haplotypes. We repeated our analyses of within-group genetic diversity and patterns of pairwise haplotype sharing using these artificial populations.

### Mixture Modeling of Ancestry Proportions.

Using the DRC-all-world dataset, we performed mixture modeling in SOURCEFIND (42) to infer the proportion of ancestry that each DRC target group with >30 individuals shares most recently with other sampled surrogate groups, including a “self-matching” term that reflects excess matching between members of the target group (*SI Appendix*, section S11). We inferred proportions of ancestry sharing between each DRC ethnicity and two different sets of surrogate groups as follows: (*i*) All other populations were used as surrogates—this analysis used the All-donors painting and included self-matching; (*ii*) only populations outside of the DRC were used as surrogates—this analysis used the non-DRC-donors painting.

For each target group and each of analyses *i* and *ii*, we ran SOURCEFIND for 200,000 iterations, discarding the first 50,000 iterations as burn-in and sampling posterior mixing coefficients every 5,000 iterations thereafter. We assumed a maximum of eight surrogates could have contribution >0 at each iteration, with an a priori expectation of four surrogates. Fig. 3 presents results for averaged sampled mixing coefficients across 30 posterior samples.

### Inferring and Dating Admixture Within and into the DRC.

We performed GLOBETROTTER (41) analyses designed to identify, describe, and date pulses of admixture within sampled groups. We first removed individuals that were classified by fineSTUCTURE into clusters of size 2 to 3. Individuals within each of these small clusters were of the same label, which could indicate close relatives (*SI Appendix*, section S6). Also based on fineSTRUCTURE results, we split the Kete into two groups: north (Kete_N, 45 individuals) and south (Kete_S, 50 individuals). We tested for admixture in each DRC group separately using all sampled populations as surrogates, using the All-donors painting and the DRC-all-world dataset. We also tested for admixture in the DRC as a whole using the Non-DRC-donors painting and DRC-all-world and DRC-world datasets, using all non-DRC groups except African Caribbeans in Barbados (ACB) and Americans of African ancestry (ASW) as surrogates, with these two groups excluded because they are recently admixed themselves. Finally, to assess how the DRC samples contribute as admixing sources in other African groups, we also tested each African population for admixture using the DRC-all-world dataset and the All-donors painting, under a separate analysis that additionally excludes DRC as a surrogate. In all instances, we ran GLOBETROTTER as described in *SI Appendix*, sections S9, S12, and S13.

## Supplementary Material

Supplementary File

Supplementary File

Supplementary File
