# Supplementary material for: Genetic legacy of state centralization in the Kuba Kingdom of the Democratic Republic of the Congo
Source: Proc Natl Acad Sci U S A. 2018 Dec 24;116(2):593–8. doi: 10.1073/pnas.1811211115 (PMC6329964; doi:10.1073/pnas.1811211115)
Supplement: Supplementary File [file pnas.1811211115.sapp.pdf]

Supplementary Figures and Tables

The Genetic Legacy of State Centralization in the Kuba Kingdom of the Democratic Republic of Congo

Contents

S1 Description of the historical background **2**
S1.1 History prior to the formation of the Kuba Kingdom 2
S1.2 The formation of the Kuba Kingdom 2
S1.3 A description of the Kuba Kingdom 3
S1.4 The Kuba Kingdom during the colonial and post-colonial periods 4

S2 Description of DRC dataset **6**

S3 Analysis of uni-parental marker systems **9**

S4 Inferring within group genetic diversity **13**
S4.1 analyses with fastIBD 13
S4.2 analyses with CHROMOPAINTER 14

S5 Genetic distance between DRC groups **17**
S5.1 Haplotype-based measures of genetic distance 17

S6 Clustering individuals within the DRC based on haplotype sharing **24**

S7 Inferring ratios of most recent ancestor sharing between groups **29**

S8 Simulation framework **35**
S8.1 Within-group genetic diversity in simulated populations 35

S9 Inferring and dating admixture within the DRC **37**

S10 Description of global datasets **41**

S11 DRC individuals' ancestry sharing with non-DRC groups **44**
S11.1 Mixture modeling of ancestry proportions 49

S12 Inferring and dating admixture from non-DRC sources into the DRC **52**

S13 Inferring and dating admixture within Africa **56**

www.pnas.org/cgi/doi/10.1073/pnas.1811211115

S1 Description of the historical background

The section aims to provide a background to the known and reported history of the region before, during, and after the formation of the Kuba Kingdom. This includes material from the Supplementary Appendix materials of Lowes et al. (2017). We present the following sections as an assimilation of histories according to scholars of the region. We note however that, as with many regions of Africa, our current understanding is heavily reliant on the evalution of oral histories, which cannot necessarily be supposed as documentary evidence, e.g. see Newbury (2007).

S1.1 History prior to the formation of the Kuba Kingdom

According to existing knowledge, there appears to have been several waves of historical migrations to the area around what would become the Kuba Kingdom. During the medieval period, a group of Mongo people, who would eventually become the Kuba and related peoples, migrated from the northwest and crossed the Sankuru river. The migration has been described by historian Jan Vansina as "part of the general expansion of the southern Mongo...The historian visualizes an expansion over a [broad] front, all along the Sankuru and parts of the lower Kasai. The Leele [Lele] crossed first, then the Bieeng and the Pyaang, then the Ngeende, and then the Bushong" (Vansina, 1978, p.56). Similarly, anthropologist Mary Douglas (1963) writes that the "Lele and Bushong and the other members of the group of tribes known as Kuba...formed the van of a movement of peoples from the north-west...who thrust their way up the Kwa, Kwango, Kwilu and Kasai rivers. The Kuba are the eastern outliers of the movement, and the Lele stand on the southernmost point of their expansion" (Douglas, 1963, pp.9,11).

The Mongo migration included many different groups, including the Bushong, Ngeende, Pyang, Bulaang, Bieeng, and Lele. Five of these groups, the Bushong, Ngeende, Pyang, Bulaang, Bieeng, would later form the Kuba Kingdom and comprise what Vansina (1978, p.5) refers to as the "central Kuba". There were also other groups present in the Kingom which were not part of the same migration. He calls these the "peripheral Kuba". Despite originating from the same historical migration as the "central Kuba", the Lele were never incorporated into the Kingdom.

Commonalities between the oral histories of the central Kuba and the Lele reflect their common ancestry. For the central Kuba, the world began with Mboom, who had a son named Woot. Woot committed incest with his sister Mweel, resulting in their banishment from the village and their migration. Woot and Mweel had a series of children who are the progenitors of the central Kuba and Lele (Vansina, 1978, p.32). The origin story of the Lele is very similar. As explained by Torday (1925, pp.127-128), Woot had a son named Nyimi Lele with his sister, resulting in "such an outcry of indignation amongst the people that Woto [Woot] had to order his son to leave the country. With his adherents, Nyimi Lele travelled until he reached the River Katembo (the Loange) which he followed, and there he founded the nation of the Bashilele (sons of Lele) and his descendants were their chiefs" (also see Wharton (1927, p.66)).

S1.2 The formation of the Kuba Kingdom

The Kuba Kingdom was formed in the early 17th century. The Kingdom, once established, expanded to the boundaries defined by surrounding rivers. The Kingdom was delineated by the Kasai river in the West, the Sankuru river in the North, and the Lulua river in the South. The formation of the Kingdom is attributed to an outsider, named Shyaam, who first made himself the chief of the Bushong by overthrowing the existing chief before uniting the other groups under his rule. Although not known with any certainty, his ability to form the Kingdom was potentially due to several idiosyncratic factors. First, he had travelled widely, and thus had

access to long-distance trade networks. As a result, he was able to introduce a number of new technologies to the area, including many new world crops (e.g. tobacco, cassava, and maize) as well as innovations (e.g. how to make raffia cloth and palm wine). Second, he was considered a magician and medicine man. Finally, he introduced many institutional innovations, including initiation rituals, ceremonial knives, the royal charm, and the belt of office (Vansina, 1978, pp.59-65).

The people on the other sides of the rivers that delineated the Kuba Kingdom were never unified under a centralized state (Vansina, 1966b). For example, the Luluwa, one of the largest ethnic groups in Kananga and in our sample, were organized into small chiefdoms. Martens (1980, p.40) explains that "The Lulua were organized into small chiefdoms or independent clan groupings with the leader usually being a senior member of the clan." This organization was typical for the groups surrounding the Kuba such as the Chokwe to the southwest (McCulloch, 1951), of the Luntu and the Songe (Vansina, 1966a, pp.168-169), and the Sala Mpasu to the South (Pruitt, 1973). Other ethnic groups, like the Lele, were even less centralized. Douglas (1963) notes that they lacked chiefs with any authority, let alone professional bureaucrats, judges, or policemen.

Other than the Kuba Kingdom, there were no other states in the region. The closest states were distant and were: the Luba Kingdom, hundreds of miles to the southeast in modern-day Katanga, the Lunda Kingdom, also hundreds of miles to the south in northern Angola, and the Kanyok Kingdom (Reefe, 1981; Bustin, 1975; Yoder, 1992). Oral histories of these states suggest that they developed independently. Our sample does not contain individuals whose ancestors lived in any of these other three kingdoms. While our sample does contain individuals who report their ethnicity as 'Luba', these Tshiluba-speaking individuals descend from migrants from Luba territories who formed separate communities (outside of the Kuba Kingdom) that were not incorporated into a centralized state. An example is the stateless Coofa, who descend from such Luba migrants. Today, in Kananga, descendants of the stateless migrant Luba are known as 'Luba', while descendants of the Luba Kingdom are known as 'Lubakat', a combination of the words 'Luba' and 'Katanga'.

S1.3 A description of the Kuba Kingdom

The territory of the Kuba Kingdom was divided into nine provinces that were themselves subdivided into counties, each of which had a head chief (Vansina, 1978, p.128). The Kingdom had executive councils, professional bureaucracies, a military, and police forces. The executive, apart from the king, comprised a system of title holders, called *kolm*. There were 120 distinct titles in the late 19th century. Though some of these titles were reserved for members of 18 aristocratic clans, the majority was appointed meritocratically such that status was achievement-based.

The King interacted with four main councils. The most significant of these was the *ishyaaml*, which did not include the king. It had fixed rules for establishing a quorum and a fixed membership that included: the *kikaam*, the highest official in the bureaucracy; the *kum ashin*, the provincial governors; and the *mbyeemy*, the ritualist of the court. The *ishyaaml* met frequently and could veto the kings orders and edicts. If a veto occurred, the issue went to another council, the *mbok ilaam*, which met in a particular square deep within the palace and included all *kolm* as members. This council dealt with current affairs and had procedures for reaching a compromise when conflicts arose between the king and the *ishyaaml*. The other two main councils were the *ibaanc* and the *iyoot*, both of which met only under special circumstances. The *iyoot*, for instance, was connected to warfare, and it was where the king informed the *kolm* about military events and decisions (Vansina, 1978, pp.145-152).

The Kingdom had a well-developed highly functioning administration. For example, it

levied annual taxes on all villages in its domain. It also required corvée labor, for example, to build and maintain the capital city, as well as a kingdom-wide system of roads and bridges. It also regulated economic activity and markets. Overall, the Kuba Kingdom was among the most sophisticated pre-colonial states in all of sub-Saharan Africa. At time of colonization, the central administration in the capital of Mushenge had more than 100 full-time officials (Vansina, 2010, p.46) (Torday and Joyce, 1910, pp.53-56). Law enforcement was also formally institutionalized. In 1892, the police force in the Bushong territory consisted of 40 men, who were led by the son of the King (Sheppard, 1917, pp.99-100,139-140), and each village in the Kingdom had two policemen (Vansina, 1971, p.138).

The administration was a meritocracy. Any free man had the ability to become a *kolm* (Vansina, 1978, p.132). Although the Kingdom featured social stratification, with a class of bureaucrats, an urban elite, artisans, traders, village farmers, and general laborers, mobility was very high (Vansina, 1978, pp.166-167).

Another distinctive feature of the Kuba Kingdom was its elaborate judicial system, which included trial by jury and appellate courts. In this system, there were two judicial forums, the *moots* and the courts, as well as different levels: the clan, the village, the chiefdom, and the Kingdom. Minor disputes were dealt with by *moots*, informal assemblies of relatives and kinsmen who heard evidence and arbitrated in public meetings. More serious offenses went to courts, in which a panel of judges with particular expertise or experience in the crime or dispute under consideration, would be selected. From the basic court, appeals could be made to another court headed by a particular *kolm*, called a *baang*. From this court, appeal could be made to yet a higher court presided over by the *kikaam*, the highest bureaucrat in the Kingdom. A final appeal could then be made to what Vansina (1971, p.138) describes as the "supreme court," where the senior members of the 18 aristocratic clans took part and the King acted as a spectator, ready to grant a stay of execution if necessary. All cases of murder in the Kingdom went directly to the supreme court.

The capital of Mushenge was the cultural and economic center of the Kingdom. In 1892, the population is estimated to have been between 5,000 and 10,000 people (Vansina, 1978, p.169). The population of the capital included those with administrative positions, as well as an urban elite.

Within the Kingdom trade flourished. Raffia squares (*mbal*) and later cowrie shells were used as a local currency. Raw materials, like raw cloth and raw iron, were sent to the capital and other urban centers where they were then transformed into finished products. This division of production resulted in increased efficiency and greater economic productivity (Vansina, 1978, pp.184-186).

S1.4 The Kuba Kingdom during the colonial and post-colonial periods

The first European contact with the Kingdom occurred in 1885 when Ludwig Wolf, a doctor who was second in command on the mission of explorer Hermann Wissman, visited the Kingdom. However, Wolf never made it much beyond the fringe of the Kingdom and it was an Afro-American Presbyterian minister named William Sheppard who first reached the capital city of Mushenge in 1893. After Sheppard, there is a long series of visitor accounts (e.g. Verner (1903)), culminating in the first full-fledged ethnography/history by Torday and Joyce (1910), who visited the region in 1907.

During the period of the Congo Free State (1885-1908), all parts of Kasai were part of a concession granted to the Compagnie de Kasai, which engaged in intense and brutal rubber collection. The impact of the Compagnie de Kasai seems to have been fairly uniform across Kasai in general (Martens, 1980). In 1910, after the Congo had become a Belgian colony, a

system of indirect rule was established whereby traditional political institutions functioned as the lowest level of government of the colony. In the Kuba Kingdom, this form of indirect rule was very similar to colonial rule elsewhere in Congo. The Kuba were subject to the usual colonial impositions, such as forced crop production, head taxes, and labor services (Vansina, 2010, p.208).

Unlike other large kingdoms on the African continent, the Kuba Kingdom remained intact during and after colonial rule. Thus, many of the Kingdoms pre-colonial institutions persisted until independence. Less information is available on the post-colonial period. After an ill-fated attempt at secession between 1960 and 1962 (Dedeken, 1978), the Kasai region fell into sustained decline during the regime of Joseph Mobutu from 1965-1997. Throughout this time and until today the Kuba Kingdom has persisted, though its traditional political structures were negatively affected during this period, as was the case in the rest of the DRC.

S2 Description of DRC dataset

Our sample comprises individuals living in Kananga, the capital of the local province, Kasai Occidental. Our sample included participants from 2,144 households from which individuals were selected. In order to ensure sufficient participants from the groups most relevant to the Kuba Kingdom, individuals from this dataset were selected based on (i) having an origin village or birth village in Mweka, a district whose borders are nearly identical to that of the historical Kuba Kingdom; (ii) having an origin or birth village in the districts contiguous to Mweka; or (iii) having an origin village or birth village in other districts, but who belong to an ethnic group represented in Mweka, such as the Kete, Kuba or Lele.

Saliva samples were collected from the selected individuals during the dry season (June to August) over three seperate collections, which took place in association with local health clinics in Kananga. These collections were lead by a team of Congolese enumerators, who visited participants three times, after the initial screening survey. The enumerators involved in direct sample collection were from the same city, spoke the same languages (usually Tshiluba or French), and were familiar with local ethnic categories, which in the region of Kanaga are widely used. Ethnicity was self-reported and consistency of self-identification over the three visits was checked for each individual before inclusion of that individual's saliva in the genetic analyses. We note that, importantly, in this region the concept of ethnicity is synonymous with language/dialect, helping to ensure robustness in each participants self-reported ancestry. For example, the Kuba speak a different dialect of language than the Lele. The Lele speak Bashilele and the Kuba Bakuba. In general, this is the case amongst our sampled groups.

DNA from the 2014 collection was genotyped by ftDNA (www.familytreedna.com) genotyping service. DNA from two subsequent collections (2015) were genotyped by 23andMe (www.23andme.com) as part of their global sampling initiatives. Participants gave their informed consent for their genetic material to be used as part of the reference databases curated by 23andMe. They were not themselves taking an ancestry test or anticipating receiving individual feedback on ancestry or relatedness.

Following genotyping, all data was quality checked in PLINK(Chang et al., 2015) to exclude SNPs with a missing genotype rate of >10%, minor allele frequency of <1%, and related individuals were excluded based on a PIHAT coefficient >0.2. The final dataset merge, which we term the *DRC-only* dataset, comprised 693 individuals from 27 labelled ethnic groups genotyped across 247,460 SNPs. The geographic distance between sampled individuals is provided in Table S2. Additional data from publicly available sources that were included in the global dataset merges (*DRC-world* and *DRC-all-world*) are described in Section S10.

DRC Dataset			
Groups	N.inds	Kuba sub-groups	N.inds
Luluwa	198(49)/71	Bushong	47(13)
Kuba	101(24)/16	Ngeende	19(4)
Luntu	58(12)/33	Pyang	11(2)
Lele	52(20)/15	Bambengi	3(2)
Kete (Combined)	95(12)/56	Ikongo	3
Kete_S	50(1)/25	Kalambamba	3
Kete_N	45(11)/31	Bayisambu	2
Bindi	44(12)/22	Bieeng	2(1)
Luba	33(7)/20	Bolemba	2(1)
Dekese	20(5)/9	Batua	1
Songe	17(2)/11	Bulendo	1
Tetela	16(4)/5	Imanya	1
Mbala	12(1)/10	Kindjale	1
Tshokwe	9(1)/9	Lukengu	1
Sala	8/7	Malongo	1
Kongo	5/5	Malumalu	1
Pende	5/5		
Nyoka	4/4		
Mfuya	3/2		
Nyambi	3/3		
Lunda	2/2		
Bemba	1/1		
Bena_Konji	1/1		
Bunde	1/1		
Dinga	1(1)/1		
Kusu	1/1		
Lubakat	1(1)/1		
Mongo	1/1		
Moyisambo	1/1		

Table S1: Description and number of QC'ed individuals (N.inds) for the *DRC-only* dataset. The number of sampled individuals is provided, with those genotyped by ftDNA and used in the *DRC-world* dataset given in brackets. All other individuals were genotyped by 23andMe. "/X" provides the number of sampled sub-groups. For the Kuba, we provide sub-group information for all but two individuals that were missing this information.

Group	N.inds	Distance (5-95%) (km)
Luluwa	198	84.0 (11.3-184.8)
Kuba	101	63.8 (6.19-195.1)
Luntu	58	54.4 (6.2-113.3)
Lele	52	105.3 (4.1-433.8)
Kete (Combined)	95	160.6 (6.1-331.4)
Kete_S	50	62.6 (8.3-137.3)
Kete_N	45	41.7 (0.1-84.2)
Bindi	44	79.2 (3.6-242.3)
Luba	33	95.0 (11.9-279.6)
Dekese	20	66.9 (5.4-121.8)
Songe	17	158.3 (11.5-284.3)
Tetela	16	164.5 (23.1-311.8)
Mbala	12	157.0 (6.2-460.1)
Tshokwe	9	240.3 (40.9-558.7)
Sala	8	98.8 (13.3-298.3)
Kongo	5	501.9 (118.7-891.9)
Pende	5	237.2 (27.2-533.2)
Nyoka	4	91.2 (22.1-167.0)
Mfuya	3	23.3 (6.0-33.7)
Nyambi	3	63.7 (23.3-91.9)
Lunda	2	140.4 (140.4-140.4)

Table S2: Geographic distances in kilometres (km) between members of the same ethnic group, calculated using the Haversine formula assuming the Earth's radius is 6,371km. Mean and 5-95% quantiles across all pairwise comparisons are provided in parentheses. Values are only given for groups with >1 individual.

S3 Analysis of uni-parental marker systems

To test the relative levels of diversity within breeding males and females from each ethnic group, we analysed two uni-parentally inherited marker systems: mitochondrial DNA (mtDNA) passed from mother to offspring, and the non-recombining Y chromosome (NRY) passed from father to son. Across the 542 individuals genotyped by 23andMe, 405 SNPs were genotyped on the mtDNA and 1149 SNPs were genotyped on the NRY. Of these, 540 had reported group identity and gender; we assessed the accuracy of sex assignments with PLINK 1.9 (Chang et al., 2015) using the "–check-sex" option. Three individuals were found to have a reported gender that did not match their sex assignment: one Luluwa, one Tshokwe, and one Luba. These individuals were removed from further analyses of uni-parental markers, leaving a final dataset of 537 individuals (270 males, 267 females). Any group, defined by self-declared ethnic identity, with fewer than 10 individuals were also removed from further analysis, resulting in 213 individuals across six groups.

mtDNA haplogroups were assigned to all individuals based on phylotree (van Oven, 2015) implemented in HaploGrep v2 (Weissensteiner et al., 2016) and are provided in Table S3. NRY haplogroups were assigned to all male individuals using a maximum likelihood approach incorporating the Y chromosome consortium NRY phylogenetic tree (Karafet et al., 2008), implemented in the software Yfitter (Jostins et al., 2014), and are provided in Table S4. Nei's gene diversity (Nei, 1987) and pairwise F_{ST} genetic distances (Reynolds et al., 1983) based on NRY and mtDNA haplogroup frequencies were estimated using Arlequin version 3.1 (Excoffier et al., 2005) and are given in Table S5 and Figure S1.

To the level of resolution afforded by our typed SNPs, our results show that mtDNA diversity was relatively similar among all groups (range of 0.955-0.974), with the lowest value observed in Luntu and the highest in both Bindi and Lele. In contrast, NRY diversity was not evenly distributed among the groups, with Lele showing a nearly two-fold reduced diversity (h=0.281) relative to the group with the second lowest diversity (Kuba; h=0.551), and with an average across all non-Lele groups equal to 0.628. This clear contrast of maternal and paternal lineage diversity in Lele is consistent with fewer males contributing genetically to the next generation, which may be consistent with the practice of polygamy in this community (Douglas, 1997, 1963).

mtDNA haplogroup	Bindi	Kete	Kuba	Lele	Luluwa	Luntu
non-L		2	1	1		1
L0a	1	4	5	7	4	1
L0a2		4	5	1	6	
L0a2a2a1	3	2	1		2	
L0a'g		1	1		1	
L0d1a					1	
L1b1	3	5	1		12	4
L1c		1				1
L1c1		1	1	2		
L1c1'2'4'5'6		1	2			
L1c1'2'4'6	2	5	7	3	9	4
L1c1a2		1			2	
L1c1a2a					1	
L1c1d		1	2		2	1
L1c2a3a			2		7	3
L1c3a	2	3			4	1
L1c3b1		1			1	
L1c3b1a		1	1			2
L2	1		2		6	
L2a1		1	1	2	4	2
L2a1a	1	5	4		10	1
L2a1a3c		4	1		2	
L2a1c		10	5	1	5	2
L2a1d						1
L2a1l1a	1	5	1	2	8	
L2a1n				1		
L2a2'3'4		1				1
L2b'c	2	4	2	1	2	
L2c2a		1	1	1		
L2c2b1b	1				1	
L2d		1	2			
L3b	2	1	1	2	1	3
L3b1a3	3		5	2	9	3
L3b1a8	1				1	
L3b3					1	
L3d1'2'3'4'5'6		1	1			
L3d1a1a	1		2	2	4	
L3d3a				1	4	
L3d3a1a		1	1			
L3d3b					1	
L3e1	1	2	6	2	7	6
L3e1a1	1	2		1	2	
L3e1a2				1		
L3e1b2	1	1	2			
L3e2a1a			1	1		
L3e2a1b		3			1	
L3e2b	2	5	7	2	16	3
L3e2b1			1			
L3e2b1a2		1	1			1
L3e3b	2	4	3	2	10	3
L3f1b	2	3	2	2	6	3
L4b1a	2	2	2			
Total	35	91	83	40	153	47

Table S3: mtDNA haplogroup frequencies in all DRC groups analysed as part of the cohort genotyped by 23andMe described in Table S1.

NRY haplogroup	Bindi	Kete	Kuba	Lele	Luluwa	Luntu
B	2				1	1
B2a1			2		2	
E1b1a		2	2		1	2
E1b1a7*		1	1		3	
E1b1a7a	7	17	10	3	32	6
E1b1a8a	10	21	28	16	33	4
E2					1	
E2b		1	1			1
R1		1				
Total	19	43	44	19	73	14

Table S4: NRY haplogroup frequencies in all DRC groups analysed as part of the cohort genotyped by 23andMe described in Table S1.

NRY						
	Bindi	Kete	Kuba	Lele	Luluwa	Luntu
Bindi	0					
Kete	<0.001	0				
Kuba	0.00483	0.02202	0			
Lele	0.11501	0.13268	0.01769	0		
Luluwa	<0.001	0.0041	0.04893	0.1672	0	
Luntu	0.00365	<0.001	0.10049	0.31319	<0.001	0
mtDNA						
	Bindi	Kete	Kuba	Lele	Luluwa	Luntu
Bindi	0					
Kete	0.00073	0				
Kuba	0.00162	<0.0001	0			
Lele	<0.0001	<0.0001	<0.0001	0		
Luluwa	<0.0001	0.00491	<0.0001	0.00165	0	
Luntu	<0.0001	0.01005	0.00223	0.00201	0.00079	0

Table S5: Pairwise F_{ST} genetic distances based on NRY haplogroup frequencies (top) and mtDNA haplogroup frequencies (bottom). F_{ST} values highlighted in grey are statistically significant at the 5% level.

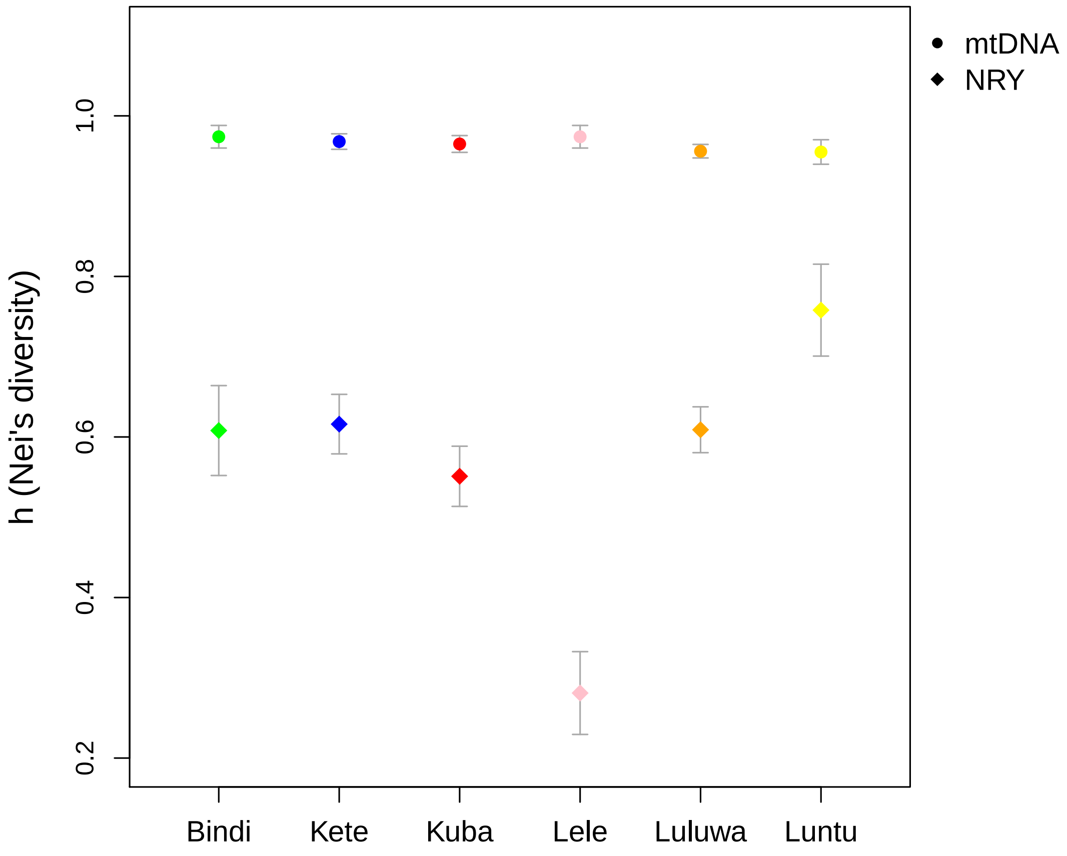

Figure S1: Nei's diversity index scores for each of the DRC ethnic groups based on mtDNA (circle) and NRY (diamond) haplogroup frequencies. Standard errors are provided as grey bars.

S4 Inferring within group genetic diversity

S4.1 analyses with fastIBD

To explore within-group genetic diversity, we applied fastIBD (Browning and Browning, 2011) using BEAGLE v.3.3.2.34 to infer the pairwise IBD fraction shared between each pairing of all individuals separately within each of the seven labelled ethnic groups with >30 individuals (Kuba, Bindi, Kete (also Kete_N, Kete_S), Lele, Luba, Luluwa, Luntu), using the *DRC-only* dataset. For each chromosome of each cluster, we ran 10 independent fastIBD runs using an IBD threshold of 10^{-10} for every pairwise comparison of individuals.

Using this approach, individuals belonging to the Kuba ethnic group showed the shortest average length of tracts shared IBD within individuals of their group, indicative of relatively higher levels of within-group genetic diversity (main text Figure 2a, Table S6).

Group	FIBD		
	Median	5% Quantile	95% Quantile
AdMixPop	0.00246	0.00006	0.01149
MixPop	0.00186	0.00006	0.00974
Kuba	0.00242	0.00008	0.01153
Kete(Combined)	0.00276	0.0001	0.01265
Lele	0.00309	0.00018	0.01561
Luluwa	0.00317	0.00006	0.01564
Kete_S	0.00331	0.00024	0.01234
Luba	0.00369	0.00042	0.01604
Kete_N	0.00396	0.00027	0.01917
Bindi	0.00448	0.00036	0.01756
Luntu	0.00461	0.00038	0.01772

Table S6: FastIBD (Browning and Browning, 2011) inferred average lengths (in cM) of IBD tracts (FIBD) across all pairwise comparisons of individuals for groups with >30 individuals within the DRC as also shown in main text Figure 2a. We also provide results for two simulated populations MixPop and AdMixPop, see Section S8 and main text **Methods**.

For each pairwise comparison of the seven ethnicities, we permuted group labels to assess whether the mean within-group IBD matching across individuals is significantly different between the two groups compared. Table S7 shows the proportion of 1,000 such permutations where the difference in mean within-group IBD matching between the two (artificial) groups is less than that observed in the real data.

Group	AdMixPop	MixPop	Kuba	Kete	Lele	Luluwa	Luba	Bindi	Luntu
**AdMixPop**			1	0.997	1	1	0.996	1	1
**MixPop**			1	0.996	1	1	1	1	1
**Kuba**	0.002	0		0.997	1	1	0.997	1	1
**Kete**	0	0.003	0.003		0.996	1	0.779	1	1
**Lele**	0	0	0	0.004		0.846	0.124	0.799	0.996
**Luluwa**	0	0	0	0	0.154		0.008	0.828	0.893
**Luba**	0.002	0.002	0.003	0.221	0.876	0.992		0.993	0.998
**Bindi**	0	0	0	0	0.201	0.172	0.007		0.844
**Luntu**	0	0	0	0	0.004	0.107	0.002	0.156	

Table S7: Each cell gives the proportion of 1,000 permutations of ethnic label for which the average within-group pairwise IBD sharing (Browning and Browning, 2011) for one ethnic group (column) minus the average within-group pairwise IBD sharing for the other ethnic group (row) is less than the observed such difference in the real (un-permuted) data. Results are also given for simulated populations AdMixPop and MixPop (see Section S8 and main text **Methods**).

S4.2 analyses with CHROMOPAINTER

As a second analysis, we also used CHROMOPAINTER (Lawson et al., 2012) to infer within-group homogeneity, by painting individuals from each label using other individuals with the same label as donors. In particular we inferred the genome-wide average number of haplotype segments (i.e. strings of contiguous SNPs inherited from a recent common ancestor, the ".chunkcounts.out" CHROMOPAINTER output) that the Kuba matched with other Kuba under CHROMOPAINTER, with a higher number indicating greater genetic diversity (i.e. shorter matching haplotype segments among Kuba). We compared the Kuba's segment count to that of other DRC ethnic groups containing >30 sampled individuals. For each comparison of Kuba and another group, we used default values in CHROMOPAINTER and matched for sample size by using $X = \min(n_A - 1, n_B - 1)$ donors, where n_A is the number of sampled individuals from the Kuba and n_B is the number of sampled individuals from group B (Bindi, Kete (combined), Kete_N, Kete_S, Lele, Luba, Luluwa, Luntu). For each comparison, we report the two sample t-test statistic used to evaluate whether the Kuba had a significantly higher average number of total segments. However, the t-test values are for comparison only, as significance is challenging to assess here given the inferred paintings for individuals include overlapping donors and hence are not independent, with permutations impractical given the computational expense of this analysis. Consistent with Figure 2a of the main text and Table S6, these analyses highlight that the Kuba have a higher overall within-group genetic diversity (more counts) within each comparison (Figures S2-S3, S11).

We repeated this CHROMOPAINTER analysis replacing the Kuba with the three Kuba sub-groups for which we had >10 sampled individuals: Bushong ($n\_Bushong$=47), Ngeende ($n\_Ngeende$=19) and Pyang (n_Pyang=11) (Figure S2). The Bushong, the sub-group with the highest sample size, was consistently found to have a higher amount of within-group autosomal genetic diversity in each comparison, reflecting the patterns seen in the full Kuba. This trend is broadly consistent in the Ngeende and Pyang, except for comparisons with the Lele. However, when we sub-sampled the Bushong to match the sample sizes of these two sub-groups, Lele was the only group with greater inferred median diversity, suggesting the Ngende and Pyang sub-groups do not have enough samples for reliable inference here (Figure S3).

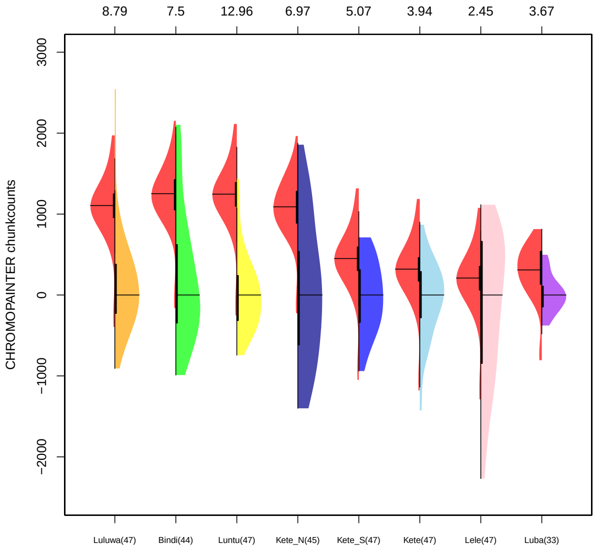

(a) Bushong

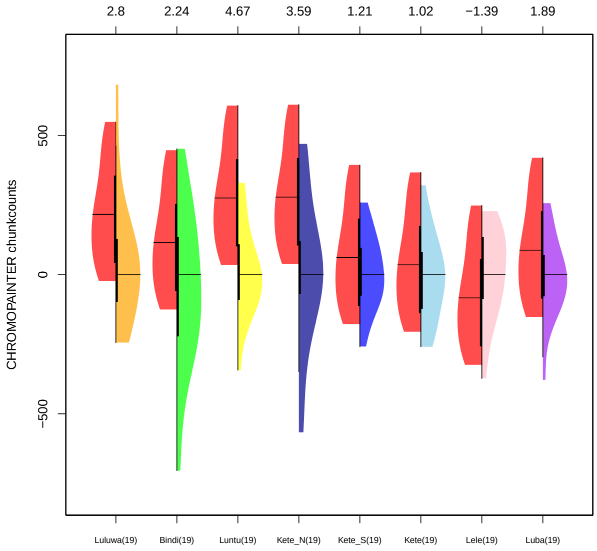

(b) Ngeende

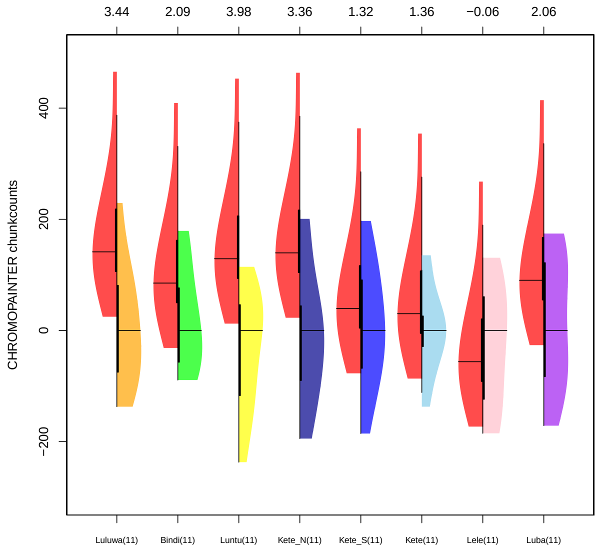

(c) Pyang

Figure S2: CHROMOPAINTER inferred number of DNA segments that individuals share with other individuals from their group, which is inversely proportional to DNA segment size, for Kuba sub-groups (in red): Bushong (a), Ngeende (b) and Pyang (c) in comparison to DRC groups with >30 individuals. For each comparison the number of individuals used is matched between the two populations being compared; this number is provided in brackets on the x-axis. Two sample t-test statistics (Kuba - X) are provided at top. Horizontal lines provide the median estimates.

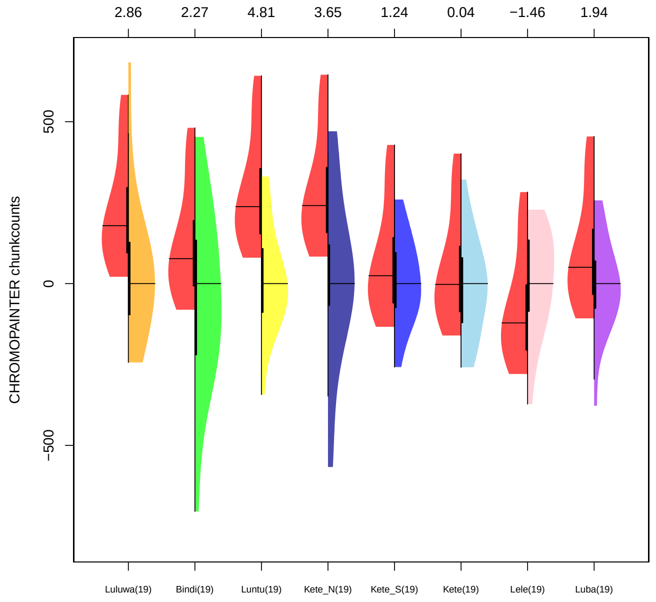

(a) Bushong (19)

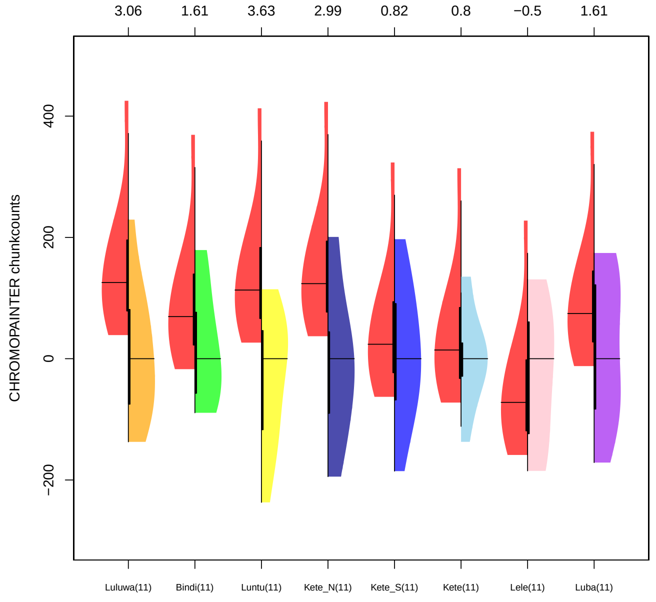

(b) Bushong (11)

Figure S3: CHROMOPAINTER inferred number of DNA segments that individuals share with other individuals from their group, which is inversely proportional to DNA segment size, among Bushong (a Kuba sub-group) when sub-sampling individuals to match sample sizes for (a) Ngende (19 individuals) and (b) Pyang (11 individuals). Two sample t-test statistics (Kuba - X) are provided at top. Horizontal lines provide the median estimates.

S5 Genetic distance between DRC groups

F_{ST} between each pair of ethnic groups in the *DRC-only* merge (Table S8) was calculated using the Weir and Cockerham formula (Cockerham and Weir, 1986) implementation in PLINK v1.9 (Chang et al., 2015). F_{ST} demonstrates that genetic structure amongst sampled DRC groups is extremely subtle (range 0-0.0018), comparable to those seen among people from different geographic regions of the United Kingdom(Leslie et al., 2015). This could reflect the close geographic proximity of individuals' reported origins (main text Figure 1, Table S2).

**Group**	**Sala**	**Tshokwe**	**Mbala**	**Tetela**	**Songe**	**Dekese**	**Luba**	**Bindi**	**Kete_N**	**Kete_S**	**Kete**	**Lele**	**Luntu**	**Kuba**	**Luluwa**
**Sala(8)**	0	0	0	0	0	0	0	0	0	0	0	0	0	0	0
**Tshokwe(9)**	0	0	0	0	0	0	0	0	0	0	0	0	0	0	0
**Mbala(12)**	0	0	0	0	0	0	0	0.0007	0.0004	0	0	0.0002	0.0006	0	0.0005
**Tetela(16)**	0	0	0	0	0	0	0.0004	0.0011	0.0011	0.0007	0.0006	0.0006	0.001	0.0003	0.0011
**Songe(17)**	0	0	0	0	0	0.0002	0	0.0006	0.0006	0.0004	0.0002	0.0005	0.0003	0	0.0002
**Dekese(20)**	0	0	0	0	0.0002	0	0.0008	0.0015	0.0014	0.0009	0.0009	0.0008	0.0014	0.0005	0.0015
**Luba(33)**	0	0	0	0.0004	0	0.0008	0	0.0007	0.0008	0.0006	0.0004	0.001	0.0001	0.0001	0.0001
**Bindi(44)**	0	0	0.0007	0.0011	0.0006	0.0015	0.0007	0	0.0014	0.0013	0.0011	0.0018	0.0009	0.0009	0.0011
**Kete_N(45)**	0	0	0.0004	0.0011	0.0006	0.0014	0.0008	0.0014	0	0.0011	0	0	0	0	0
**Kete_S(50)**	0	0	0	0.0007	0.0004	0.0009	0.0006	0.0013	0.0011	0	0	0	0	0	0
**Kete(95)**	0	0	0	0.0006	0.0002	0.0009	0.0004	0.0011	0	0	0	0	0	0	0
**Lele(52)**	0	0	0.0002	0.0006	0.0005	0.0008	0.001	0.0018	0	0	0	0	0	0	0
**Luntu(58)**	0	0	0.0006	0.001	0.0003	0.0014	0.0001	0.0009	0	0	0	0	0	0	0
**Kuba(101)**	0	0	0	0.0003	0	0.0005	0.0001	0.0009	0	0	0	0	0	0	0
**Luluwa(198)**	0	0	0.0005	0.0011	0.0002	0.0015	0.0001	0.0011	0	0	0	0	0	0	0

Table S8: Mean pairwise F_{ST} between each pair of ethnic groups in the *DRC-only* merge. Only groups with >5 individuals were included. The total number of individuals in each group is shown in brackets in column 1.

S5.1 Haplotype-based measures of genetic distance

To explore patterns of shared ancestry between DRC samples further, and with increased resolution, we used CHROMOPAINTER (Lawson et al., 2012) to paint all recipient groups using all other DRC groups (*DRC-only* dataset) as donors. We refer to this as the **All-donors** analysis (see main text **Methods**).

We initially estimated the CHROMOPAINTER mutation (θ) and switch rate (Ne) parameters across 4 chromosomes 1,4,15 and 22 on every individual. This gave estimates of $\theta = 0.000398$ and Ne = 113.155. CHROMOPAINTER was then run with these fixed estimates across all autosomes to infer a haplotype sharing profile for each individual by measuring the amount of DNA contributed from each donor group. In particular this profile is a vector of length K, with each element comprising the total proportion of genome-wide DNA that the individual matches to members of each donor group $k \in [1, ..., K]$, with donor groups defined using population labels.

As well as using haplotype information, we additionally ran CHROMOPAINTER using the unlinked mode (Lawson et al., 2012), which considers all SNPs as independent, to explore how much information is gained through using haplotype information in this dataset.

We calculated the metric TVD (Leslie et al., 2015) to provide a statistical measure of the amount of difference between populations. TVD is analogous to F_{ST}, but based on the painting profiles inferred using CHROMOPAINTER (Lawson et al., 2012) and hence using haplotype information (i.e. correlations among neighboring SNPs) and relative amounts of matching to multiple groups in order to increase power. In the "unlinked" mode of CHROMOPAINTER, using TVD differs from F_{ST} by measuring the difference in how the two compared groups match to other populations.

In brief, under the **All-donors** analysis (see **Methods**), let f_k^X be the genome-wide proportion of DNA that recipient DRC individual X copies from donor group (i.e. group label) $k \in [1, ..., K]$ as inferred by CHROMOPAINTER. Then to compare the painting profiles between any two DRC individuals or two DRC groups X and Y, TVD_{XY} is calculated as:

$$TVD_{XY} = 0.5 \sum_{k=1}^{K} |f_k^X - f_k^Y|. \tag{S1}$$

We compare TVD under the CHROMOPAINTER "unlinked" versus "linked" approaches in Figure S4 and Table S9. Similarly to F_{ST}, output from the CHROMOPAINTER "unlinked" approach under the **All-donors** analysis struggles to elucidate strong genetic structure. However the incorporation of haplotype information increases the resolution to resolve different labelled groupings (upper triangle of Figure S4) relative to the "unlinked" approach (lower triangle of Figure S4), with permutation based p-values testing whether group labels are exchangable provided for each approach in Table S10. Despite a relatively low number of SNPs in this merge (247,460 SNPs), this analysis (*All-donors*) indicates there is still enough information present for haplotype-based techniques to be valuable, with CHROMOPAINTER inferring matching haplotype segments averaged across all DRC people to contain 35.9 contiguous SNPs.

A look at the lower right triangle of Figure S4 suggests that, in general, individuals look more genetically similar to other individuals with the same group label (note the lighter colors along the diagonal). In addition, Luntu and Luluwa look somewhat genetically similar to each other (though still significantly different) and relatively more genetically distinct from other ethnic groups, having a higher TVD (darker color) with Lele, Kuba and Kete than the other DRC samples, and perhaps reflecting their geographical proximity to one another and their purported sharing of the same historic expansion event (Vansina, 1966b, 1978; Guthrie, 1971). The Lele have the highest TVD and F_{ST} with most other ethnic groups, notably Luntu and Luluwa, perhaps indicative of a lack of gene flow between Lele and other DRC groups and/or drift due to bottleneck and genetic isolation effects. This is consistent with geography, as Lele are situated to the Western side of the Kasai river, and with politics and anthropology, as the Lele were excluded from the Kuba Kingdom (Vansina, 1978; Douglas, 1997; Vansina, 2010; Acemoglu and Robinson, 2012; Douglas, 1963).

Interestingly we find the mean TVD of each DRC group is often lower when compared to Kuba individuals as opposed to when compared to other sampled DRC groups. This is consistent with a relative lack of isolation between the Kuba and other neighbouring groups in the region (Figure S5-S6), as we explore further in the subsequent sections.

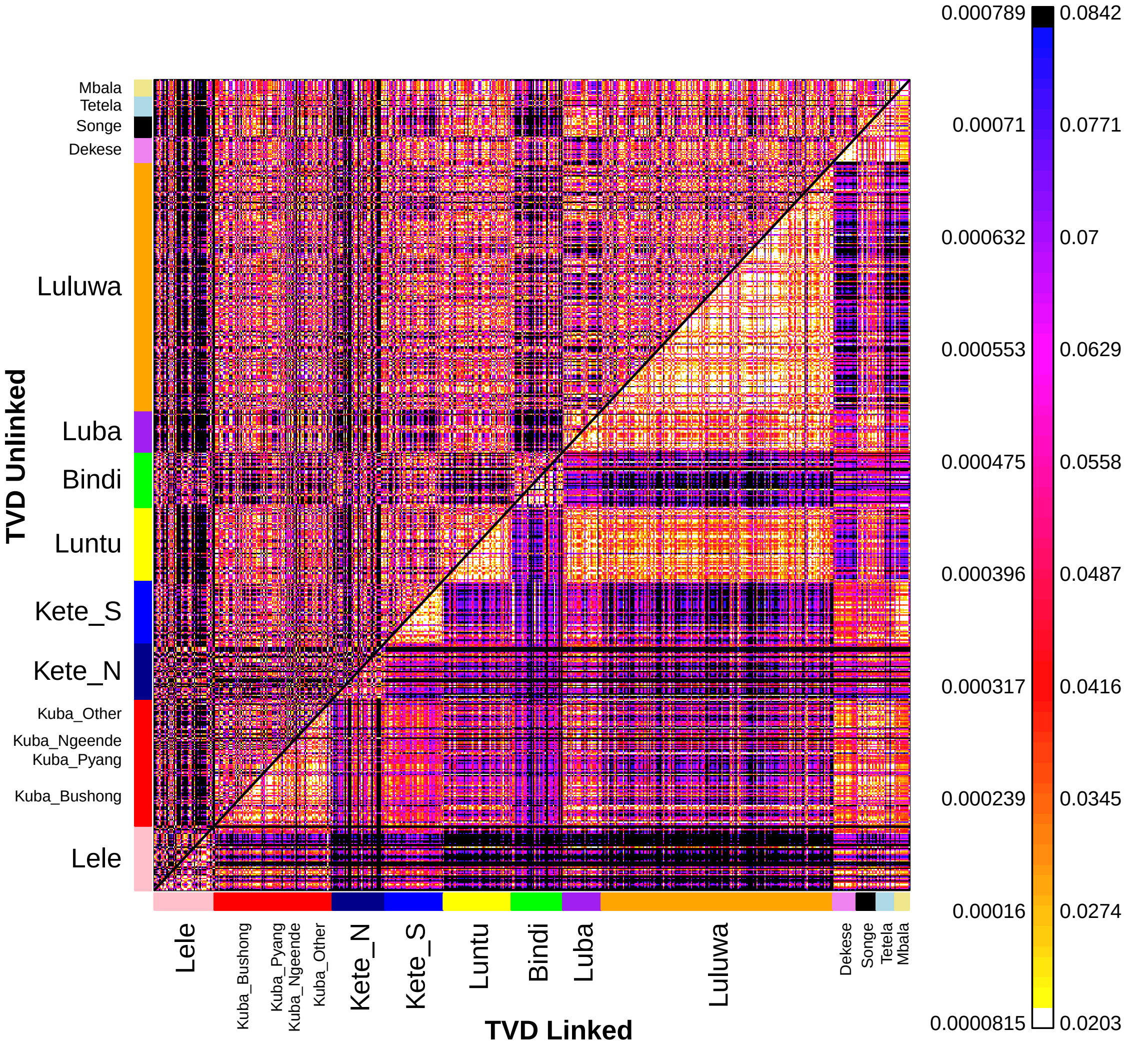

Figure S4: TVD distance matrix based on CHROMOPAINTER inference using the **All-donors** analysis for the *DRC-only* dataset. Only ethnic groups with >10 individuals are included, with individuals ordered by their ascribed ethnicity. The upper triangle provides TVD between patterns of allele frequency sharing, i.e. when CHROMOPAINTER is run under the unlinked model, with the scale provided on the left of the color bar. The lower triangle provides the TVD between patterns of haplotype sharing when CHROMOPAINTER is run under the linked model, with the scale provided on the right of the color bar. A lighter color implies more genetic similarity.

TVD Unlinked

Group	Kuba_All	Kuba_Bushong	Kuba_Ngeende	Kuba_Pyang	Kuba_Other	Lele	Kete_N	Kete_S	Luntu	Bindi	Luba	Luluwa	Dekese	Songe	Tetela	Mbala
Kuba_All	0.0005(0.0004-0.0008)					0.0009(0.0005-0.0013)	0.0007(0.0004-0.0013)	0.0005(0.0004-0.0007)	0.0004(0.0004-0.0006)	0.0006(0.0004-0.0011)	0.0005(0.0004-0.0006)	0.0005(0.0004-0.0007)	0.0004(0.0004-0.0006)	0.0005(0.0004-0.0006)	0.0005(0.0004-0.001)	0.0004(0.0004-0.0005)
Kuba_Bushong		0.001(0.001-0.001)	0.001(0.001-0.001)	0.001(0.001-0.001)	0.001(0.001-0.001)	0.001(0.001-0.001)	0.001(0.001-0.001)	0.001(0.001-0.001)	0.001(0.001-0.001)	0.001(0.001-0.001)	0.001(0.001-0.001)	0.001(0.001-0.001)	0.001(0.001-0.001)	0.001(0.001-0.001)	0.001(0.001-0.001)	0.001(0.001-0.001)
Kuba_Ngeende		0.001(0.001-0.001)	0(0-0.001)	0(0-0.001)	0(0-0.001)	0(0-0.001)	0(0-0.001)	0.001(0-0.001)	0(0-0.001)	0.001(0-0.001)	0(0-0.001)	0(0-0.001)	0(0-0.001)	0(0-0.001)	0(0-0.001)	0(0-0.001)
Kuba_Pyang		0.001(0.001-0.001)	0(0-0.001)	0(0-0.001)	0(0-0.001)	0(0-0.001)	0(0-0.001)	0(0-0.001)	0(0-0.001)	0.001(0-0.002)	0.001(0-0.001)	0(0-0.001)	0(0-0.002)	0(0-0.0002)	0(0-0.00025)	0(0-0)
Kuba_Other		0.001(0.001-0.001)	0(0-0.001)	0(0-0.001)	0(0-0.001)	0(0-0.001)	0(0-0.001)	0(0-0.001)	0(0-0.001)	0.001(0-0.001)	0(0-0.001)	0(0-0.001)	0(0-0.001)	0(0-0.001)	0(0-0.001)	0(0-0.001)
Lele	0.0009(0.0005-0.0013)	0.001(0.001-0.001)	0(0-0.001)	0(0-0.001)	0(0-0.001)	0(0-0.001)	0(0-0.001)	0(0-0.001)	0(0-0.001)	0.001(0-0.001)	0.001(0-0.001)	0(0-0.001)	0(0-0.00105)	0(0-0.001)	0(0-0.001)	0(0-0.001)
Kete_N	0.0007(0.0004-0.0013)	0.001(0.001-0.001)	0(0-0.001)	0(0-0.001)	0(0-0.001)	0(0-0.001)	0(0-0.001)	0(0-0.001)	0(0-0.001)	0.001(0-0.00185)	0.001(0-0.001)	0(0-0.001)	0(0-0.00105)	0(0-0.001)	0(0-0.001)	0(0-0)
Kete_S	0.0005(0.0004-0.0007)	0.001(0.001-0.001)	0.001(0-0.001)	0(0-0.001)	0(0-0.001)	0(0-0.001)	0(0-0.001)	0.001(0-0.001)	0(0-0.001)	0.001(0-0.001)	0.001(0-0.001)	0(0-0.001)	0.001(0-0.001)	0(0-0.001)	0(0-0.001)	0(0-0.001)
Luntu	0.0004(0.0004-0.0006)	0.001(0.001-0.001)	0(0-0.001)	0(0-0.001)	0(0-0.001)	0(0-0.001)	0(0-0.001)	0(0-0.001)	0(0-0.001)	0.001(0-0.001)	0.001(0-0.001)	0(0-0.001)	0(0-0.00105)	0(0-0.001)	0(0-0.001)	0(0-0.001)
Bindi	0.0006(0.0004-0.0011)	0.001(0.001-0.001)	0.001(0-0.001)	0.001(0-0.002)	0.001(0-0.001)	0.001(0-0.001)	0.001(0-0.00185)	0.001(0-0.001)	0.001(0-0.001)	0.001(0-0.001)	0.001(0-0.001)	0.001(0.001-0.001)	0.001(0.00095-0.001)	0.001(0.0008-0.001)	0.001(0.001-0.001)	0.001(0.001-0.001)
Luba	0.0005(0.0004-0.0006)	0.001(0.001-0.001)	0(0-0.001)	0.001(0-0.001)	0(0-0.001)	0.001(0-0.001)	0.001(0-0.001)	0.001(0-0.001)	0.001(0-0.001)	0.001(0-0.001)	0(0-0.001)	0.001(0-0.001)	0(0-0.001)	0.001(0-0.001)	0.001(0-0.001)	0.001(0.001-0.001)
Luluwa	0.0005(0.0004-0.0007)	0.001(0.001-0.001)	0(0-0.001)	0(0-0.001)	0(0-0.001)	0(0-0.001)	0(0-0.001)	0(0-0.001)	0(0-0.001)	0.001(0.001-0.001)	0.001(0-0.001)	0(0-0.001)	0(0-0.00105)	0(0-0.001)	0(0-0.001)	0(0-0.0005)
Dekese	0.0004(0.0004-0.0006)	0.001(0.001-0.001)	0(0-0.001)	0(0-0.002)	0(0-0.001)	0(0-0.00105)	0(0-0.00105)	0.001(0-0.001)	0(0-0.00105)	0.001(0.00095-0.001)	0(0-0.001)	0(0-0.00105)	0.001(0-0.001)	0(0-0.001)	0.001(0-0.001)	0.001(0.00055-0.001)
Songe	0.0005(0.0004-0.0006)	0.001(0.001-0.001)	0(0-0.001)	0(0-0.0)	0(0-0.001)	0(0-0.001)	0(0-0.001)	0(0-0.001)	0(0-0.001)	0.001(0.0008-0.001)	0.001(0-0.001)	0(0-0.001)	0(0-0.001)	0(0-0.001)	0(0-0.001)	0(0-0.001)
Tetela	0.0005(0.0004-0.001)	0.001(0.001-0.001)	0(0-0.001)	0(0-0.00025)	0(0-0.001)	0(0-0.001)	0(0-0.001)	0(0-0.001)	0(0-0.001)	0.001(0.001-0.001)	0.001(0-0.001)	0(0-0.001)	0.001(0-0.001)	0(0-0.001)	0(0-0.00025)	0(0-0)
Mbala	0.0004(0.0004-0.0005)	0.001(0.001-0.001)	0(0-0.001)	0(0-0)	0(0-0.001)	0(0-0.001)	0(0-0)	0(0-0.001)	0(0-0.001)	0.001(0.001-0.001)	0.001(0.001-0.001)	0(0-0.0001)	0.001(0.00055-0.001)	0(0-0.001)	0(0-0)	0(0-0)

TVD Linked

Group	Kuba_All	Kuba_Bushong	Kuba_Ngeende	Kuba_Pyang	Kuba_Other	Lele	Kete_N	Kete_S	Luntu	Bindi	Luba	Luluwa	Dekese	Songe	Tetela	Mbala
Kuba_All	0.039(0.028-0.067)					0.072(0.057-0.105)	0.066(0.035-0.109)	0.049(0.036-0.066)	0.059(0.038-0.077)	0.068(0.042-0.094)	0.048(0.033-0.073)	0.065(0.036-0.089)	0.041(0.031-0.056)	0.038(0.03-0.059)	0.042(0.031-0.076)	0.043(0.038-0.047)
Kuba_Bushong		0.054(0.041-0.0737)	0.062(0.0549-0.0714)	0.072(0.057-0.089)	0.063(0.0533-0.07385)	0.074(0.05755-0.0909)	0.061(0.044-0.0788)	0.062(0.0459-0.088)	0.072(0.05485-0.09945)	0.091(0.055-0.14125)	0.08(0.0572-0.1014)	0.077(0.053-0.101)	0.073(0.04965-0.13595)	0.062(0.056-0.0746)	0.059(0.05125-0.069)	0.061(0.054-0.0759)
Kuba_Ngeende		0.062(0.0549-0.0714)	0.027(0.0199-0.0515)	0.065(0.0465-0.0825)	0.029(0.01915-0.05595)	0.066(0.04255-0.08435)	0.048(0.037-0.067)	0.054(0.03745-0.0852)	0.059(0.035-0.09345)	0.072(0.03645-0.12425)	0.068(0.0216-0.094)	0.069(0.038-0.09515)	0.057(0.0359-0.11995)	0.036(0.0246-0.0624)	0.037(0.02375-0.06)	0.044(0.0313-0.0687)
Kuba_Pyang		0.072(0.057-0.089)	0.065(0.0465-0.0825)	0.026(0.022-0.0315)	0.065(0.0292-0.08485)	0.032(0.02-0.06845)	0.056(0.0274-0.0826)	0.06(0.03-0.1044)	0.046(0.0227-0.0769)	0.098(0.04205-0.15155)	0.067(0.036-0.096)	0.035(0.023-0.05615)	0.073(0.0317-0.14695)	0.062(0.0302-0.0752)	0.047(0.02525-0.071)	0.042(0.0241-0.05915)
Kuba_Other		0.063(0.0533-0.07385)	0.029(0.01915-0.05595)	0.065(0.0292-0.08485)	0.031(0.023-0.05865)	0.066(0.045-0.0839)	0.049(0.0392-0.068)	0.055(0.03945-0.0851)	0.06(0.037-0.0933)	0.075(0.03845-0.1274)	0.07(0.0252-0.096)	0.07(0.04-0.09515)	0.059(0.0369-0.12295)	0.038(0.0276-0.0636)	0.039(0.02575-0.061)	0.046(0.03375-0.06915)
Lele	0.072(0.057-0.105)	0.074(0.05755-0.0909)	0.066(0.04255-0.08435)	0.032(0.02-0.06845)	0.066(0.045-0.0839)	0.038(0.027-0.0679)	0.059(0.0342-0.0818)	0.062(0.03645-0.1045)	0.05(0.0297-0.07775)	0.098(0.0466-0.15055)	0.064(0.0408-0.0894)	0.041(0.03-0.05915)	0.074(0.03865-0.14595)	0.064(0.0362-0.0754)	0.05(0.0325-0.07075)	0.046(0.0311-0.06025)
Kete_N	0.066(0.035-0.109)	0.061(0.044-0.0788)	0.048(0.037-0.067)	0.056(0.0274-0.0826)	0.049(0.0392-0.068)	0.059(0.0342-0.0818)	0.035(0.025-0.0608)	0.043(0.02545-0.08)	0.053(0.0317-0.0836)	0.071(0.0323-0.12625)	0.069(0.0418-0.0944)	0.062(0.03485-0.08815)	0.05(0.025-0.121)	0.04(0.0288-0.0554)	0.041(0.032-0.052)	0.039(0.02955-0.0598)
Kete_S	0.049(0.036-0.066)	0.062(0.0459-0.088)	0.054(0.03745-0.0852)	0.06(0.03-0.1044)	0.055(0.03945-0.0851)	0.062(0.03645-0.1045)	0.043(0.02545-0.08)	0.05(0.036-0.0832)	0.058(0.0397-0.0866)	0.076(0.03915-0.1291)	0.071(0.047-0.0954)	0.066(0.04-0.09015)	0.057(0.035-0.12395)	0.047(0.038-0.0604)	0.047(0.039-0.05625)	0.045(0.03755-0.06335)
Luntu	0.059(0.038-0.077)	0.072(0.05485-0.09945)	0.059(0.035-0.09345)	0.046(0.0227-0.0769)	0.06(0.037-0.0933)	0.05(0.0297-0.07775)	0.053(0.0317-0.0836)	0.058(0.0397-0.0866)	0.05(0.039-0.06545)	0.087(0.04345-0.1404)	0.072(0.0506-0.0988)	0.048(0.039-0.06315)	0.066(0.04195-0.13595)	0.054(0.039-0.0648)	0.047(0.038-0.05975)	0.044(0.0401-0.04935)
Bindi	0.068(0.042-0.094)	0.091(0.055-0.14125)	0.072(0.03645-0.12425)	0.098(0.04205-0.15155)	0.075(0.03845-0.1274)	0.098(0.0466-0.15055)	0.071(0.0323-0.12625)	0.076(0.03915-0.1291)	0.087(0.04345-0.1404)	0.048(0.036-0.07755)	0.096(0.068-0.1164)	0.103(0.07155-0.1273)	0.063(0.03795-0.08585)	0.064(0.0556-0.089)	0.074(0.0605-0.09475)	0.074(0.05865-0.10225)
Luba	0.048(0.033-0.073)	0.08(0.0572-0.1014)	0.068(0.0216-0.094)	0.067(0.036-0.096)	0.07(0.0252-0.096)	0.064(0.0408-0.0894)	0.069(0.0418-0.0944)	0.071(0.047-0.0954)	0.072(0.0506-0.0988)	0.096(0.068-0.1164)	0.046(0.032-0.085)	0.073(0.05355-0.09315)	0.079(0.0566-0.1409)	0.067(0.0622-0.0734)	0.064(0.05875-0.0715)	0.063(0.0591-0.07145)
Luluwa	0.065(0.036-0.089)	0.077(0.053-0.101)	0.069(0.038-0.09515)	0.035(0.023-0.05615)	0.07(0.04-0.09515)	0.041(0.03-0.05915)	0.062(0.03485-0.08815)	0.066(0.04-0.09015)	0.048(0.039-0.06315)	0.103(0.07155-0.1273)	0.073(0.05355-0.09315)	0.035(0.026-0.0603)	0.078(0.04025-0.1519)	0.068(0.0376-0.0812)	0.052(0.029-0.07675)	0.048(0.026-0.06625)
Dekese	0.041(0.031-0.056)	0.073(0.04965-0.13595)	0.057(0.0359-0.11995)	0.073(0.0317-0.14695)	0.059(0.0369-0.12295)	0.074(0.03865-0.14595)	0.05(0.025-0.121)	0.057(0.035-0.12395)	0.066(0.04195-0.13595)	0.063(0.03795-0.08585)	0.079(0.0566-0.1409)	0.078(0.04025-0.1519)	0.058(0.04395-0.1039)	0.05(0.0416-0.0692)	0.054(0.044-0.06825)	0.053(0.04355-0.07525)
Songe	0.038(0.03-0.059)	0.062(0.056-0.0746)	0.036(0.0246-0.0624)	0.062(0.0302-0.0752)	0.038(0.0276-0.0636)	0.064(0.0362-0.0754)	0.04(0.0288-0.0554)	0.047(0.038-0.0604)	0.054(0.039-0.0648)	0.064(0.0556-0.089)	0.067(0.0622-0.0734)	0.068(0.0376-0.0812)	0.05(0.0416-0.0692)	0.029(0.0228-0.0612)	0.036(0.02275-0.058)	0.038(0.0241-0.0677)
Tetela	0.042(0.031-0.076)	0.059(0.05125-0.069)	0.037(0.02375-0.06)	0.047(0.02525-0.071)	0.039(0.02575-0.061)	0.05(0.0325-0.07075)	0.041(0.032-0.052)	0.047(0.039-0.05625)	0.047(0.038-0.05975)	0.074(0.0605-0.09475)	0.064(0.05875-0.0715)	0.052(0.029-0.07675)	0.054(0.044-0.06825)	0.036(0.02275-0.058)	0.034(0.0275-0.04425)	0.034(0.02755-0.0487)
Mbala	0.043(0.038-0.047)	0.061(0.054-0.0759)	0.044(0.0313-0.0687)	0.042(0.0241-0.05915)	0.046(0.03375-0.06915)	0.046(0.0311-0.06025)	0.039(0.02955-0.0598)	0.045(0.03755-0.06335)	0.044(0.0401-0.04935)	0.074(0.05865-0.10225)	0.063(0.0591-0.07145)	0.048(0.026-0.06625)	0.053(0.04355-0.07525)	0.038(0.0241-0.0677)	0.034(0.02755-0.0487)	0.032(0.02455-0.0467)

Table S9: TVD values based on the CHROMOPAINTER coancestry heatmap under the **All-donors** analysis using the *DRC-only* dataset. Only ethnic groups with >10 individuals are included. Results are provided for the mean and 5-95% quantiles across all pairwise comparisons of individuals, under unlinked and linked models that ignore and exploit haplotype information, respectively. Pairwise TVD results for each individual are shown in Figure S4.

TVD Unlinked

Group	Kuba_all	Kuba_Bushong	Kuba_Ngeende	Kuba_Pyang	Kuba_Other	Luluwa	Luntu	Lele	Kete S	Kete N	Bindi	Luba	Dekese	Songe	Tetela	Mbala
Kuba_all						0	1	0.235	0	0	0	0.998	0.726	0.001	0.122	0.133
Kuba_Bushong			0.051	0.051	0.118	0.008	1	0.169	0	0	0	0.99	0.841	0.001	0.402	0.426
Kuba_Ngeende		0.051		0.09	0.359	0.191	1	0.026	0.002	0.004	0.001	1	0.467	0.381	0.361	0.842
Kuba_Pyang		0.051	0.09		0.135	0.098	1	0.043	0.006	0	0.008	0.994	0.572	0.129	0.638	0.753
Kuba_Other		0.118	0.359	0.135		0.195	1	0.028	0.004	0.002	0.068	0.998	0.458	0.527	0.807	0.787
Luluwa	0	0.008	0.191	0.098	0.195		0	0	0	0	0	0.003	0.003	0.001	0.101	0.354
Luntu	1	1	1	1	1	0		1	1	1	0.996	0.998	1	1	1	1
Lele	0.235	0.169	0.026	0.043	0.028	0	1		0	0	0.001	1	0	0.259	0	0
Kete S	0	0	0.002	0.006	0.004	0	1	0		0	0	1	0.009	0.003	0.04	0
Kete N	0	0	0.004	0	0.002	0	1	0	0		0	0.028	0.552	0	0.103	0.201
Bindi	0	0	0.001	0.008	0.068	0	0.996	0.001	0	0		0.025	0.888	0	0.803	0.588
Luba	0.998	0.99	1	0.994	0.998	0.003	0.998	1	1	0.028	0.025		1	0.839	1	1
Dekese	0.726	0.841	0.467	0.572	0.458	0.003	1	0	0.009	0.552	0.888	1		0.996	0.081	0.321
Songe	0.001	0.001	0.381	0.129	0.527	0.001	1	0.259	0.003	0	0	0.839	0.996		0.904	0.821
Tetela	0.122	0.402	0.361	0.638	0.807	0.101	1	0	0.04	0.103	0.803	1	0.081	0.904		0.371
Mbala	0.133	0.426	0.842	0.753	0.354	0.354	1	0	0	0.201	0.588	1	0.321	0.821	0.371	

TVD Linked

Group	Kuba_all	Kuba_Bushong	Kuba_Ngeende	Kuba_Pyang	Kuba_Other	Luluwa	Luntu	Lele	Kete S	Kete N	Bindi	Luba	Dekese	Songe	Tetela	Mbala
Kuba_all						1	1	1	1	1	1	1	1	0.997	0.997	0.998
Kuba_Bushong			1	1	1	1	1	1	1	1	1	1	1	0.997	0.997	0.998
Kuba_Ngeende		1		0.994	0.995	1	1	1	1	1	1	1	1	0.995	0.996	0.994
Kuba_Pyang		1	0.994		0.998	1	1	1	1	1	1	1	1	0.996	0.996	0.994
Kuba_Other		1	0.995	0.998		1	1	1	1	1	1	1	1	0.997	0.997	0.998
Luluwa	1	1	1	1	1		1	1	1	1	1	1	1	1	1	1
Luntu	1	1	1	1	1	1		1	1	1	1	1	1	1	1	1
Lele	1	1	1	1	1	1	1		1	1	1	1	1	1	1	1
Kete S	1	1	1	1	1	1	1	1		1	1	1	1	1	1	0.995
Kete N	1	1	1	1	1	1	1	1	1		1	1	1	1	1	1
Bindi	1	1	1	1	1	1	1	1	1	1		1	1	1	1	1
Luba	1	1	1	1	1	1	1	1	1	1	1		1	0.996	1	1
Dekese	1	1	1	1	1	1	1	1	1	1	1	1		1	1	1
Songe	0.997	0.997	0.995	0.996	0.997	1	1	1	1	1	1	0.996	1		0.998	1
Tetela	0.997	0.997	0.996	0.996	0.997	1	1	1	1	1	1	1	1	0.998		1
Mbala	0.998	0.997	0.994	0.994	0.998	1	1	1	0.995	1	1	1	1	1	1	

Table S10: The proportion of times, based on 1000 permutations, that the true mean TVD across all pairwise combinations of individuals from the given two DRC groups is greater than that after randomly permuting labels within the two groups. Results are provided for the **All-donors** analysis applied to the *DRC-only* dataset using unlinked (top) and linked (bottom) CHROMOPAINTER models.

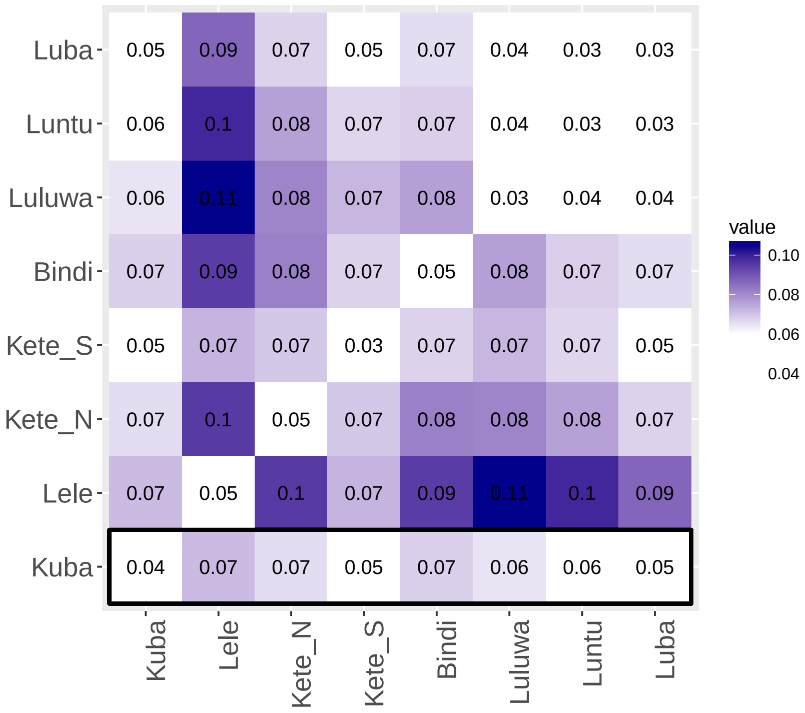

Figure S5: Heatmap providing the pairwise TVD between ethnic groups with >30 individuals under the **All-donors** linked anaylsis using the *DRC-only* dataset. Values provide the mean TVD across all pairing of individuals between the two groups, also reflected in the color scheme and scale at right. Estimates with 5-95% CIs are also provided in Table S9 with the distribution of TVD within groups provided in Figure S6.

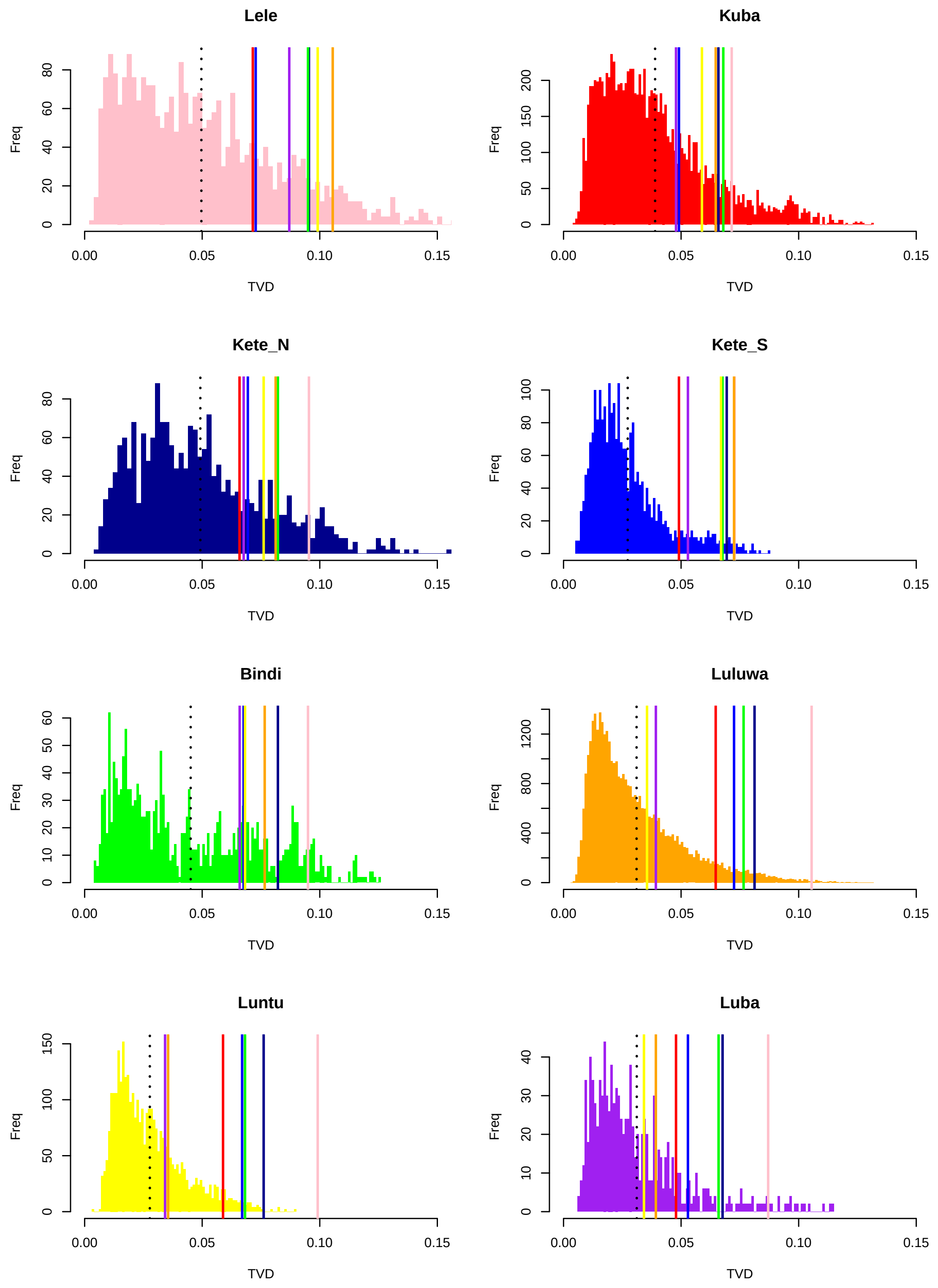

Figure S6: Histograms of pairwise TVD between all individuals within each ethnic group with >30 individuals under the **All-donors** analysis using the *DRC-only* dataset, with the black dotted vertical line giving the mean TVD. Colors correspond to different ethnic groups as defined in main text Figure 1 and given in the header of each plot, with colored horizontal bars giving the mean TVD across individual pairings between that group and every other depicted ethnic label.

S6 Clustering individuals within the DRC based on haplotype sharing

Haplotype-based clustering was performed using fineSTRUCTURE (Lawson et al., 2012) in order to group DRC individuals into genetically homogeneous clusters based on the CHROMOPAINTER inferred painting profiles. This approach is advantageous as it allows individuals to be grouped, independent of self-reported identity, into cohorts based entirely on their genetics. A clustering based approach is also useful in this case where there are several ethnic groups with very few individuals (for example the Kusu, Mongo and Moyisambo in this dataset) which can potentially be clustered together, increasing analytical power.

fineSTRUCTURE was applied to the *DRC-only* dataset based on the inferred "*.chunkcounts.out*" painting profiles under the **All-donors** analysis, estimating the normalization parameter ($c = 0.62$) as recommended by the authors. fineSTRUCTURE was run sampling clustering assignments every 10,000 iterations for 1×10^6 MCMC iterations after 1×10^6 burn-in steps. We then performed an additional 1×10^5 hill-climbing iterations, starting from the MCMC sample with highest posterior probability. This step resulted in an initial classification of individuals into 28 clusters.

In order to assess the confidence associated with the assignment of any single DRC individual to each cluster, we applied an additional procedure to the fineSTRUCTURE output which (potentially) reassigns individuals to clusters based on which individuals they are grouped with at each MCMC sample (as described in Leslie et al. (2015)). Briefly, for each individual a, this procedure calculates the proportion of individuals in the cluster to which a is assigned in a given MCMC iteration that are comprised of individuals from each fineSTRUCTURE-inferred cluster. It then sums these proportions across MCMC iterations and assigns a to the fineSTRUCTURE-inferred cluster with the highest such sum. This potentially re-classifies individuals to different clusters; these clusters are then used as the new "fineSTRUCTURE-inferred clusters" and the above procedure is run again. As in Leslie et al. (2015), we used 50 iterations of this process, which assigned individuals to 23 final clusters. This procedure also calculates the confidence of each individual a's final cluster assignment, using the relative magnitude of these sums. For each individual, we assessed the confidence of their cluster membership, as well as the correlation between ethnic group label and cluster across individuals. We also used fineSTRUCTURE to build a simple bifurcating tree relating these 23 clusters, merging clusters pair-at-time using the greedy approach of Lawson et al. (2012) that identifies – at each step – the merge that least decreases the posterior probability of the fineSTRUCTURE model. Table S11 and Figure S7 provide this final cluster assignment for the *DRC-only* merge.

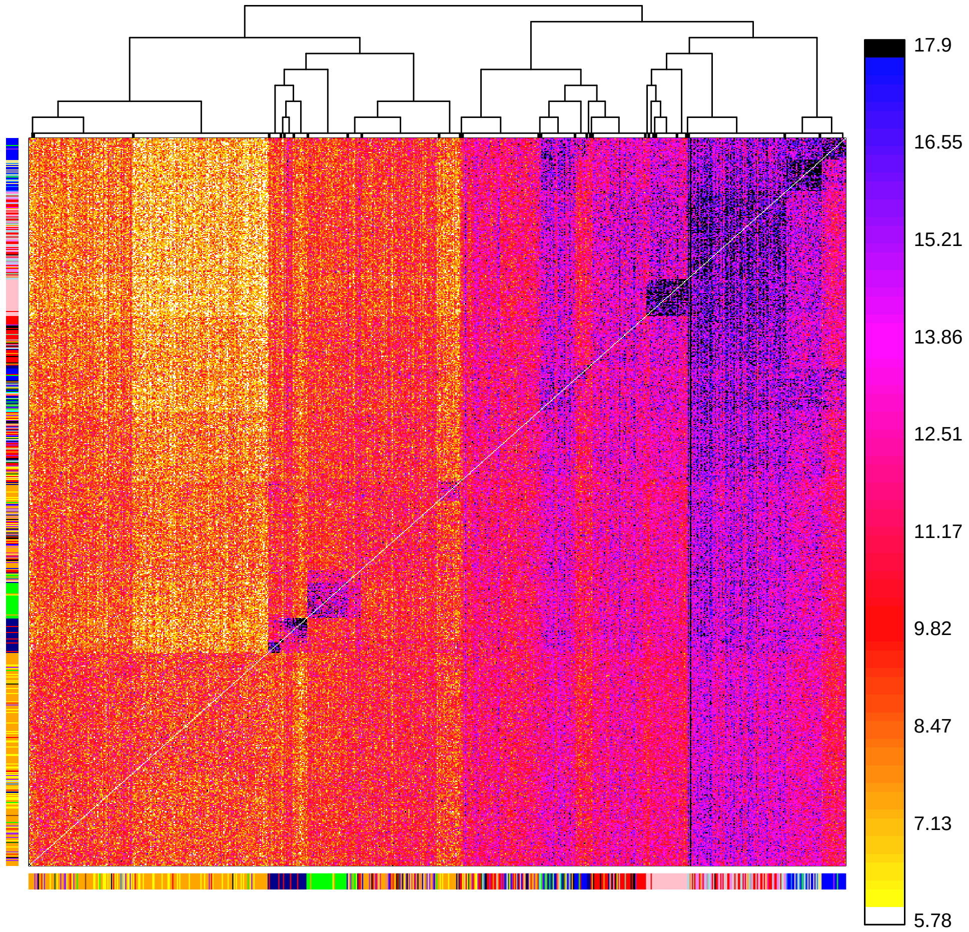

Figure S7: CHROMOPAINTER's inferred number of matching segments of genome-wide DNA that each individual (column) copies from each other individual (row) in the *DRC-only* dataset, ordered by our final inferred 23 clusters. The tree at the top shows fineSTRUCTURE's inferred hierarachical merging of these 23 clusters. Axis colors denote the ethnic group to which each individual belongs, using the color scheme from Figure 1 in the main text. The final inferred clustering is provided in Table S11.

Cluster	n.inds	Group.Assignment	Kuba	TVD_all	TVD_same	TVD_diff
**1**	2	Lele(2)	0	0.041(0.041-0.041)		
**2**	19	Lele(18)Kuba(1)	1	0.056(0.045-0.07)	0.057(0.046-0.073)	0.047(0.047-0.047)
**3**	11	Lele(11)	0	0.056(0.045-0.072)		
**4**	3	Lele(3)	0	0.058(0.048-0.064)		
**5**	2	Tetela(2)	0	0.034(0.034-0.034)		
**6**	85	Kuba(30)Dekese(19)Lele(12)Tetela(10)Kongo(4)Luluwa(3)Pende(3)Dinga(1)Kusu(1)Lubakat(1)Songe(1)	30	0.058(0.048-0.079)	0.041(0.025-0.061)	0.057(0.033-0.087)
**7**	69	Kuba(23)Luluwa(10)Luba(7)Luntu(6)Kete_N(5)Lele(3)Tshokwe(3)Kete_S(2)Nyoka(2)Songe(2)Tetela(2)Bunde(1)Kongo(1)Mbala(1)Moyisambo(1)	22	0.058(0.047-0.081)	0.052(0.035-0.075)	0.057(0.038-0.083)
**8**	3	Kete_N(3)	0	0.047(0.044-0.051)		
**9**	45	Kuba(27)Songe(7)Kete_N(2)Luluwa(2)Dekese(1)Luba(1)Lunda(1)Luntu(1)Nyoka(1)Pende(1)Tetela(1)	27	0.061(0.047-0.099)	0.051(0.035-0.092)	0.067(0.043-0.124)
**10**	9	Kete_S(7)Bindi(1)Kuba(1)	1	0.055(0.044-0.073)	0.047(0.039-0.063)	0.067(0.061-0.073)
**11**	20	Kete_S(18)Bindi(1)Luba(1)	0	0.047(0.041-0.057)	0.046(0.041-0.054)	0.054(0.04-0.069)
**12**	29	Kete_S(12)Mbala(10)Sala(7)	0	0.042(0.035-0.061)	0.04(0.025-0.058)	0.043(0.03-0.064)
**13**	27	Kete_S(8)Bindi(4)Tshokwe(4)Luluwa(3)Bemba(1)Lele(1)Luba(1)Lunda(1)Nyambi(1)Nyoka(1)Pende(1)Songe(1)	0	0.045(0.04-0.054)	0.039(0.025-0.048)	0.047(0.035-0.066)
**14**	2	Luluwa(2)	0	0.041(0.041-0.041)		
**15**	77	Luluwa(44)Luntu(16)Luba(7)Bindi(2)Kuba(2)Mfuya(1)Mongo(1)Sala(1)Songe(1)Tetela(1)Tshokwe(1)	2	0.055(0.046-0.068)	0.047(0.038-0.06)	0.057(0.036-0.083)
**16**	122	Luluwa(87)Luntu(26)Kuba(4)Luba(3)Bindi(1)Songe(1)	4	0.052(0.043-0.069)	0.047(0.038-0.062)	0.056(0.036-0.079)
**17**	15	Kete_N(13)Kuba(2)	2	0.08(0.065-0.111)	0.074(0.04-0.104)	0.081(0.044-0.138)
**18**	12	Kete_N(11)Kuba(1)	1	0.055(0.047-0.075)	0.057(0.049-0.076)	0.047(0.047-0.047)
**19**	9	Bindi(3)Kuba(2)Luluwa(2)Lele(1)Luntu(1)	2	0.065(0.055-0.083)	0.061(0.034-0.076)	0.068(0.042-0.095)
**20**	34	Bindi(31)Luluwa(2)Luntu(1)	0	0.053(0.044-0.064)	0.052(0.043-0.063)	0.058(0.031-0.09)
**21**	71	Luluwa(32)Luba(13)Kuba(6)Luntu(4)Songe(4)Kete_S(3)Mfuya(2)Nyambi(2)Bena_Konji(1)Bindi(1)Lele(1)Mbala(1)Tshokwe(1)	6	0.056(0.046-0.067)	0.05(0.039-0.066)	0.055(0.037-0.073)
**22**	11	Kete_N(9)Kuba(2)	2	0.042(0.035-0.058)	0.043(0.032-0.066)	0.049(0.039-0.062)
**23**	16	Luluwa(11)Luntu(3)Kete_N(2)	0	0.054(0.046-0.065)	0.045(0.038-0.054)	0.062(0.042-0.079)

Table S11: Final cluster assignment into 23 genetically homogeneous groups we label 1-23. Each cluster contains "n.inds" total number of individuals, with the number from each ethnic group given in "Group.Assignment". The total number of individuals in each cluster that identify as belonging to the former Kuba Kingdom (Kuba) are provided together with the mean and 95% empirical distribution of pairwise TVD scores between all individuals within the cluster (TVD_all), between only individuals of the same ethnic group within the cluster (TVD_same), and between only individuals belonging to different ethnic groups within the cluster (TVD_diff), all based on the *DRC-only* dataset.

Many of the inferred clusters contain small numbers of individuals from multiple ethnic groups, suggesting a weak relationship between group label and genetic cluster and/or that sub-structure related to ethnic label is not well-captured by fineSTRUCTURE. Some of our results point towards the latter being the case. For example, often the average TVD between individuals of the same group label within a cluster is lower than that between individuals of different group labels within the same cluster (Figure S8, Table S11). As a result, in our subsequent analyses we grouped individuals by group label rather than inferred cluster, with the exception of – in some analyses as noted – splitting our 95 Kete individuals into two fineSTRUCTURE-inferred clusters which we term "Kete_N" (45 individuals) and "Kete_S" (50 individuals), which are separated by relatively large genetic (Figure S7, Table S11) and geographic (main text Figure 1, Table S2) distances.

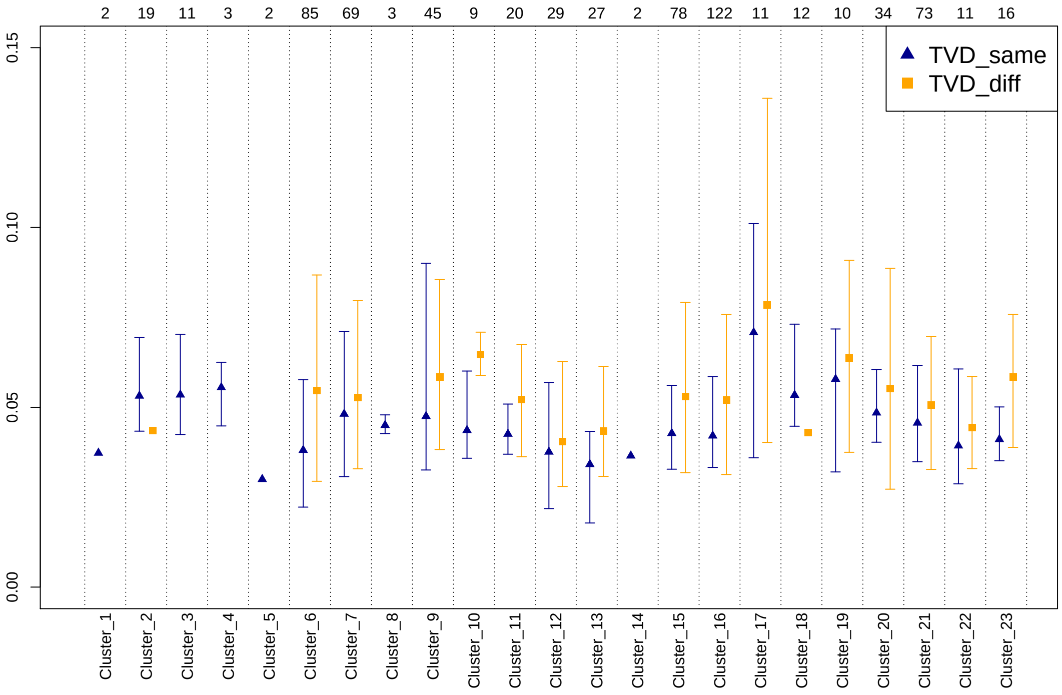

Figure S8: Mean TVD, along with 5-95% empirical values, across pairwise combinations of individuals within each inferred fineSTRUCTURE cluster, when considering only individuals belonging to the same ethnic group (blue triangle) or only individuals belonging to different ethnic groups (orange square). The sample size of each cluster is given at the top axis.

Of note, no cluster is comprised of entirely Kuba individuals. To evaluate the extent to which individuals from each ethnic group are scattered across different clusters, we used group label information to calculate the per group entropy statistic. To calculate entropy, for each ethnic group j we calculate p_{ij}, the probability that an individual of ethnic group j belongs to cluster i, as: $p_{ij} = m_{ij}/m_j$, where m_j is the number of individuals in ethnic group j and m_{ij} is the number of clusters to which individuals in group j are assigned. The entropy of each group is then calculated using the standard formula (Schütze et al., 2008): $e_j = \sum_{i=1}^{L} p_{ij} \times log(p_{ij})$, where L is the total number of fineSTRUCTURE clusters, in this case 23 (Figure S9).

The Kuba (assigned to ten possible clusters) have the highest entropy in fineSTRUCTURE cluster assignment except for that inferred in the combined Kete, which show significant geographic clustering (Table S11, Figure S7, main text Figure 1). This is consistent with a lack of genetic isolation in the Kuba relative to many neighbouring ethnic groups.

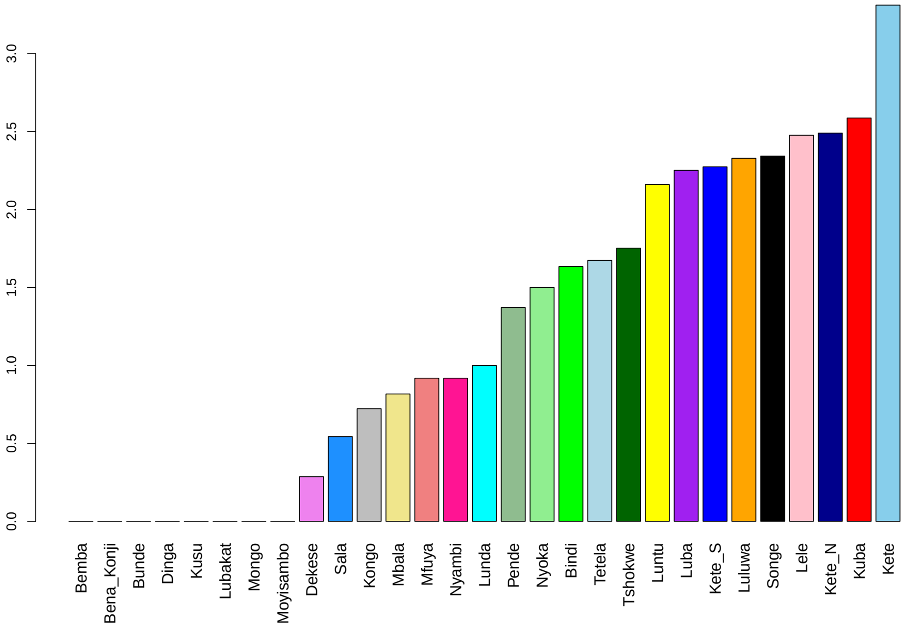

(a) Group Entropy

Figure S9: Barplots providing the per group entropy based on comparison of group label to fineSTRUCTURE cluster. Bars are colored according to ethnic group label as in Figure 1 of the main text. The Kete are shown collectively and split into the Northern and Southern groupings (Kete_N and Kete_S).

S7 Inferring ratios of most recent ancestor sharing between groups

To further explore the extent to which individuals from different groups are related ancestrally, we used CHROMOPAINTER (Lawson et al., 2012) to match haplotype patterns in each DRC individual's genome to a donor set consisting of an equal number of individuals from their own group and another DRC group. In this way, we pairwise compared all DRC ethnic groups that contained >30 individuals: Kuba, Luluwa, Luntu, Bindi, Kete (combined, plus split based on geography into Northern (Kete_N) and Southern (Kete_S) groups), Lele, and Luba in the *DRC-only* dataset. The group with the next largest sample size was the Dekese, which only has 20 individuals, and we show in this section that $\leq$ 33 individuals may not be sufficient data to accurately capture group diversity in this dataset. As described in **Methods**, when comparing individuals from group A containing n_A individuals to those in group B containing n_B individuals, we first randomly sampled $n + 1$ individuals from group A and n individuals from group B, where $n = \min(n_A - 1, n_B)$ is the number of individuals in group X. We then applied CHROMOPAINTER with default settings to paint each of the $n+1$ individuals from A using the other $2n$ sampled individuals from A and B as donors, dividing the total proportion of genome-wide DNA each individual matches to A by the amount they match to B. We matched for sample size in these comparisons, because we want to compare the relative amount of matching to each group under CHROMOPAINTER, which is sensitive to sample size. This painting procedure is schematized in the top of main text Figure 2b and was repeated for all pairwise combinations of these DRC groups, with results provided in Figure S10 and Table S12.

As expected, on average individuals typically matched a higher proportion of haplotype segments to members of their own ethnic group relative to those from the other group. However, notably Kuba and Luba have amongst the smallest ratios of average proportional haplotype matching to their own group relative to other groups (see Kuba and Luba columns in Figure S10). This observation is consistent with results from previous sections for the Kuba ethnic group, but does not match previous observations in the Luba (e.g. Figures S6, S9). Furthermore, notably other DRC groups consistently match a relatively high proportion of haplotypes with members of their own group relative to Kuba (see Kuba row in Figure S10), indicative of isolation effects in these groups, which is not the case in the Luba. Given Luba are the group with the lowest sample size included in this analysis (33 individuals), we explored whether their signal was being driven by the smaller sample size of the comparison. To do so, we randomly reduced the sample size of Kuba to 33 individuals to match the sample size of Luba (Kuba_33 in Figure S10 and Table S12). Reducing the sample size of Kuba resulted in a similar pattern to that observed in Luba (see columns/rows of Kuba_33 and Luba, relative to Kuba, in Figure S10). This suggests a notable lack of power to identify these relationships in comparisons with a sample size >33. For this reason, we repeated this analysis using only groups with >40 individuals, which removed Luba from the seven labelled groups listed above, while also matching for sample size across all groups (Figure 2b of main text).

We also explore whether the pattern observed in the full Kuba is also seen in Bushong (n=47), the only sub-group of Kuba with a sample size >33. Indeed Bushong show a similar relationship as Kuba (see columns versus rows of Bushong in Figure S10, relative to that of Kuba and other groups). Sub-sampling Bushong to 33 individuals also resulted in a similiar reduction in the assymetric trend (high ratios in the row, low ratios in the column) that was seen in Kuba_33. Notably, the simulated populations AdmixPop and MixPop (described in Section S8 and in main text **Methods**) showed similar trends to that observed in the full Kuba.

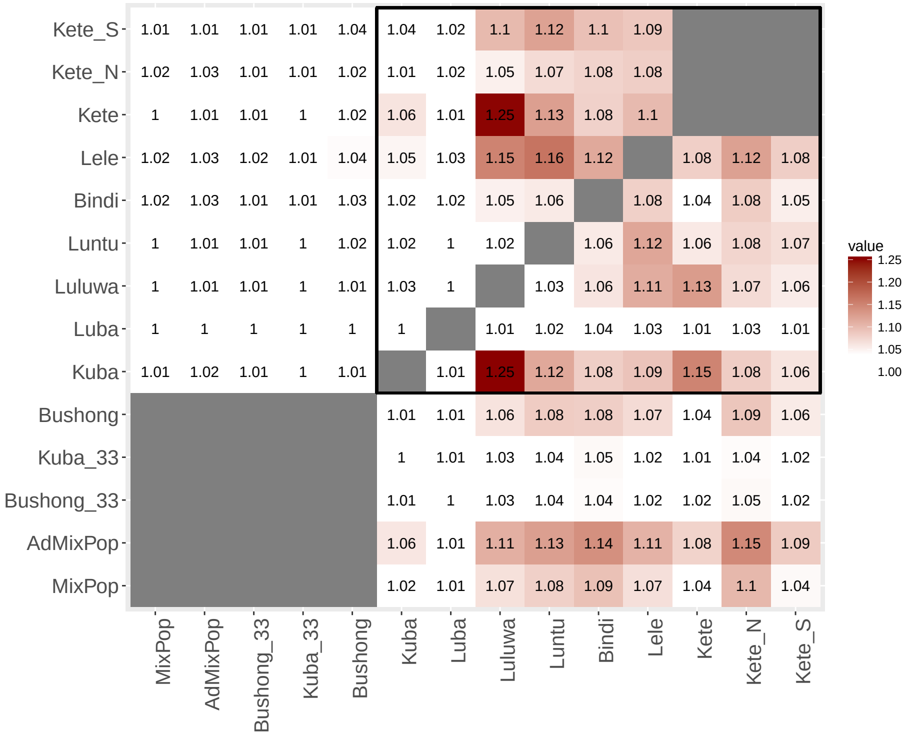

Figure S10: Factor increase by which members of given group (column) share most recent ancestors with other individuals from their own group relative to individuals from a different group (row), when matching only to individuals from those two groups using CHROMOPAINTER. Equivalent comparisons are given for Bushong (a sub-group of the Kuba), random sub-samples to 33 Kuba (Kuba_33) and 33 Bushong (Bushong_33) (matching Luba, which has the smallest sample size of all groups depicted), and the simulated groups "MixPop" and "AdMixPop" (see Section S8 and main text **Methods**). For each comparison the mean and 5%-95% empirical quantiles across individuals are provided in Table S12. Those comparisons shown in grey were not performed, e.g. due to the use of overlapping individuals in the groupings being compared.

Group	MixPop	AdMixPop	Bushong_33	Kuba_33	Bushong	Kuba	Luba	Luluwa	Luntu	Bindi	Lele	Kete(Combined)	Kete_N	Kete_S
MixPop						1.005(0.985-1.028)	1.002(0.992-1.009)	1.002(0.985-1.019)	1.004(0.985-1.026)	1.016(0.986-1.044)	1.023(0.992-1.048)	1.003(0.988-1.023)	1.017(0.989-1.046)	1.006(0.986-1.027)
AdMixPop						1.016(0.933-1.131)	1.002(0.99-1.013)	1.01(0.95-1.124)	1.013(0.975-1.118)	1.027(0.996-1.042)	1.034(0.99-1.185)	1.011(10.996-1.048)	1.027(0.985-1.050)	1.014(0.988-1.030)
Bushong_33						1.008(0.991-1.034)	1.005(0.99-1.028)	1.006(0.995-1.028)	1.007(0.995-1.033)	1.015(0.993-1.043)	1.02(1.002-1.038)	1.009(0.994-1.031)	1.014(0.996-1.042)	1.015(0.999-1.036)
Kuba_33						0.998(0.984-1.009)	1.002(0.991-1.012)	1.005(0.994-1.011)	1.005(0.992-1.015)	1.013(0.996-1.025)	1.013(0.994-1.026)	1.005(0.992-1.018)	1.009(0.996-1.026)	1.011(0.998-1.025)
Bushong						1.014(0.983-1.045)	1.005(0.99-1.028)	1.011(0.988-1.046)	1.019(0.992-1.049)	1.026(0.99-1.048)	1.043(1.005-1.077)	1.024(0.994-1.067)	1.025(0.988-1.052)	1.036(1.003-1.062)
Kuba	1.019(0.992-1.043)	1.061(1.033-1.093)	1.006(0.994-1.02)	1(0.983-1.023)	1.01(0.982-1.04)		1.002(0.991-1.012)	1.033(0.96-1.145)	1.019(0.988-1.064)	1.019(0.994-1.04)	1.049(1.007-1.09)	1.064(0.958-1.157)	1.011(0.988-1.036)	1.037(1.002-1.084)
Luba	1.011(0.996-1.023)	1.011(0.996-1.023)	1.004(1-1.009)	1.015(0.998-1.028)	1.013(0.997-1.03)	1.015(0.998-1.028)		0.999(0.987-1.014)	0.997(0.984-1.01)	1.018(1.001-1.03)	1.032(1.006-1.05)	1.011(0.992-1.026)	1.016(1-1.034)	1.019(0.992-1.037)
Luluwa	1.067(0.993-1.114)	1.111(1.058-1.149)	1.028(0.985-1.054)	1.03(0.987-1.057)	1.065(0.974-1.128)	1.25(0.98-1.534)	1.013(0.989-1.033)		1.015(0.946-1.095)	1.053(0.976-1.097)	1.151(1.047-1.228)	1.249(1.01-1.447)	1.053(0.994-1.093)	1.101(1.019-1.158)
Luntu	1.081(0.999-1.136)	1.126(1.076-1.162)	1.036(1.007-1.056)	1.036(1.009-1.062)	1.085(1.027-1.129)	1.117(1.018-1.215)	1.016(0.991-1.042)	1.025(0.944-1.086)		1.059(1.009-1.102)	1.164(1.07-1.229)	1.125(1.026-1.216)	1.072(1.018-1.106)	1.123(1.035-1.171)
Bindi	1.095(1.01-1.161)	1.139(1.033-1.214)	1.044(0.993-1.082)	1.046(0.994-1.084)	1.083(0.988-1.147)	1.083(0.989-1.156)	1.038(0.994-1.071)	1.063(0.978-1.123)	1.059(0.972-1.122)		1.117(1.032-1.183)	1.08(0.993-1.138)	1.078(0.993-1.142)	1.095(1-1.152)
Lele	1.068(1.007-1.13)	1.108(1.034-1.178)	1.024(1-1.075)	1.021(0.996-1.063)	1.071(1.001-1.149)	1.092(1.003-1.204)	1.028(1.004-1.068)	1.114(1.014-1.23)	1.119(1.016-1.233)	1.081(1.02-1.151)		1.101(1.009-1.206)	1.083(1.013-1.15)	1.089(1.013-1.179)
Kete(Combined)	1.038(1.003-1.097)	1.079(1.043-1.138)	1.015(0.997-1.035)	1.014(0.997-1.028)	1.04(0.997-1.095)	1.15(0.989-1.402)	1.008(0.994-1.024)	1.128(0.983-1.338)	1.059(1.004-1.174)	1.037(0.995-1.091)	1.082(1.033-1.165)			
Kete_N	1.103(1.032-1.192)	1.145(1.064-1.229)	1.046(0.997-1.087)	1.044(1.005-1.084)	1.09(0.997-1.169)	1.084(0.998-1.176)	1.034(1.002-1.075)	1.071(1.004-1.152)	1.077(1.001-1.162)	1.084(1.013-1.153)	1.124(1.02-1.218)			
Kete_S	1.045(1.001-1.089)	1.086(1.042-1.144)	1.022(1-1.036)	1.021(1.005-1.038)	1.058(1.007-1.095)	1.064(1.014-1.115)	1.015(0.996-1.03)	1.057(1.007-1.099)	1.069(1.015-1.115)	1.055(1-1.096)	1.083(1.032-1.133)			

Table S12: Factor increase by which members of given group (row) share most recent ancestors with other individuals from their own group relative to individuals from a different group (column), when matching only to individuals from those two groups using CHROMOPAINTER (as depicted in Figure S10). Equivalent comparisons are given for the Bushong (a sub-group of the Kuba), random sub-samples to 33 Kuba and 33 Bushong (matching the sample size of the Luba), and the simulated groups “MixPop” and “AdMixPop” (see Section S8). For each comparison the mean and 5%-95% empirical quantiles across individuals are provided.

As noted above, we also assessed the effect of varying sample sizes across groups by repeating this analysis when fixing each group's sample size to match that of the Bindi (44 individuals), which is the next smallest group after excluding the Luba. These results are provided in main text Figure 2b and Tables S13-S14. Consistent with previous results, on average individuals matched a higher proportion of haplotype segments to members of their own ethnic group relative to those from the other group. However, again consistent with previous results, this ratio was smallest in Kuba and Bushong, a Kuba sub-group. Note, this pattern was also observed in the simulated popuations MixPop and AdmixPop, as described in Section S8 and in the main text **Methods**, meant to mimic the formation of the Kuba Kingdom.

We next sought to evaluate whether the ratios in the Kuba were significantly lower than that of other ethnicities under this sample-size matched analysis. To do so, for each ethnicity B, we used a two-sample t-test to assess whether each ethnic groups mean ratio of recent ancestry matching to individuals from their own ethnicity versus individuals from B was less that that of other ethnic groups. Table S14 shows the number of such pairwise comparisons for which the two-sample t-test gave a one-sided p-value <0.01. For each group B, the Kuba had the most comparisons matching this criterion, suggesting that the ratios observed in the Kuba are significantly lower than those in other ethnicities. However, as is the case with the CHROMOPAINTER analyses above, these t-test results are for comparison only, as significance is challenging to assess here given the inferred paintings for individuals include overlapping donors and hence are not independent, and permutations are impractical given the computational expense of this analysis. Nonetheless, overall these results highlight how non-Kuba DRC groups in our sample have a higher degree of genetic isolation than our sampled Kuba individuals, and – conversely – that Kuba have a higher degree of genetic similarity to neighbouring DRC groups.

Group	MixPop	AdMixPop	Bushong	Kuba	Luluwa	Luntu	Bindi	Kete_N	Kete_S	Kete	Lele
MixPop			1.006(0.978-1.024)	1.005(0.985-1.028)	1.002(0.985-1.019)	1.004(0.985-1.026)	1.016(0.986-1.044)	1.017(0.989-1.046)	1.006(0.986-1.027)	1.003(0.988-1.023)	1.023(0.992-1.048)
AdMixPop			1.027(0.944-1.108)	1.026(0.943-1.141)	1.02(0.958-1.034)	1.023(0.979-1.038)	1.037(0.952-1.059)	1.037(0.994-1.054)	1.029(1.016-1.085)	1.024(0.987-1.039)	1.044(0.955-1.069)
Bushong	1.026(0.988-1.056)	1.01(1.005-1.034)		1.012(0.986-1.043)	1.008(0.989-1.036)	1.015(0.989-1.04)	1.026(0.99-1.048)	1.023(0.986-1.048)	1.031(1.002-1.049)	1.021(0.996-1.055)	1.038(1.006-1.067)
Kuba	1.019(0.992-1.043)	1.051(1.023-1.073)	1.008(0.985-1.033)		1.003(0.983-1.02)	1.007(0.99-1.025)	1.019(0.994-1.04)	1.011(0.989-1.037)	1.023(0.999-1.045)	1.011(0.985-1.035)	1.029(1.001-1.058)
Luluwa	1.067(0.993-1.114)	1.005(0.998-1.111)	1.054(0.974-1.106)	1.054(0.99-1.106)		1.006(0.971-1.043)	1.053(0.976-1.097)	1.053(0.993-1.091)	1.076(1.011-1.12)	1.057(0.991-1.104)	1.1(1.018-1.15)
Luntu	1.081(0.999-1.136)	1.067(1.036-1.102)	1.069(1.021-1.105)	1.068(1.02-1.119)	1.016(0.981-1.048)		1.059(1.009-1.102)	1.067(1.016-1.103)	1.094(1.038-1.128)	1.071(1.023-1.119)	1.116(1.056-1.157)
Bindi	1.095(1.01-1.161)	1.083(1.033-1.201)	1.083(0.988-1.147)	1.083(0.989-1.156)	1.063(0.978-1.123)	1.059(0.972-1.122)		1.078(0.993-1.142)	1.095(1-1.152)	1.08(0.993-1.138)	1.117(1.032-1.183)
Kete_N	1.103(1.032-1.192)	1.086(1.042-1.144)	1.087(0.996-1.163)	1.08(0.998-1.168)	1.07(1.004-1.149)	1.075(1-1.155)	1.084(1.013-1.153)				1.119(1.01-1.209)
Kete_S	1.045(1.001-1.089)	1.034(1.014-1.099)	1.05(1.002-1.083)	1.048(1.01-1.088)	1.047(1.005-1.076)	1.056(1.015-1.097)	1.055(1-1.096)				1.066(1.03-1.104)
Kete	1.038(1.003-1.097)	1.024(1.014-1.119)	1.035(0.998-1.08)	1.03(0.988-1.085)	1.027(0.994-1.074)	1.032(0.998-1.087)	1.037(0.995-1.091)				1.057(1.018-1.111)
Lele	1.068(1.007-1.13)	1.054(1.014-1.138)	1.062(1-1.125)	1.058(0.997-1.118)	1.075(1.011-1.148)	1.08(1.014-1.146)	1.081(1.02-1.151)	1.078(1.012-1.139)	1.071(1.012-1.136)	1.066(1.009-1.131)	

Table S13: Factor increase by which members of given group (row) share most recent ancestors with other individuals from their own group relative to individuals from a different group (column), when matching only to individuals from those two groups using CHROMOPAINTER and using only 44 individuals per group (as depicted in main text Figure 2b). Equivalent comparisons are given for Bushong (a sub-group of Kuba), and the simulated groups “MixPop” and “AdMixPop” (see Section S8). For each comparison, the mean and 5%-95% empirical quantiles across individuals are provided.

Group	Kuba	Luluwa	Luntu	Bindi	Kete(Combined)	Lele
Lele	4	0	0	0	3	
Kete(Combined)	4	1	0	0		0
Bindi	4	1	1		2	0
Luntu	3	3		0	2	0
Luluwa	4		2	0	2	0
Kuba		1	0	0	4	0

Table S14: For each group B in the rows, this table shows the number of tests (out of 4) for which the mean ratio given in main text Figure 2b and Table S13 for a given ethnicity (column) was significantly less (one-sided p-value < 0.01 under a two-sample t-test) than that of the 4 other other ethnicities (excluding B) in the same row. Note for all rows but Luntu, the Kuba ratio is significantly less than all other ethnicities. For the Luntu row, the Kuba ratio is significantly less than all ethnicities besides Luluwa.

S8 Simulation framework

As discussed in Section S1.2, it is thought that the formation of the Kuba Kingdom involved the unification of many different groups that resided in the region (Vansina, 1978, p.5). Oral accounts, together with our inferred genetic patterns, suggest that a possible explanation for the genetic trends observed in Kuba individuals is that they descend from genetically different sub-groups that merged in the past during state centralization. To explore this we simulated two artificially mixed populations, MixPop and AdmixPop, as a composition of six DRC groups (see main text **Methods**).

- **MixPop** - Randomly sampled 44 individuals, to match the size of the Bindi, from each of six DRC groups which contained between 8-20 individuals: Dekese, Songe, Tetela, Mbala, Tshokwe, Sala (see Table S1). The sampled individuals were then treated as if one population in subsequent analyses. Thus, MixPop is designed to represent a situation where populations with a similar level of genetic diversity to those present in the region today were unified with no subsequent mixture.

- **AdmixPop** - 44 simulated individuals are composed of haplotype segments matched to those in individuals randomly sampled from each of the Dekese, Songe, Tetela, Mbala, Tshokwe, Sala, in the proportions 25%, 20%, 17%, 17%, 15%, 6% respectively. Segment sizes (in Morgans) were sampled from an exponential distribution with rate ten, chosen to approximately match the time in generations between the Kuba Kingdom's formation and today, as described in Price et al. (2008). AdmixPop is designed to represent a situation where populations with a similar level of genetic diversity to those present in the region today intermixed (i.e. admixed) with each other ten generations ago, corresponding to the start of the Kingdom, and then individuals from this admixed population randomly mated for the duration of the Kingdom's existence prior to Belgian colonisation.

S8.1 Within-group genetic diversity in simulated populations

We explored the within-group homogeneity inferred using fastIBD and CHROMOPAINTER, as described in Sections S4.1 and S4.2, for simulated populations MixPop and AdMixPop.

The simulated groups exhibit patterns of genetic diversity similar to that seen when Kuba is compared to neighbouring groups (main text Figure 2A, Table S6, Figure S11). The true diversity of the Kuba sits somewhere between the patterns of genetic diversity observed in MixPop and AdMixpop. This suggests that the genetic diversity observed in the Kuba today can be explained by the union of genetically distinct groups with subsequent intermixing to some extent in the past.

Furthermore, each of MixPop and AdMixpop showed similar patterns to the real data Kuba when calculating the ratio of haplotype segments for which each group shares most recent ancestry with members of their own group versus each other DRC ethnic group (see Section S7), as illustrated in Figure 2b of the main text and in Figure S10 and Tables S12-S13.

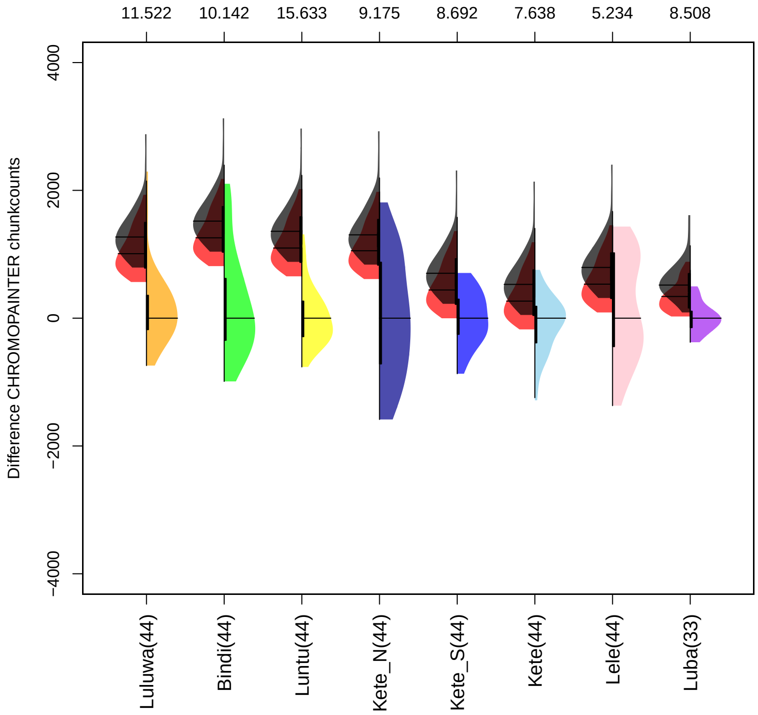

(a) MixPop

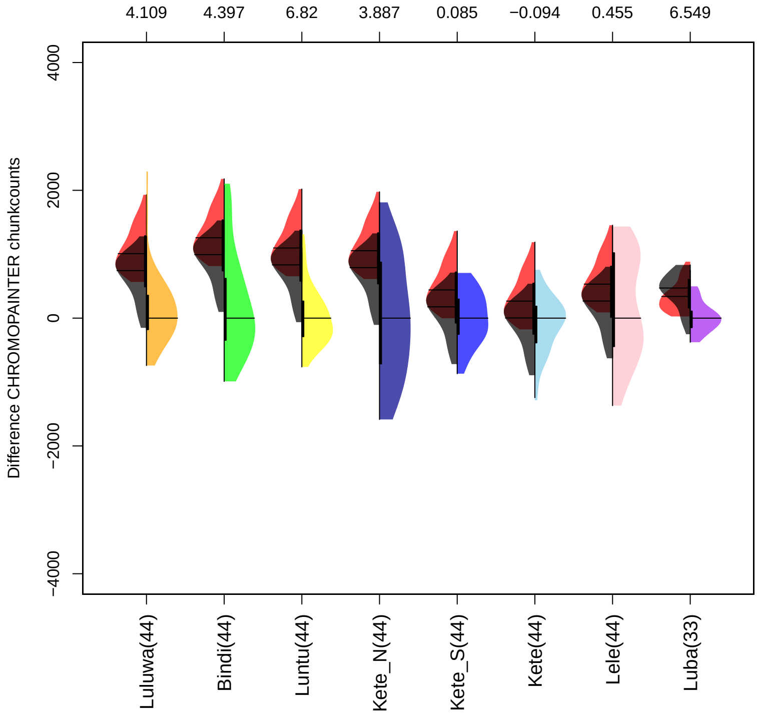

(b) AdMixpop

Figure S11: CHROMOPAINTER inferred number of DNA segments that individuals share with other individuals from their group, which is inversely proportional to DNA segment size, for Kuba (in red) compared to other DRC groups together with the simulated populations (a) MixPop (overlayed on the Kuba in grey) and (b) AdMixPop (overlayed on the Kuba in grey). Two sample t-test statistics (MixPop/AdMixPop - X) are provided at top. Horizontal lines provide the median estimates.

S9 Inferring and dating admixture within the DRC

In order to investigate the admixture history of groups in the DRC, we used GLOBETROTTER (Hellenthal et al., 2014) to describe admixing source groups and date admixture events, using each DRC ethnicity as a putatively admixed (target) group and all other DRC and non-DRC groups as surrogates to the (unknown) admixing sources, using the *DRC-all-world* dataset (described in Section S10). In brief, GLOBETROTTER uses patterns of decay in linkage disequilibrium among haplotype segments assigned to different surrogate pairs (as inferred by CHROMOPAINTER) in order to identify and date any putative DNA admixture events that have occurred in the past ≈4,500 years. In particular GLOBETROTTER assumes that each admixture event occurs as pulses (i.e. over short time periods) between two or more sources. Violations of this (e.g. continuous migration) typically results in inferred dates being biased towards more recent intermixing (Hellenthal et al., 2014).

For inference, GLOBETROTTER uses two (interlocking) steps that each use different CHROMOPAINTER paintings. The first step infers the genetic make-up of the putative admixing source groups and requires each surrogate and target individual to be painted using the same (or a very similar) set of donors. Here we used the "chunklengths" file from the **All-donors** CHROMOPAINTER painting, i.e. as the "input.file.copyvectors" GLOBETROTTER input file. The second step infers the date of admixture, for which we used ten CHROMOPAINTER painting samples per haploid genome for each target group individual to infer the decay of linkage disequilibrium among painted segments that is attributable to admixture. Here we used a slightly different painting, where each target group individual was not allowed to match to other individuals from their same group (but otherwise used the same donors from the **All-donors** analysis). This is because individuals typically match large segments of their genome to other individuals from their own group, and such "self-matching" segments are discarded by GLOBETROTTER when inferring dates, often substantially attenuating the admixture signal. While we could also use this same painting for the first step that infers the genetic make-up of the admixing source groups, for each target group tested we would then have had to re-paint every surrogate group similarly excluding that target group's individuals as donors. For computational simplicity we instead used the same **All-donors** painting for each target group, which previous work suggests makes little difference in practice for these sample sizes (Hellenthal et al., 2014).

As these GLOBETROTTER analyses are designed to infer the ancestral history (in this case admixture history) of the target group, the target group should exhibit genetic homogeneity across its individuals. Therefore, from each DRC ethnicity (target group) we removed individuals that were outliers relative to other group members in the fineSTRUCTURE tree. This led to removing five Lele, two Tetela, three Kete and two Luluwa. We also split the Kete into two groups: Northern Kete (Kete_N) and Southern Kete (Kete_S), given that individuals clustered separately in a manner that corresponded closely with their recorded geographic origins (Section S6).

For each target group, we inferred admixture dates using the default LD decay curve range of 1-50cM and bin size of 0.1cM when considering the distance between painted haplotype segments. We used five iterations of GLOBETROTTER's alternating source composition and admixture date inference (i.e. num.mixing.iterations: 5) and 100 bootstrap re-samples of target individuals to infer 95% confidence intervals (CIs) around the point estimates of the date of admixture. Furthermore, in each test, GLOBETROTTER was run twice, once using the option Nullind:0 and once with Nullind:1 to assess the effect of standardizing against a pseudo (null) individual, an approach designed to account for spurious signals of linkage disequilibrium that are not attributable to admixture (Hellenthal et al., 2014). Results are reported only for target groups who had squared correlation (r^2) ≥ 0.2 and removing any date estimates for which a

date of 1 generation was inferred for any bootstrap re-sample under the Nullind:1 analysis, mimicking previous work (Hellenthal et al., 2014). We report results for Nullind:1 in Figure 2c of the main text and in Table S15, highlighting with an asterisk in all GLOBETROTTER tables any results that should be interpreted with caution due to having discrepancies between Nullind:1 and Nullind:0 results, either in having non-overlapping 95% CIs for date estimates or inferring different types of admixture event (e.g. one-date versus multiple-dates, etc). In general we consider results from Nullind:1 to be more reliable given Hellenthal et al. (2014) identified that population specific drift (e.g. due to bottlenecks) in the target population can affect the ability to accurately infer admixture dates under Nullind:0.

We found evidence of admixture in nine DRC groups. For Kuba, we inferred multiple ($\geq$2) dates of admixture, for which GLOBETROTTER attempts to characterize two events. In these example curves, the best model fit is one that supports two independent exponential distributions to the data, supporting the presence of two different rates of LD decay (Figure S12). This provides evidence for multiple dates of admixture (red line versus green) with Luluwa-GWD, Luluwa-Dekese and Luluwa-Luba depicting the decay curves of a more recent event and the GWD-GWD decay curve showing a signal of an additional older event. This older inferred event dates to 267CE (95% CI: 125BCE-939CE), between sources best represented by Songe and Tshokwe of the DRC (Table S15). Interestingly, the more recent inferred event dates to $\approx$1723CE (95% CI: 1667-1891CE), falling directly within the period of the Kuba Kingdom and with inferred contributions from sources similar in genetic make-up to several DRC groups: Luba, Luntu, Luluwa, Kete, Lele, Lubakat and Tetela. This is a more recent event than any other admixture events we inferred in eight other DRC groups, which ranged in point estimates from 97BCE to 1611CE (Table S15). This inferred recent event in the Kuba is consistent with migration into the Kuba Kingdom increasing the genetic relatedness observed between present-day Kuba and other DRC groups (Figure 2b, Tables S12, S13, S14).

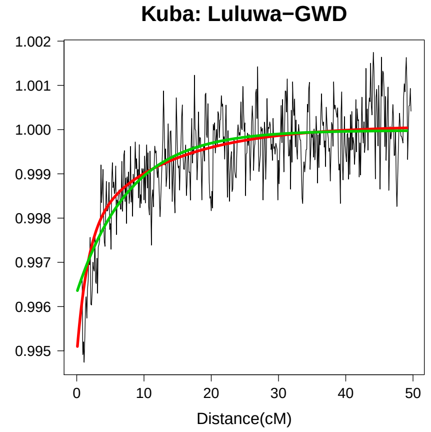

(a) r_1^2=0.609; $\tilde{r}_2^2$=0.06

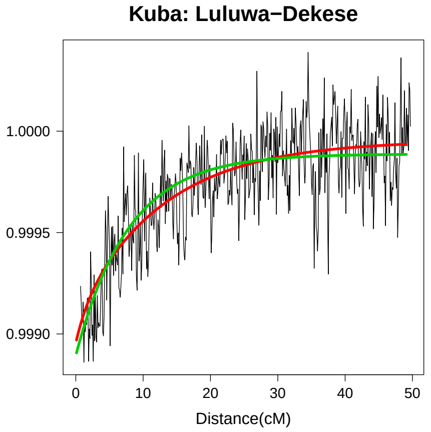

(b) r_1^2=0.629; $\tilde{r}_2^2$=0.02

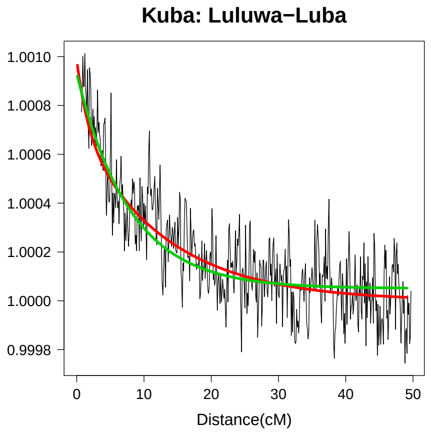

(c) r_1^2=0.732; $\tilde{r}_2^2$=0.04

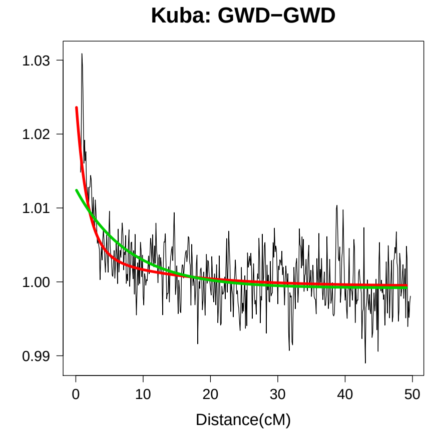

(d) r_1^2=0.409; $\tilde{r}_2^2$=0.16

Figure S12: GLOBETROTTER coancestry curves testing for admixture in Kuba individuals using all groups, including other DRC individuals, as surrogates to admixing sources in the *DRC-all-world* dataset. The black lines give the (scaled) probability (y-axis) that two segments within a Kuba individual, on average, are inferred to be most recently related ancestrally to the two surrogate groups given in the title, versus the genetic distance (x-axis) between the two segments' midpoints. Increasing curves indicate that the two surrogates are representing *different* admixing sources, while decreasing curves indicate that the two surrogates are representing *the same* admixing source. Green lines give the best-fitting exponential model to these data assuming a single date of admixture, while red lines give the best-fitting sum of two exponential models to these data assuming two dates of admixture. Below each plot is r_1^2, the r^2 between the green and black lines measuring the fit of a single date, and $\tilde{r}_2^2 \equiv (r_2^2 - r_1^2)/(1 - r_1^2)$ showing the increased fit when adding a second date (with r_2^2 the r^2 between the red and black lines).

Inference:one-date-admixture									
Target	gens	year	r^2	%.S1	best.S1	S1>10%	%.S2	best.S2	S2>10%
Bindi	17(12-24)	1471CE(1275CE-1611CE)	0.56	0.25	Kongo	Kete_S(0.12)Dinga(0.17)Kuba(0.2)Tetela(0.3)	0.75	Luba	Kuba(0.12)Mfuya(0.17)Luntu(0.54)
Dekese*	43(30-61)	743CE(239CE-1107CE)	0.34	0.23	Kongo	YRI(0.15)Songe(0.13)Lele(0.12)	0.77	Pende	Pende(0.1)Dinga(0.12)Tetela(0.13)Kuba(0.43)
Kete_N*	29(22-40)	1135CE(827CE-1331CE)	0.65	0.43	Luluwa	Luluwa(0.85)	0.57	Kuba	Kuba(0.79)
Kete_S	24(13-35)	1275CE(967CE-1583CE)	0.46	0.19	Kongo	Dinga(0.18)Mbala(0.21)	0.81	Songe	Songe(0.12)Kuba(0.15)Mbala(0.17)Nyambi(0.24)
Lele*	72(60-89)	69BCE(267CE-545BCE)	0.58	0.1	YRI	ESN(0.11)MSL(0.19)	0.9	Pende	Dinga(0.40)Kuba(0.42)
Luluwa*	22(18-25)	1331CE(1247CE-1443CE)	0.87	0.24	Pende	Kete_S(0.15)Kuba(0.31)Lubakat(0.44)	0.76	Luntu	Kuba(0.14)Mfuya(0.24)Luntu(0.42)
Luntu	22(15-26)	1331CE(1219CE-1527CE)	0.73	0.08	Kongo	Nzime_Cam(0.13)ESN(0.18)Bindi(0.38)	0.92	Luluwa	Luluwa(0.94)
Mbala*	45(30-73)	687CE(1107CE-97BCE)	0.26	0.07	YRI	MSL(0.12)YRI(0.30)	0.93	Sala	Kete_S(0.12)Lubakat(0.15)Sala(0.67)
Inference:multiple-date-admixture									
Target	gens.date1	year.date1	r^2	%.date1.S1	best.date1.S1	date1.S1>10%	%.date1.S2	best.date1.S2	date1.S2>10%
Kuba	8(2-10)	1723CE(1667CE-1891CE)	0.78	0.48	Luba	Luba(0.13)Luntu(0.14)Luluwa(0.35)	0.52	Pende	Kete_S(0.1)Lele(0.1)Kete_N(0.11)Lubakat(0.22)Tetela(0.24)
	gens.date2	year.date2		%.date2.S1	best.date2.S1	date2.S1>10%	%.date2.S2	best.date2.S2	date2.S2>10%
	60(36-74)	267CE(125BCE-939CE)		0.33	Songe	SUDANESE(0.1)Luluwa(0.41)	0.67	Tshokwe	Luntu(0.11)Kete_S(0.13)Mfuya(0.27)Lele(0.37)

Table S15: GLOBETROTTER results for Nullind:1 for each DRC group ("Target") in the *DRC-all-world* dataset based on the **All-donors** analysis, testing for admixture in each DRC group using all DRC and all other world-wide groups as surrogates to the putative admixing sources. Target groups are split according to whether one date (Inference:one-date-admixture) or multiple dates of admixture (Inference:multiple-date-admixture) were inferred. The date of admixture is provided in generations ("gens") and years, with bootstrap 95% CIs given in parenthesis, and with generations converted to years using the formula: $1975-(g+1)\times 28$, which reflects how the average birthdate of study participants was $\approx$1975. The maximum r^2 across all surrogate pairs describing how well the linkage disequilibrium decay curve of the data fits to the exponential model expected under the inferred admixture event(s) is provided. The inferred proportion (%.S1, %.S2) and best matching surrogate (best.S1, best.S2) for each inferred source of each admixture event are provided, as is the more detailed inferred composition of each source (S1 and S2) as a mixture of surrogates (only surrogates contributing >10% are shown). Those results marked with an asterisk (*) in the first column should be interpreted with caution due to discrepencies (i.e. differing inference in the type of event or non-overlapping date estimates) between Nullind:1 and Nullind:0 results.

S10 Description of global datasets

In order to infer the relationship of our DRC samples to other populations within and outside Africa, we performed two dataset merges that included populations from around the world, with a focus on capturing African genetic diversity in particular. In these datasets the non-DRC populations are identical but the DRC individuals included differ based on the genotyping array used, so that one dataset maximizes the number of individuals and one maximizes the number of overlapping SNPs. In both cases, and as for the *DRC-only* dataset, all data was quality checked in PLINK(Chang et al., 2015) to exclude SNPs with a missing genotype rate of >10%, minor allele frequency of <1%, and related individuals were excluded based on a PIHAT coefficient >0.2. This resulted in the following datasets:

- *DRC-world* - DRC genotype data from the first collection (ftDNA) comprising 151 DRC individuals from 14 ethnic groups and an additional 3,060 individuals from worldwide groups. The combined merge comprised 606,925 autosomal overlapping SNPs.
- *DRC-all-world* - as in *DRC-world* but including DRC genotype data from both collections (ftDNA and 23andMe), resulting in a final merge of 3,753 individuals after QC, genotyped across 213,164 autosomal overlapping SNPs.

The global populations used, together with the publications in which they were first released, are provided in Table S16 and Figure S13.

Region/Source	Population	N.inds	Region/Source	Population	N.inds
1KGP (The 1000 Genomes Project Consortium, 2015)	Gambia(GWD)	112	Pygmy, Cameroon, Gabon (Patin et al., 2014)	Baka_Cam(BakC)	58
	Iberian(IBS)	107		Nzime_Cam(Nzime)	53
	Tuscan(TSI)	106		Bakiga(Bak)	35
	Japan(JPT)	104		Batwa(Bat)	27
	Puerto Rican(PUR)	104		Bongo_GabS(BonGS)	24
	Han(CHB)	103		Bongo_GabE(BonGE)	22
	Yoruba(YRI)	101		Nzebi_Gab(Nzebi)	20
	Finnish(FIN)	99		Baka_Gab(BakG)	16
	Indian Telugu(ITU)	98	Khoe-san, sub-Saharan, Bantu speakers (Schlebusch et al., 2012)	Khomani(Kho)	39
	Vietman(KHV)	98		ColouredColesberg(ColC)	20
	Southern Han(CHS)	97		ColouredWellington(ColW)	20
	Gujarati(GIH)	96		Karretjie(Karr)	20
	Sri Lankan(STU)	96		Nama(Nama)	20
	African Caribbeans(ACB)	95		SEBantu(SEBan)	20
	Esan Nigeria(ESN)	95		Xun(Xun)	19
	Colombians(CLM)	93		Juhoansi(Juh)	18
	Utah(CEU)	91		Khwe(Khwe)	17
	Punjabi(PJL)	86		GuiGhanaKgal(GuiG)	15
	British(GBR)	85		SWBantu(SWBan)	12
	Bengali(BEB)	83	DRC-all-world	DRC	693
	Dai(CDX)	82	DRC-world	DRC	151
	Luhya(LWK)	79			
	Peruvians(PEL)	76			
	Mende(MSL)	69			
	Mexican(MXL)	55			
	African Americans(ASW)	45			
Ethiopia, Sudan, Somali (Pagani et al., 2012)	Amhara(AMH)	25			
	AriCultivator(ARIc)	24			
	Anuak(ANU)	23			
	Somali(SOM)	23			
	Sudanese(SUD)	22			
	Oromo(ORO)	21			
	Tygray(TYG)	21			
	Gumuz(GUM)	19			
	ESomali(ESOM)	17			
	AriBlacksmith(ARIb)	15			
	Afar(AFA)	12			
	Wolayta(WOL)	8			

Table S16: Description and number of QC'd individuals (N.inds) for the global datasets: *DRC-world* (3,211 individuals, 606,925 SNPs) and *DRC-all-world* (3,753 individuals, 213,164 SNPs). Full details on the populations included can be obtained from the publications in which they were originally released (Region/Source) and are also provided in Figure S13. For the DRC the total number of individuals is provided for each dataset with the full description provided in Table S1 and Figure 1 of the main text.

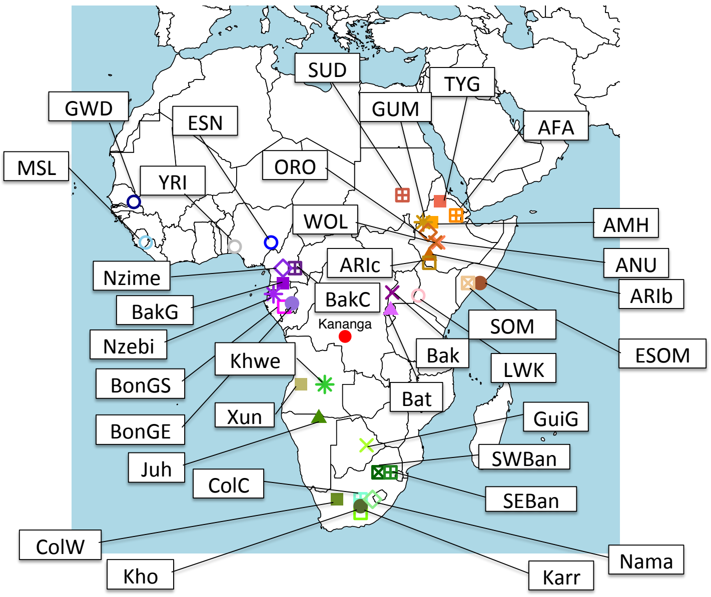

(a)

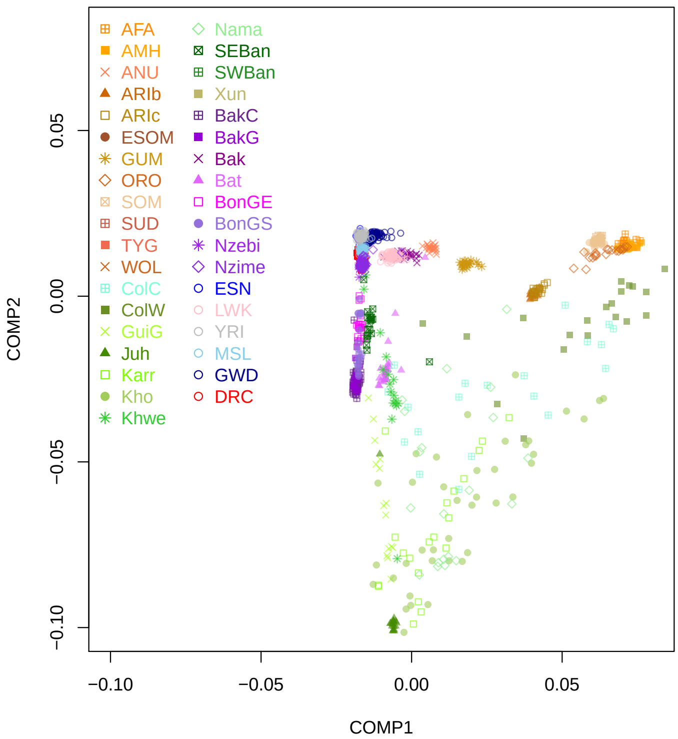

(b)

Figure S13: (a) Map providing locations of African samples included in the *DRC-world* and *DRC-all-world* datasets. (b) PCA analysis of the *DRC-all-world* dataset including only African individuals shown in (a). Symbols are colored according to the publication in which the dataset was originally made available, with Ethiopians, Sudanese and Somalians from Pagani et al. (2012) in orange colors, sub-Saharan African and Bantu speakers from Schlebusch et al. (2012) in greens, Pygmy hunter-gatherers and agriculturalists from Cameroon and Gabon from Patin et al. (2014) in purples and African populations from The 1000 Genomes Project Consortium (2015) in blue (ESN), pink (LWK) and grey (YRI). Novel DRC samples from this study are plotted in red.

S11 DRC individuals' ancestry sharing with non-DRC groups

Using these two global datasets we performed a CHROMOPAINTER analysis as in Section S5.1, but now using all individuals (i.e. not just DRC individuals) as both donors and recipients. We refer to this as the **All-donors** analysis, see main text **Methods**. However, we also performed an alternative CHROMOPAINTER analysis where we matched DNA patterns in DRC individuals only to those of non-DRC individuals (**non-DRC-donors**). As before, in both cases we initially estimated the CHROMOPAINTER mutation (θ) and switch rate (Ne) parameters across 4 chromosomes 1,4,15,22 on every 10th individual. This gave estimates of: (i) *DRC-world*, **All-donors** (θ = 0.0005785; Ne = 230.020), (ii) *DRC-world*, **Non-DRC-donors** (θ = 0.0005863; Ne = 235.1427), (iii) *DRC-all-world*, **All-donors** (θ = 0.0008786; Ne = 181.873), (iv) *DRC-all-world*, **non-DRC-donors** (θ = 0.0009415; Ne = 202.8686). CHROMOPAINTER was then run with fixed estimates across all autosomes to infer a haplotype sharing profile for each individual and worldwide group by measuring the amount of DNA contributed by members of each donor group.

Relative to comparing haplotype patterns in DRC individuals to each other (i.e. the **All-donors** analysis), the **non-DRC-donors** analysis should reflect the sharing of ancestors further back in time, and hence provides a glimpse of the more ancient ancestry of the DRC. Consistent with this, on average DRC individuals match shorter genetic segments to non-DRC people ($\approx$0.65 cM) relative to DRC people ($\approx$1.76cM), highlighting how this analysis can capture features of older genetic relationships.

As in Section S5, we assess the differences in inferred painting profiles using TVD and permutation re-sampling. Individuals from different DRC ethnic groups exhibit extremely similar patterns of haplotype sharing to non-DRC groups under this analysis, which was not the case in the **All-donors** analysis, eg. compare upper and lower triangles of Figure S14. In particular many of the genetic differences between ethnic groups observed under the **All-donors** analysis (Tables S9-S10, Figure S6) are mitigated under this **non-DRC-donors** analysis (Tables S17-S18, Figure S15). Taken together this suggests that DRC groups sampled here share a common history relative to outside groups, and that detectable genetic differences among DRC groups today likely result from relatively recent isolation among them (van Dorp et al., 2015).

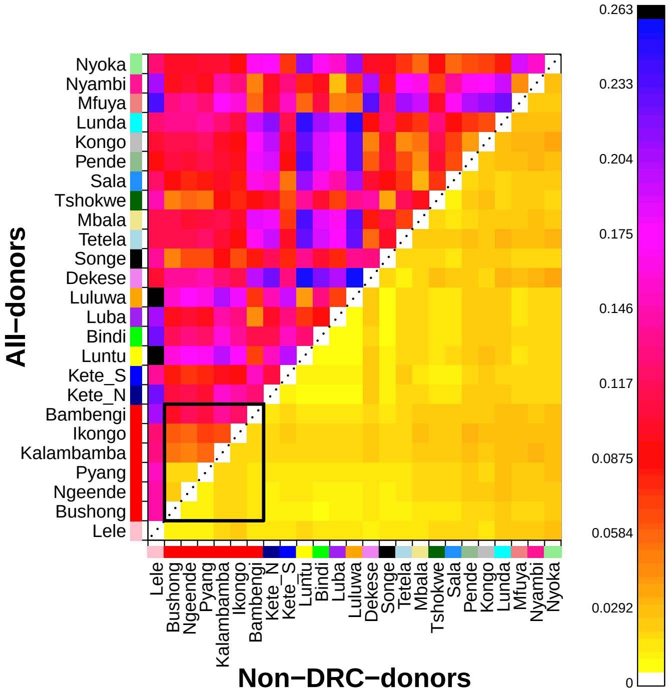

(a)

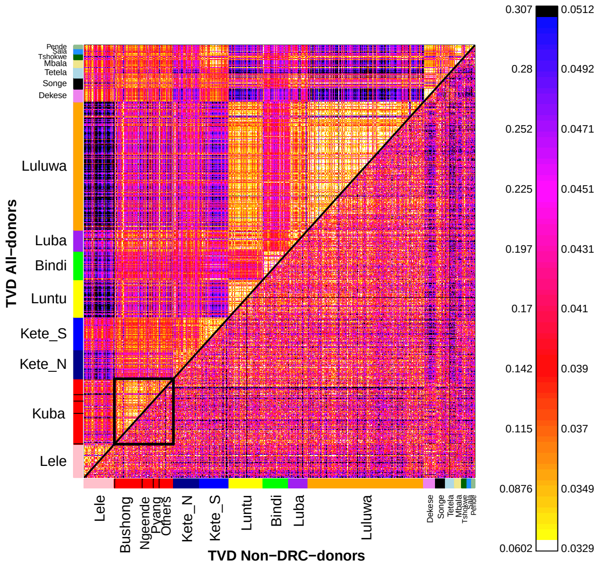

(b)

Figure S14: Pairwise TVD under **All-donors** (upper triangle) and **Non-DRC-donors** analyses (lower triangle) across DRC groups based on the *DRC-all-world* dataset as in Table S17. (a) provides the total values, with scale at right, when combined across individuals in each group (with $\geq$ 3 individuals) (b) provides the values per individual ordered by each group (with $\geq$ 5 individuals) with scale at right providing the scores for **All-donors** (left of scale) and **Non-DRC-donors** (right of scale). In both cases axes are colored by the color assignment also provided in main text Figure 1 and Kuba sub-groups are bordered in black.

	TVD non-DRC-donors														
**Group**	**Kuba_Bushong**	**Kuba_Ngeende**	**Kuba_Pyang**	**Kuba_All**	**Lele**	**Kete_N**	**Kete_S**	**Luntu**	**Bindi**	**Luba**	**Luluwa**	**Dekese**	**Songe**	**Tetela**	**Mbala**
**Kuba_Bushong**	0.041(0.037-0.045)	0.041(0.039-0.045)	0.041(0.038-0.044)		0.041(0.037-0.045)	0.041(0.038-0.043)	0.042(0.037-0.047)	0.041(0.037-0.046)	0.041(0.038-0.044)	0.041(0.036-0.045)	0.041(0.038-0.046)	0.041(0.037-0.045)	0.041(0.038-0.045)	0.044(0.039-0.056)	0.043(0.04-0.047)
**Kuba_Ngeende**	0.041(0.039-0.045)	0.042(0.039-0.045)	0.041(0.038-0.044)		0.042(0.038-0.046)	0.041(0.038-0.045)	0.043(0.039-0.048)	0.042(0.038-0.046)	0.041(0.039-0.044)	0.041(0.038-0.045)	0.042(0.038-0.047)	0.042(0.039-0.046)	0.042(0.039-0.046)	0.045(0.04-0.057)	0.044(0.041-0.049)
**Kuba_Pyang**	0.041(0.038-0.044)	0.041(0.038-0.044)	0.041(0.038-0.044)		0.04(0.037-0.044)	0.041(0.038-0.044)	0.043(0.038-0.048)	0.042(0.038-0.046)	0.041(0.038-0.044)	0.041(0.038-0.046)	0.042(0.038-0.047)	0.041(0.037-0.044)	0.042(0.038-0.047)	0.044(0.038-0.055)	0.044(0.04-0.047)
**Kuba_All**				0.041(0.038-0.045)	0.041(0.037-0.045)	0.041(0.038-0.044)	0.042(0.038-0.047)	0.042(0.037-0.045)	0.041(0.039-0.044)	0.041(0.037-0.045)	0.042(0.038-0.046)	0.041(0.038-0.045)	0.042(0.039-0.046)	0.044(0.039-0.055)	0.044(0.041-0.048)
**Lele**	0.041(0.037-0.045)	0.042(0.038-0.046)	0.04(0.037-0.044)	0.041(0.037-0.045)	0.039(0.036-0.043)	0.042(0.039-0.045)	0.043(0.038-0.049)	0.043(0.039-0.048)	0.042(0.039-0.046)	0.042(0.037-0.048)	0.043(0.038-0.048)	0.04(0.036-0.044)	0.042(0.039-0.046)	0.044(0.039-0.055)	0.042(0.039-0.046)
**Kete_N**	0.041(0.038-0.043)	0.041(0.038-0.045)	0.041(0.038-0.044)	0.041(0.038-0.044)	0.042(0.039-0.045)	0.04(0.036-0.044)	0.041(0.037-0.046)	0.04(0.035-0.043)	0.04(0.037-0.043)	0.039(0.036-0.043)	0.04(0.036-0.044)	0.044(0.039-0.048)	0.041(0.037-0.044)	0.046(0.04-0.059)	0.044(0.041-0.049)
**Kete_S**	0.042(0.037-0.047)	0.043(0.039-0.048)	0.043(0.038-0.048)	0.042(0.038-0.047)	0.043(0.038-0.049)	0.041(0.037-0.046)	0.041(0.037-0.044)	0.041(0.037-0.046)	0.041(0.038-0.045)	0.041(0.037-0.045)	0.042(0.038-0.046)	0.046(0.04-0.051)	0.042(0.039-0.045)	0.048(0.042-0.06)	0.042(0.039-0.047)
**Luntu**	0.041(0.037-0.046)	0.042(0.038-0.046)	0.042(0.038-0.046)	0.042(0.037-0.045)	0.043(0.039-0.048)	0.04(0.035-0.043)	0.041(0.037-0.046)	0.039(0.035-0.043)	0.04(0.037-0.044)	0.039(0.035-0.043)	0.04(0.036-0.044)	0.045(0.039-0.049)	0.041(0.037-0.045)	0.047(0.04-0.06)	0.045(0.041-0.05)
**Bindi**	0.041(0.038-0.044)	0.041(0.039-0.044)	0.041(0.038-0.044)	0.041(0.039-0.044)	0.042(0.039-0.046)	0.04(0.037-0.043)	0.041(0.038-0.045)	0.04(0.037-0.044)	0.04(0.037-0.044)	0.039(0.035-0.044)	0.04(0.036-0.044)	0.044(0.038-0.047)	0.041(0.037-0.044)	0.046(0.039-0.058)	0.044(0.04-0.048)
**Luba**	0.041(0.036-0.045)	0.041(0.038-0.045)	0.041(0.038-0.046)	0.041(0.037-0.045)	0.042(0.037-0.048)	0.039(0.036-0.043)	0.041(0.037-0.045)	0.039(0.035-0.043)	0.039(0.035-0.044)	0.039(0.035-0.043)	0.039(0.036-0.044)	0.045(0.04-0.048)	0.04(0.037-0.044)	0.047(0.04-0.06)	0.045(0.041-0.049)
**Luluwa**	0.041(0.038-0.046)	0.042(0.038-0.047)	0.042(0.038-0.047)	0.042(0.038-0.046)	0.043(0.038-0.048)	0.04(0.036-0.044)	0.042(0.038-0.046)	0.04(0.036-0.044)	0.04(0.036-0.044)	0.039(0.036-0.044)	0.04(0.037-0.044)	0.045(0.04-0.049)	0.041(0.037-0.045)	0.047(0.04-0.06)	0.045(0.042-0.049)
**Dekese**	0.041(0.037-0.045)	0.042(0.039-0.046)	0.041(0.037-0.044)	0.041(0.038-0.045)	0.04(0.036-0.044)	0.044(0.039-0.048)	0.046(0.04-0.051)	0.045(0.039-0.049)	0.044(0.038-0.047)	0.045(0.04-0.048)	0.045(0.04-0.049)	0.038(0.034-0.042)	0.044(0.038-0.05)	0.042(0.036-0.054)	0.044(0.039-0.049)
**Songe**	0.041(0.038-0.045)	0.042(0.039-0.046)	0.042(0.038-0.047)	0.042(0.039-0.046)	0.042(0.039-0.046)	0.041(0.037-0.044)	0.042(0.039-0.045)	0.041(0.037-0.045)	0.041(0.037-0.044)	0.04(0.037-0.044)	0.041(0.037-0.045)	0.044(0.038-0.05)	0.042(0.038-0.045)	0.046(0.04-0.058)	0.045(0.042-0.05)
**Tetela**	0.044(0.039-0.056)	0.045(0.04-0.057)	0.044(0.038-0.055)	0.044(0.039-0.055)	0.044(0.039-0.055)	0.046(0.04-0.059)	0.048(0.042-0.06)	0.047(0.04-0.06)	0.046(0.039-0.058)	0.047(0.04-0.06)	0.047(0.04-0.06)	0.042(0.036-0.054)	0.046(0.04-0.058)	0.046(0.04-0.056)	0.048(0.042-0.052)
**Mbala**	0.043(0.04-0.047)	0.044(0.041-0.049)	0.044(0.04-0.047)	0.044(0.041-0.048)	0.042(0.039-0.046)	0.044(0.041-0.049)	0.042(0.039-0.047)	0.045(0.041-0.05)	0.044(0.04-0.048)	0.045(0.041-0.049)	0.045(0.042-0.049)	0.044(0.039-0.049)	0.045(0.042-0.05)	0.048(0.042-0.052)	0.041(0.038-0.047)

Table S17: Pairwise TVD under the **non-DRC-donors** analyses across labelled DRC groups with >10 individuals, including the Kuba sub-groups the Bushong, Ngeende and Pyang. Mean and 5-95% CIs are provided for each comparison where appropriate.

Group	Luluwa	Kuba	Luntu	Kete_S	Lele	Bindi	Kete_N	Luba	Dekese	Songe	Tetela	Mbala
**Luluwa**	NA	1	0.552	1	1	0.992	0.189	0.048	1	0.448	1	1
**Kuba**	1	NA	1	1	1	1	1	1	1	0.877	0.999	1
**Luntu**	0.552	1	NA	1	1	0.988	0.711	0.754	1	0.801	1	1
**Kete_S**	1	1	1	NA	1	1	1	1	1	0.995	1	0.996
**Lele**	1	1	1	1	NA	1	1	1	1	1	0.998	1
**Bindi**	0.992	1	0.988	1	1	NA	0.591	0.775	1	0.203	1	1
**Kete_N**	0.189	1	0.711	1	1	0.591	NA	0.326	1	0.705	1	1
**Luba**	0.048	1	0.754	1	1	0.775	0.326	NA	1	0.147	1	1
**Dekese**	1	1	1	1	1	1	1	1	NA	1	0.892	1
**Songe**	0.448	0.877	0.801	0.995	1	0.203	0.705	0.147	1	NA	1	0.998
**Tetela**	1	0.999	1	1	0.998	1	1	1	0.892	1	NA	1
**Mbala**	1	1	1	0.996	1	1	1	1	1	0.998	1	NA

Table S18: The proportion of times the true TVD between any pairwise comparison of DRC groups with >10 individuals is greater than TVD in 1000 random permutations of ethnic label in each comparison, under the **Non-DRC-donors** painting applied to the *DRC-all-world* dataset.

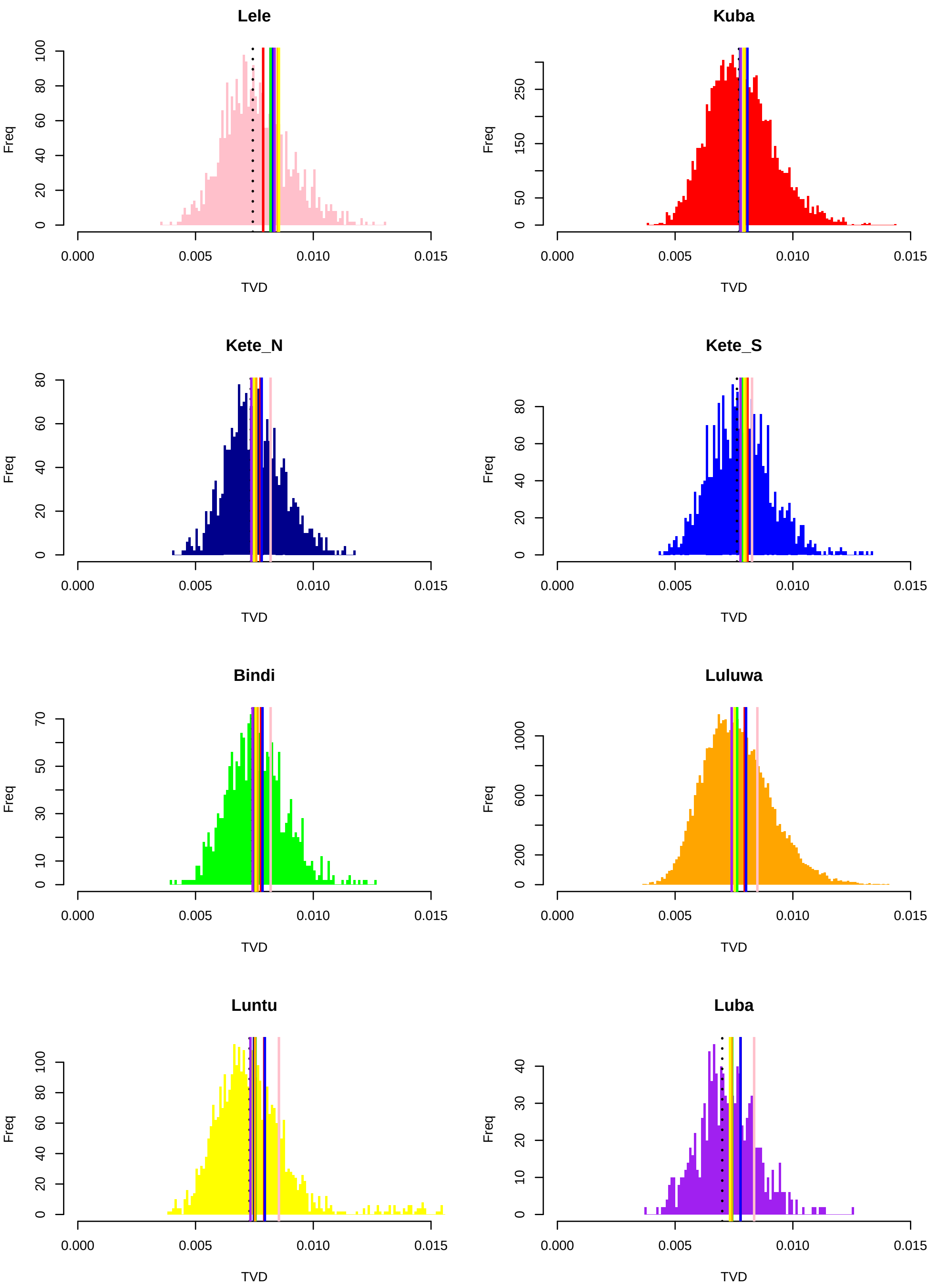

Figure S15: Histograms of TVD values within ethnic groups with a sample size >30 under the **non-DRC-donors** analysis using the *DRC-all-world* dataset. Colors correspond to different ethnic groups as defined in main text Figure 1 and given in the header of each plot. Colored horizontal bars give the mean TVD between each group with every other ethnic group whilst the black dashed bar gives the mean TVD within group.

S11.1 Mixture modeling of ancestry proportions

To understand the ancestry of the sampled DRC groups in the context of these global datasets, we used SOURCEFIND (Chacon-Duque et al., 2018) to describe the ancestry of a target population as a mixture of surrogate groups. To do so, SOURCEFIND assumes the overall amount of DNA segments that each DRC ethnicity matches to each of K labeled populations, as inferred by CHROMOPAINTER, follows a multinomial distribution with K parameters. Each parameter k of this multinomial is described as a linear mixture of the proportions of DNA that the surrogate groups match to labeled population k, with these mixture coefficients the same across all K parameters and summing to 1 across all surrogates. SOURCEFIND uses MCMC to infer the mixture coefficients, under a Bayesian framework that puts a truncated Poisson prior on the number of surrogate groups that have mixing coefficients >0 at each iteration. SOURCEFIND also allows for a "self-matching" term that reflects the additional amount of haplotype matching between members of the target group exceeding that predicted under the inferred mixture of the surrogate groups.

This mixture modelling approach has advantages over using the raw painting profiles inferred by CHROMOPAINTER to describe the target group's ancestry, as the raw painting profiles can be subject to biases as a result of sample size differences amongst donor groups, among other issues. The output of SOURCEFIND is the proportion of DNA for which each target group, on average, shares most recent ancestry with each surrogate group. While these inferred proportions of ancestry can be indicative of admixture in the target group, we caution strongly against such an interpretation in general, since many other processes can lead to identical ancestry proportion inference. In addition, SOURCEFIND allows for a "self-matching" term that reflects the additional amount of CHROMOPAINTER-inferred haplotype matching between members of the target group exceeding that predicted under the inferred mixture of the surrogate groups. We consider this "self-matching" term to be the proportion of ancestry that the target group shares most recently with its own groups members.

We performed SOURCEFIND analyses to infer the contributions to each DRC ethnicity under two different settings applied to the *DRC-all-world* dataset:

(a) all populations are used as surrogates, using the haplotype sharing patterns inferred under the **All-donors** analysis and including a "self-matching" term

(b) only populations outside of the DRC are used as surrogates, using the haplotype sharing patterns inferred under the **non-DRC-donors** analysis

Analysis (a) aims to reveal proportions of ancestry shared at recent time periods, by capturing recent ancestry sharing among DRC groups whilst (b) aims to characterize the ancestral history of these groups at deeper time-scale, by capturing recent ancestry sharing between each DRC group and groups outside of the DRC, with whom they should share more distant relatives. Results under (a) are provided in Figure 3c and Tables S19, while results under (b) are provided in Figure 3b and Table S20. In all cases we ran SOURCEFIND for 200,000 iterations of Markov-Chain-Monte-Carlo (MCMC), discarding the first 50,000 iterations as burn-in and sampling posterior mixing coefficients every 5000 iterations thereafter. We assumed a maximum of 8 surrogates could have coefficient >0 at each MCMC iteration, with an a priori expectation of 4 surrogates.

Notably, while inference under (a) varied considerably across DRC ethnicities, inferences under (b) consistently matched DNA patterns in each DRC group primarily to the Nzebi of Gabon (Nzebi_Gab), the Nzime of Cameroon (Nzime_Cam), a Nigerian group (Yoruba or Esan), Luhya of Kenya (LWK) and South East Bantu speakers (SEBantu).

Group	Luluwa	Tetela	Luba	Kuba	Songe	Luntu	Mfuya	Bunde	Tshokwe	Kongo	Pende	Nyambi	Lubakat	Dinga	Lele	Moyisambo	Kete	Mongo	Bena_Konji	Bindi	Mbala
Kuba	12.03(0-37.28)	2.73(0-29.6)	8(0-62.82)	3.03(2-4)	6.83(0-50.28)	4.23(0-28.65)	3.23(0-33.5)	2(0-23.3)	7.87(0-75.22)	1.2(0-14.85)	38.8(0-67.28)	3.67(0-34.32)	2.07(0-19.27)	1.97(0-17.2)	0.97(0-8.27)	0.23(0-1.92)	0.6(0-6.3)	0(0-0)	0(0-0)	0(0-0)	0(0-0)
Lele	0(0-0)	4.47(0-27.02)	0(0-0)	6.37(0-23)	0(0-0)	0.07(0-0.55)	0.07(0-0.55)	0.03(0-0.27)	0(0-0)	28.6(0-83.02)	47.2(0-89.82)	0.37(0-4.37)	0(0-0)	1.67(0-16.12)	8.67(7.73-9)	1.1(0-10.55)	0(0-0)	0(0-0)	0.23(0-3.27)	0(0-0)	0.07(0-1)
Luluwa	4.43(2-6.27)	0(0-0)	11.03(0-44.28)	0(0-0)	0.13(0-1.1)	65.97(46.17-96.27)	7.43(0-41.3)	0.33(0-4.55)	0(0-0)	0.1(0-1.27)	0(0-0)	8.57(0-45.28)	0.4(0-4.37)	0(0-0)	0(0-0)	0.13(0-1.1)	0.97(0-11)	0.17(0-1.37)	0(0-0)	0(0-0)	0(0-0)
Luba	24.4(0-79.82)	0(0-0)	0(0-0)	1.67(0-15.47)	8.43(0-44.65)	8.63(0-42.28)	11.5(0-61.3)	0(0-0)	2.9(0-23.67)	2.27(0-19.55)	1.57(0-23.28)	21.3(0-91.65)	1.07(0-8.82)	0.2(0-1.65)	0(0-0)	0.43(0-4.55)	2.27(0-18.47)	5(0-27.1)	6.4(0-42.82)	0(0-0)	0.03(0-0.27)
Luntu	91.37(56.08-98)	0(0-0)	3.8(0-41.92)	0.03(0-0.27)	0.27(0-2.37)	2.13(2-3)	0(0-0)	0(0-0)	0.03(0-0.27)	0(0-0)	0(0-0)	0(0-0)	0.1(0-1.27)	0(0-0)	0.03(0-0.27)	0.2(0-2.1)	0(0-0)	1.1(0-12.75)	0.73(0-6.82)	0.07(0-1)	0.1(0-0.82)
Bindi	0.07(0-0.55)	0.43(0-3.57)	54.87(0-91)	3(0-21.57)	0.23(0-2.82)	10.7(0-51.2)	2.2(0-23.37)	2.07(0-20.22)	8.37(0-37.65)	0.67(0-6.57)	4.23(0-30.37)	0.37(0-3.02)	0.1(0-0.82)	0.33(0-2.75)	0.07(0-0.55)	0.3(0-3.82)	1.67(0-15.3)	0(0-0)	0(0-0)	9.47(8.72-10)	0.13(0-1.1)
Kete	0(0-0)	0(0-0)	0(0-0)	14.1(0-55)	1.27(0-13.67)	0(0-0)	0(0-0)	0(0-0)	71.9(1.45-94)	0.07(0-0.55)	0.07(0-0.55)	0.9(0-7.42)	0(0-0)	0.1(0-1.27)	0(0-0)	2.07(0-15.47)	7.1(6-8)	0.5(0-4.12)	0.07(0-0.55)	0(0-0)	1.03(0-11.57)

Table S19: Inferred ancestry percentages between DRC groups (Targets) and global groups (Surrogates), including the DRC, inferred using SOURCEFIND (Chacon-Duque et al., 2018). Results are based on the **All-donors** analysis applied to the *DRC-all-world* dataset. Only surrogates with a total contribution >1% to at least one group are provided. Each entry provides the mean and 95% credible intervals across 30 MCMC samples. Results are also provided as a barplot in main text Figure 3c.

Group	Khwe	LWK	Nzebi_Gab	Nzime_Cam	SEBantu	SWBantu	YRI	Bakiga	ESN
Kuba	1.27(0-4)	12.57(7.45-17)	52(33.18-71.82)	7.23(0.73-11.28)	6.3(3.73-11.1)	1.8(0-4)	17.73(8-24.27)	0.83(0-3.82)	0.17(0-1.37)
Lele	1.57(0-3)	11.2(7.45-16.82)	58.03(41.18-74.92)	5.83(0-12)	4.03(0-8)	1.9(0-4)	16.6(6.52-23)	0.27(0-3)	0.57(0-6.02)
Luluwa	0.9(0-2.27)	12.3(4.72-18)	56.23(39.08-76.55)	5.9(0-13.28)	7.13(4.72-10.82)	1(0-3)	14.53(0-21)	1.03(0-5.27)	0.97(0-14.27)
Luba	1(0-2.27)	14.07(7.73-20.82)	53.9(40.62-65.55)	6.47(0-10.28)	6.7(3-11.28)	1.8(0-4)	14.23(0-21)	0.73(0-4.27)	1.1(0-10.37)
Luntu	1.03(0-2)	13.33(7.45-18.55)	54.63(33.45-73.82)	6.63(3-12)	7.5(4.72-10.28)	1.13(0-3)	13.43(0-22.55)	1(0-4.27)	1.27(0-12.55)
Bindi	1.17(0-3)	12.67(8-18)	51.8(37.9-66.82)	7.1(0-13)	7.4(4-11)	1.5(0-4)	17.53(12.72-24.55)	0.83(0-4)	0(0-0)
Kete	1.97(0.73-3)	12.7(8-17.27)	51.87(35.08-66.82)	6.8(0-12)	6.33(2.72-10.1)	2.73(1-4.55)	17.13(10.73-23)	0.47(0-4.27)	0(0-0)

Table S20: Inferred ancestry percentages between DRC groups (Targets) and global groups (Surrogates), excluding the DRC, inferred using SOURCEFIND (Chacon-Duque et al., 2018). Results are based on the **non-DRC-donors** analysis applied to the *DRC-all-world* dataset. Only surrogates with a total contribution >1% to at least one group are provided. Each entry provides the mean and 95% credible intervals across 30 MCMC samples. Results are also provided as a barplot in main text Figure 3b.

S12 Inferring and dating admixture from non-DRC sources into the DRC

Given the lack of differentiation amongst DRC groups in how they relate ancestrally to non-DRC groups (e.g. Figure 3b of the main text, Figure S14, Table S20), we also applied GLOBETROTTER to all 693 DRC individual jointly using the *DRC-all-world* dataset. This analysis assumes that all of our sampled DRC individuals share common recent ancestral origins, and aims to characterize any admixture events that may have occurred in these ancestors. We applied GLOBETROTTER as described in Section S9, though here we used the same painting in the two steps described in that section. In particular we used the **non-DRC-donors** painting for both the painting profiles used for inferring the genetic make-up of the putative admixing source groups and the painting samples used to infer dates of admixture. Here we also excluded the African Caribbeans in Barbados (ACB) and Americans of African Ancestry in SW USA (ASW) from The 1000 Genomes Project Consortium (2015) as surrogates, because these groups are themselves recently admixed, which makes interpreting any inferred matching to them challenging.

We inferred a complex admixture event between multiple sources dated to 265BCE (95% CI: 377BCE-211CE), with an overlapping date range inferred when using fewer DRC individuals but more SNPs (*DRC-world* dataset) (293BCE; 95% CI:433BCE- 155CE, Table S21). The inferred admixture event involves more than two sources mixing at approximately the same time, with each source represented by multiple (overlapping) African surrogate groups (Table S21, Figures S16-S17). The precise genetic make-up of the source groups involved is difficult to characterize using these sampled, present-day surrogate groups, particularly given the older nature of the admixture. However, coancestry curves (Figure S16) broadly suggest one admixing source was related to present-day Nigerian (YRI) groups, with at least two other separate sources with varying relatedness to present-day SEBantu and groups from Cameroon and Gabon.

Due to the complicated nature of this inferred event and difficulty in disentangling the genetic make-up of the admixing groups involved, it is also difficult to assign this admixture to any particular historical event with confidence. Furthermore, we note our inferred date likely reflects more recent migrations at the cost of potentially missing older migrations into the region, due to recent events often acting to “mask” older ones. Subject to these issues, the primary languages of our DRC groups are Bantu in origin, and our findings could be consistent with multiple episodes of intermixing between Bantu-speaking peoples and inhabitants of the DRC, who had already arrived as part of the major Bantu migrations into the Congo Basin, e.g. with multiple migrations occurring close enough in time around 2,000 years ago that GLOBETROTTER cannot distinguish separate dates. Indeed our inferred dates and sources are in concordance with the proposed timing of a late wave of migrations, thought to correspond to the development of metallurgy in Nigeria and a subsequent expansion of individuals with iron-based technologies (Cavalli-Sforza et al., 1994; Vansina, 2006; Pour et al., 2013; Klieman, 2003). They are also consistent with evidence for settlements of iron-using people in Gabon and Congo dated to the end of the first millennium BCE (Ehret, 2001; Peyrot and Oslisly, 1986).

DRC-all-world Dataset									
gen.date	year.date	r2	%.S1	best.S1	S1>10%	%.S2	best.S2	S2>10%
79(62-83)	265BCE(377BCE-211CE)	0.79	0.33	YRI	YRI(0.58),Nzime_Cam(0.12),Nzebi_Gab(0.12),LWK(0.07),LWK(0.06)	0.67	Nzebi_Gab	Nzebi_Gab(0.39),LWK(0.19),SEBantu(0.14),Nzime_Cam(0.12),Bakiga(0.07)
**DRC-world Dataset**								
gen.date	year.date	r2	%.S1	best.S1	S1>10%	%.S2	best.S2	S2>10%
80(64-85)	293BCE(433BCE-155CE)	0.71	0.27	YRI	ESN(0.1),Nzime_Cam(0.13),YRI(0.6)	0.73	Nzebi_Gab	SEBantu(0.11),Nzime_Cam(0.12),LWK(0.19),Nzebi_Gab(0.43)

Table S21: GLOBETROTTER results using *Nullind:1* testing for admixture events shared by all sampled DRC individuals using all non-DRC groups as surrogates, under the **non-DRC-donors** painting. Results are provided for both the *DRC-all-world* and *DRC-world* datasets. The date of admixture is provided in generations ("gens") and years, with bootstrap 95% CIs given in parenthesis, and with generations converted to years using the formula: $1975 - (g + 1) \times 28$, which assumes the average birthdate of study participants was 1975. The maximum r^2 across all surrogate pairs describing the how well the linkage disequilibrium decay curve of the data fits to the exponential model expected under the inferred admixture event(s) is provided. The proportion (%.S1, %.S2) and best matching surrogate (best.S1, best.S2) for each inferred source of each admixture event are provided, as is the more detailed inferred composition of each source (S1 and S2) as a mixture of surrogates (only surrogates contributing >10% are shown).

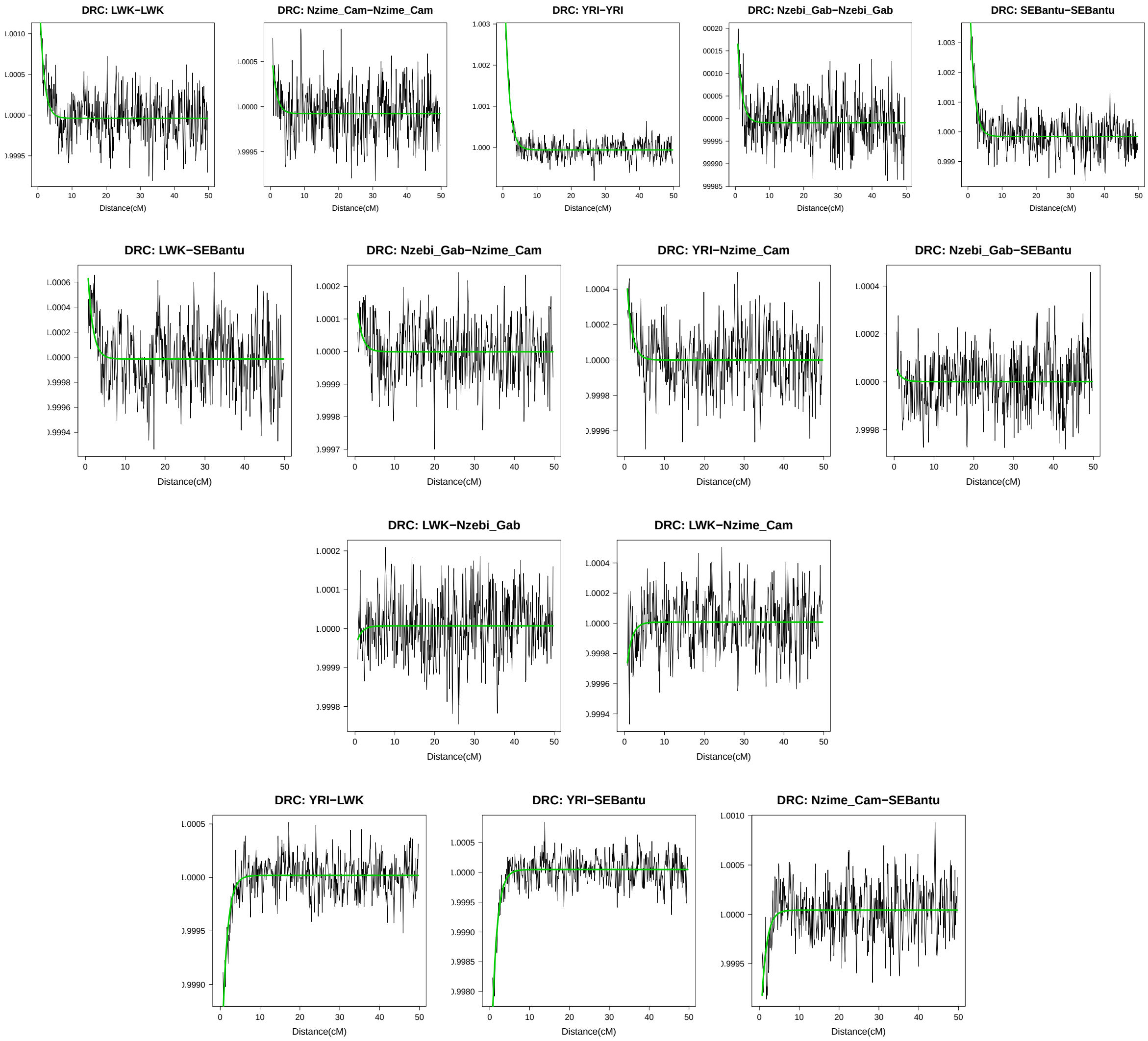

Figure S16: GLOBETROTTER coancestry curves testing for admixture shared by all DRC indivdiuals for the *DRC-all-world* dataset. The black lines give the (scaled) probability (y-axis) that two segments within a DRC individual, on average, are inferred to be most recently related ancestrally to the two surrogate groups given in the title versus the genetic distance (x-axis) between the two segments' midpoints. Green lines give the best-fitting exponential model to these data assuming a single date of admixture. Here the surrogates depicted are the Yoruba of Nigeria (YRI) (The 1000 Genomes Project Consortium, 2015), Luhya of Webuye, Kenya (LWK) (The 1000 Genomes Project Consortium, 2015), Bantu speakers of South East Africa (SEBantu) (Schlebusch et al., 2012), the Nzime of Cameroon (Nzime_Cam) and the Nzebi of Gabon (Nzebi_Gab) (Patin et al., 2014). Increasing curves indicate that the two surrogates are representing *different* admixing sources, while decreasing curves indicate that the two surrogates are representing *the same* admixing source. Therefore, the fact that each combination of {LWK,Nzime_Cam,SEBantu} shows increasing curves demonstrates that >2 sources have intermixed (Hellenthal et al., 2014).

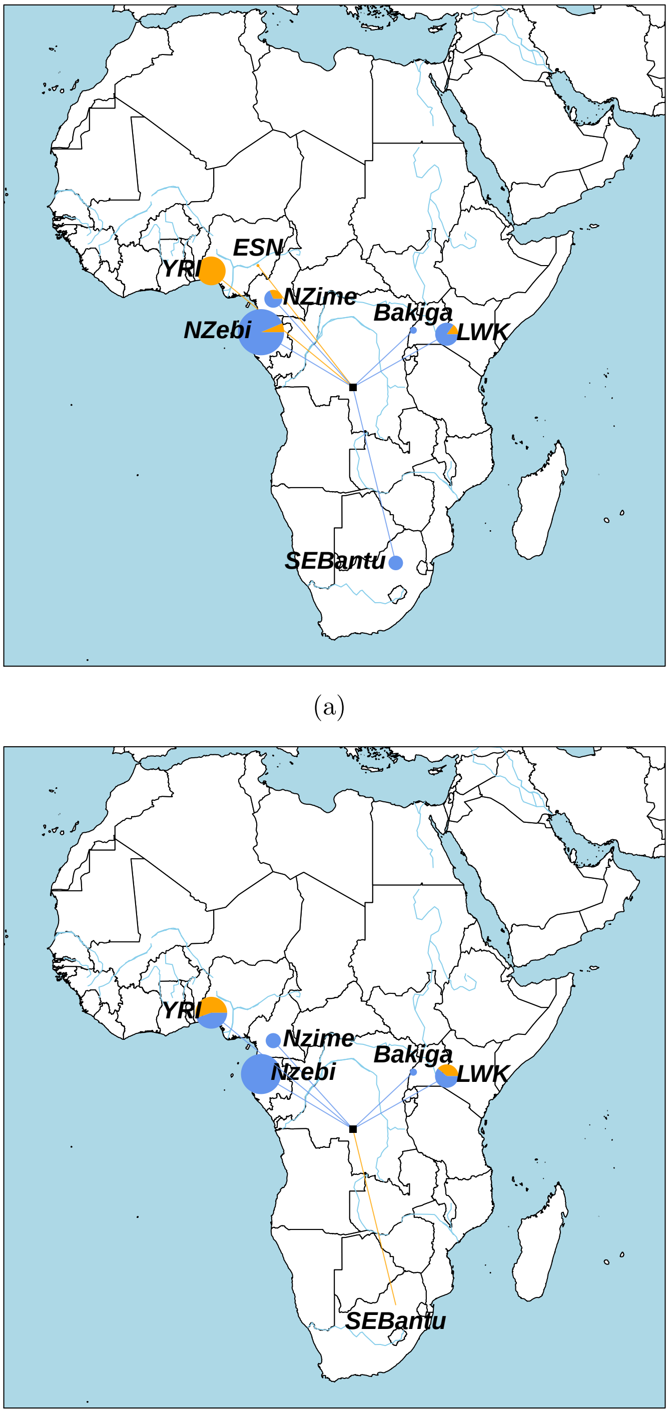

(a)

(b)

Figure S17: Genetic make-up of inferred admixture sources when testing for admixture shared by all DRC individuals using GLOBETROTTER with *null.ind:1*, with one admixing source depicted in orange and the other in blue, for (a) the strongest signaled inferred event and (b) an additional inferred admixture event. Both events are dated to 390BCE (400-190BCE). The size of each pie gives the total inferred contribution from that surrogate to the DRC, with the pie slices depicting the proportion of this contribution that is inferred to reflect DNA inherited from each of the two admixing sources. Only surrogates contributing >5% to the genetic make-up of source1 and source2 are shown. The exact composition of each source is provided in Table S21.

S13 Inferring and dating admixture within Africa

The Kasai region of the DRC is an important area for understanding the expansion of the Bantu speaking people, given Western Central Africa is the widely purported starting point for the expansion of Bantu languages, together with agriculture, into East and Southern Africa, beginning about 5000 years ago (Klieman, 2003; Eggert, 1992). Previous studies (Tishkoff et al., 2009; de Filippo et al., 2012; Li et al., 2014; Busby et al., 2016; Patin et al., 2017) have lacked genome-wide data from the DRC. Thus, to expand upon recent work documenting Bantu migrations in East and South Africa (Busby et al., 2016; Patin et al., 2017), we also tested for admixture in each of our non-DRC African groups while using the DRC as a surrogate for the admixing source.

We applied GLOBETROTTER as described in Section S9, using paintings under the **All-donors** analysis using the *DRC-all-world* dataset. We tested every African popuation (i.e. as a target group) and used all other globel populations as surrogates, except for African Caribbeans in Barbados (ACB) and Americans of African Ancestry in SW USA (ASW) from The 1000 Genomes Project Consortium (2015), both of which have recent admixture that makes interpreting any inferred matching to them challenging. Analagous to the protocol described in Section S9, we performed two paintings when testing each target group: one painted each individual using all other individuals as surrogates, while the other painting only individuals from the target group using all non-target group individuals as surrogates. Furthermore, to evaluate the effect of including our novel DRC samples as surrogates, we also performed two separate GLOBETROTTER analyses, one that included and one that excluded the DRC as a potential surrogate.

For analyses including DRC as a surrogate, we detected evidence of admixture in 33 of the 36 African populations tested. For 15 of these 33 populations, we inferred a single admixture event occuring at one time between two sources (Table S22). The remaining populations inferred more complex admixture events, with 8 showing evidence for multiple admixture events occuring at approximately the same time (Table S23), and 10 showing evidence for multiple dates of admixture with date point estimates ranging from 3 to 182 generations ago (Table S24). Introgression from a source inferred to match >5% of their DNA to the DRC occurred in 13 of these 35 cases. Of these, ten represent Bantu-speaking groups, sampled from Western (Yoruba, Bongo Gabon South, Bongo Gabon East, Nzime, Nzebi), Eastern (Bakiga, Batwa, Luhya) and Southern (SWBantu, SEBantu) Africa, with DRC inferred dates older than 100BCE in two groups (YRI and SEBantu) but otherwise more recent. The remaining non-Bantu groups with a source matching $> 5\%$ of their DNA to the DRC were the Khwe and Xun, both Southern Khoe-san groups sampled from neighbouring Angola, the GuiGhanaKgal and the Baka of Cameroon.

Analagous results when excluding the DRC as a potential surrogate are provided for single admixture events (Table S25), multiple events at a single time (Table S26), and multiple dates of admixture (Table S27). Inferred dates remain very consistent across target groups in this analysis, with the DRC contribution replaced by a mixture of other Bantu-speaking African populations, predominantly those from Cameroon, Gabon and Nigeria, Figure S18.

Whilst these results do not date to the proposed timing of early Bantu expansion waves, they do highlight the important role of the DRC, as a surrogate for a large Bantu-like ancestry component that is wide-spread across Africa today.

Target	gens	date	%S1	best.S1	S1>10%	%S2	best.S2	S2>10%
AMHARA*	58(53-65)	298CE(102CE-438CE)	0.49	IBS	TSI(0.19)IBS(0.22)TYGRAY(0.43)	0.51	WOLAYTA	SOMALI(0.1)ANUAK(0.22)OROMO(0.27)
Baka_Gab	7(4-7)	1726CE(1726CE-1810CE)	0.12	Nzebi_Gab	LWK(0.19)Nzime_Cam(0.22)SWBantu(0.4)	0.88	Baka_Cam	Baka_Cam(1)
Bongo_GabE	27(24-30)	1166CE(1082CE-1250CE)	0.35	Bongo_GabS	Baka_Gab(0.14)Bongo_GabS(0.5)	0.65	Nzebi_Gab	YRI(0.1)Nzebi_Gab(0.21)DRC(0.46)
Bongo_GabS*	31(27-34)	1166CE(1082CE-1250CE)	0.43	Bongo_GabE	Baka_Cam(0.12)Baka_Gab(0.14)Bongo_GabE(0.41)	0.57	Nzebi_Gab	Nzebi_Gab(0.24)DRC(0.47)
ESN	82(43-122)	374BCE(1494BCE-718CE)	0.08	DRC	Nzebi_Gab(0.3)DRC(0.48)	0.92	YRI	YRI(0.98)
ESOMALI*	40(31-48)	802CE(578CE-1054CE)	0.23	ColouredWellington	AMHARA(0.14)BEB(0.19)IBS(0.21)	0.77	SOMALI	SOMALI(0.92)
GUMUZ*	13(9-23)	1558CE(1278CE-1670CE)	0.33	ANUAK	SUDANESE(0.14)ANUAK(0.41)	0.67	DRC	ANUAK(0.18)WOLAYTA(0.35)
Juhoansi*	52(44-63)	466CE(158CE-690CE)	0.19	Nama	GuiGhanaKgal(0.11)Nama(0.23)Karretjie(0.24)Khomani(0.31)	0.81	Xun	Khomani(0.15)Xun(0.74)
Khomani*	6(6-6)	1754CE(1754CE-1754CE)	0.14	GBR	CEU(0.24)GBR(0.7)	0.86	Nama	Karretjie(0.27)Nama(0.66)
Khwe*	20(19-22)	1362CE(1306CE-1390CE)	0.49	Xun	Juhoansi(0.14)Nama(0.17)Xun(0.26)	0.51	DRC	DRC(0.66)
MSL*	79(62-106)	290BCE(1046BCE-186CE)	0.36	GWD	GWD(0.75)	0.64	YRI	GWD(0.13)YRI(0.73)
Nzebi_Gab	33(29-38)	998CE(858CE-1110CE)	0.12	Bongo_GabS	Baka_Cam(0.16)Bongo_GabE(0.18)Bongo_GabS(0.26)	0.88	DRC	YRI(0.13)DRC(0.57)
SUDANESE*	16(11-21)	1474CE(1334CE-1614CE)	0.18	Nzebi_Gab	GWD(0.11)DRC(0.12)LWK(0.14)YRI(0.24)	0.82	ANUAK	ANUAK(0.89)
TYGRAY*	56(52-60)	354CE(242CE-466CE)	0.38	TSI	AMHARA(0.18)TSI(0.27)IBS(0.28)	0.62	WOLAYTA	SUDANESE(0.16)OROMO(0.52)
WOLAYTA*	44(38-50)	690CE(522CE-858CE)	0.34	ARICULTIVATOR	ARICULTIVATOR(0.52)	0.66	AMHARA	AMHARA(0.76)

Table S22: Details of admixture inference in sampled African populations where GLOBETROTTER infers a single date of admixture between two groups. Results are shown for Nullind:1. The date of admixture is provided in generations ("gens") and years, with bootstrap 95% CIs given in parenthesis, and with generations converted to years using the formula: $1950 - (g + 1) \times 28$. The proportion (%.S1, %.S2) and best matching surrogate (best.S1, best.S2) for each inferred source of each admixture event are provided, as is the more detailed inferred composition of each source (S1 and S2) as a mixture of surrogates (only surrogates contributing >10% are shown). Contributions from the DRC are colored in red. Those results marked with an asterisk (*) in the first column should be interpreted with caution due to discrepencies (i.e. differing inference in the type of event or non-overlapping date estimates) between Nullind:1 and Nullind:0 results.

**Event1**								
Target	gens	date	%S1	best.S1	S1>10%	%S2	best.S2	S2>10%
AFAR	48(43-52)	578CE(466CE-718CE)	0.2	TSI	IBS(0.31)TSI(0.36)	0.8	OROMO	SOMALI(0.2)TYGRAY(0.26)OROMO(0.4)
ANUAK	32(25-39)	1026CE(830CE-1222CE)	0.25	DRC	LWK(0.1)GUMUZ(0.11)ARICULTIVATOR(0.13)YRI(0.17)	0.75	SUDANESE	SUDANESE(0.87)
ARIBLACKSMITH*	64(46-78)	130CE(262BCE-634CE)	0.42	WOLAYTA	ANUAK(0.17)OROMO(0.66)	0.58	ARICULTIVATOR	ARICULTIVATOR(0.93)
ARICULTIVATOR*	75(66-87)	178BCE(514BCE-74CE)	0.28	DRC	GUMUZ(0.14)ANUAK(0.17)ARIBLACKSMITH(0.22)	0.72	WOLAYTA	WOLAYTA(0.88)
Batwa	29(26-32)	1110CE(1026CE-1194CE)	0.45	Nzebi_Gab	LWK(0.13)Bakiga(0.19)	0.55	Nzebi_Gab	LWK(0.22)DRC(0.29)Bakiga(0.37)
Karretjie	4(4-5)	1810CE(1782CE-1810CE)	0.18	GBR	GBR(0.94)	0.82	Nama	Nama(0.11)SEBantu(0.21)Khomani(0.58)
Nzime_Cam*	34(30-37)	970CE(886CE-1082CE)	0.18	Nzebi_Gab	YRI(0.23)Baka_Cam(0.23)	0.82	DRC	YRI(0.26)DRC(0.43)
YRI*	87(73-106)	514BCE(1046BCE-122BCE)	0.27	DRC	DRC(0.16)GWD(0.21)MSL(0.23)	0.73	ESN	MSL(0.14)ESN(0.62)
**Event2**								
Target	gens	date	%S1	best.S1	S1>10%	%S2	best.S2	S2>10%
AFAR	48(43-52)	578CE(466CE-718CE)	0.35	ESOMALI	TSI(0.12)TYGRAY(0.18)AMHARA(0.19)SOMALI(0.37)	0.65	OROMO	AMHARA(0.21)TYGRAY(0.6)
ANUAK	32(25-39)	1026CE(830CE-1222CE)	0.21	YRI	SUDANESE(0.14)LWK(0.15)YRI(0.33)	0.79	SUDANESE	SUDANESE(0.76)
ARIBLACKSMITH*	64(46-78)	130CE(262BCE-634CE)	0.31	ANUAK	ANUAK(0.33)ARICULTIVATOR(0.33)	0.69	ARICULTIVATOR	AMHARA(0.27)ARICULTIVATOR(0.61)
ARICULTIVATOR*	75(66-87)	178BCE(514BCE-74CE)	0.32	DRC	YRI(0.1)ANUAK(0.2)WOLAYTA(0.32)	0.68	WOLAYTA	ARIBLACKSMITH(0.11)WOLAYTA(0.77)
Batwa	29(26-32)	1110CE(1026CE-1194CE)	0.39	Bakiga	AMHARA(0.13)LWK(0.2)Bakiga(0.47)	0.61	Nzebi_Gab	DRC(0.18)Bakiga(0.18)LWK(0.18)
Karretjie	4(4-5)	1810CE(1782CE-1810CE)	0.34	SEBantu	ColouredColesberg(0.42)SEBantu(0.5)	0.66	Khomani	Khomani(0.86)
Nzime_Cam*	34(30-37)	970CE(886CE-1082CE)	0.47	Nzebi_Gab	DRC(0.21)YRI(0.42)	0.53	Nzebi_Gab	YRI(0.11)Nzebi_Gab(0.11)DRC(0.48)
YRI*	87(73-106)	514BCE(1046BCE-122BCE)	0.48	Nzebi_Gab	MSL(0.11)DRC(0.21)ESN(0.38)	0.52	ESN	MSL(0.22)ESN(0.51)

Table S23: Details of admixture inference in sampled African populations where GLOBETROTTER infers multiple admixture events occurring at around the same time. Results are shown for NullInd:1, and for each admixture event. The date of admixture is provided in generations ("gens") and years, with bootstrap 95% CIs given in parenthesis, and with generations converted to years using the formula: $1950 - (g + 1) \times 28$. The proportion (%.S1, %.S2) and best matching surrogate (best.S1, best.S2) for each inferred source of each admixture event are provided, as is the more detailed inferred composition of each source (S1 and S2) as a mixture of surrogates (only surrogates contributing >10% are shown). Contributions from the DRC are colored in red. Those results marked with an asterisk (*) in the first column should be interpreted with caution due to discrepencies (i.e. differing inference in the type of event or non-overlapping date estimates) between Nullind:1 and Nullind:0 results.

**Date1**								
Target	gens	date	%S1	best.S1	S1>10%	%S2	best.S2	S2>10%
Baka_Cam*	11(7-13)	1614CE(1558CE-1726CE)	0.25	Nzebi_Gab	ESN(0.15)DRC(0.2)Baka_Gab(0.39)	0.75	Baka_Gab	Baka_Gab(0.9)
ColouredColesberg	6(2-8)	1754CE(1698CE-1866CE)	0.27	CEU	GBR(0.23)CEU(0.5)	0.73	SEBantu	SEBantu(0.34)Karretjie(0.52)
ColouredWellington	4(2-5)	1810CE(1782CE-1866CE)	0.45	SEBantu	Nama(0.1)Karretjie(0.14)SEBantu(0.46)	0.55	CEU	BEB(0.19)CEU(0.38)
GuiGhanaKgal	5(2-7)	1782CE(1726CE-1866CE)	0.36	SEBantu	Khomani(0.13)DRC(0.27)SEBantu(0.53)	0.64	Nama	Xun(0.11)Juhoansi(0.15)Karretjie(0.16)Khomani(0.39)
GWD	9(4-12)	1670CE(1586CE-1810CE)	0.25	YRI	YRI(0.26)MSL(0.31)	0.75	YRI	YRI(0.33)MSL(0.35)
LWK	15(12-17)	1502CE(1446CE-1586CE)	0.26	ANUAK	SUDANESE(0.15)AMHARA(0.16)ANUAK(0.46)	0.74	DRC	Bakiga(0.19)DRC(0.42)
OROMO	6(2-9)	1754CE(1670CE-1866CE)	0.32	WOLAYTA	ARICULTIVATOR(0.16)AMHARA(0.47)	0.68	AMHARA	TYGRAY(0.13)AMHARA(0.77)
SEBantu	22(12-25)	1306CE(1222CE-1586CE)	0.28	Karretjie	Khomani(0.23)Karretjie(0.59)	0.72	DRC	LWK(0.21)DRC(0.52)
SWBantu*	3(1-3)	1838CE(1838CE-1894CE)	0.2	Nama	Nama(0.6)	0.8	DRC	Nama(0.2)DRC(0.54)
Xun*	4(1-8)	1810CE(1698CE-1894CE)	0.24	DRC	Juhoansi(0.12)Khomani(0.15)Khwe(0.21)DRC(0.36)	0.76	Juhoansi	Nama(0.13)Khwe(0.16)Juhoansi(0.52)
**Date2**								
Target	gens	date	%S1	best.S1	S1>10%	%S2	best.S2	S2>10%
Baka_Cam*	56(33-72)	346CE(94BCE-998CE)	0.36	DRC	ESN(0.11)DRC(0.37)Baka_Gab(0.52)	0.64	Baka_Gab	Bongo_GabS(0.27)Baka_Gab(0.73)
ColouredColesberg	17(9-42)	1435CE(746CE-1670CE)	0.42	SEBantu	GBR(0.12)CEU(0.14)SEBantu(0.51)	0.58	Karretjie	Karretjie(0.63)
ColouredWellington	23(9-41)	1288CE(774CE-1670CE)	0.48	SEBantu	Karretjie(0.13)CEU(0.17)SEBantu(0.41)	0.52	BEB	CEU(0.11)BEB(0.14)
GuiGhanaKgal	38(32-40)	854CE(802CE-1026CE)	0.25	DRC	LWK(0.13)DRC(0.51)	0.75	Nama	Juhoansi(0.11)SEBantu(0.13)Karretjie(0.23)Khomani(0.38)
GWD	90(62-114)	605BCE(1270BCE-186CE)	0.11	DRC	IBS(0.2)YRI(0.29)	0.89	YRI	YRI(0.32)MSL(0.38)
LWK	55(46-65)	376CE(102CE-634CE)	0.31	DRC	Bakiga(0.14)SUDANESE(0.23)	0.69	DRC	Bakiga(0.15)DRC(0.54)
OROMO	68(59-79)	25CE(290BCE-270CE)	0.34	WOLAYTA	SOMALI(0.12)ANUAK(0.2)ARICULTIVATOR(0.26)	0.66	TYGRAY	AMHARA(0.63)
SEBantu	130(53-182)	1720BCE(3174BCE-438CE)	0.43	Karretjie	Khomani(0.15)GuiGhanaKgal(0.17)Karretjie(0.49)	0.57	DRC	LWK(0.17)DRC(0.83)
SWBantu*	65(34-80)	112CE(318BCE-970CE)	0.36	Nama	DRC(0.14)Nama(0.78)	0.64	DRC	DRC(0.6)
Xun*	32(28-36)	1032CE(914CE-1138CE)	0.19	DRC	Nzebi_Gab(0.11)DRC(0.54)	0.81	Juhoansi	Khomani(0.11)Khwe(0.17)Juhoansi(0.53)

Table S24: Details of admixture inference in sampled African populations where GLOBETROTTER infers multiple dates of admixture. Results are shown for NullInd:1, and for each admixture event. The date of admixture is provided in generations ("gens") and years, with bootstrap 95% CIs given in parenthesis, and with generations converted to years using the formula: $1950-(g+1)\times 28$. The proportion (%.S1, %.S2) and best matching surrogate (best.S1, best.S2) for each inferred source of each admixture event are provided, as is the more detailed inferred composition of each source (S1 and S2) as a mixture of surrogates (only surrogates contributing >10% are shown). Contributions from the DRC are colored in red. Those results marked with an asterisk (*) in the first column should be interpreted with caution due to discrepencies (i.e. differing inference in the type of event or non-overlapping date estimates) between Nullind:1 and Nullind:0 results.

Target	gens	date	%S1	best.S1	S1>10%	%S2	best.S2	S2>10%
AMHARA*	58(51-63)	298CE(158CE-494CE)	0.49	IBS	TSI(0.19)IBS(0.22)TYGRAY(0.43)	0.51	WOLAYTA	SOMALI(0.1)ANUAK(0.22)OROMO(0.27)
Baka_Gab	7(4-7)	1726CE(1726CE-1810CE)	0.13	LWK	SWBantu(0.13)Nzime_Cam(0.36)LWK(0.49)	0.87	Baka_Cam	Baka_Cam(1)
Bongo_GabE	24(22-26)	1250CE(1194CE-1306CE)	0.23	Bongo_GabS	Baka_Gab(0.11)Baka_Cam(0.15)Bongo_GabS(0.58)	0.77	Nzebi_Gab	Nzebi_Gab(0.9)
Bongo_GabS	26(22-30)	1194CE(1082CE-1306CE)	0.31	Bongo_GabE	Baka_Cam(0.19)Bongo_GabE(0.46)	0.69	Nzebi_Gab	Nzebi_Gab(0.91)
ESN	76(57-104)	206BCE(990BCE-326CE)	0.27	YRI	Nzebi_Gab(0.44)YRI(0.56)	0.73	YRI	YRI(0.99)
ESOMALI*	40(32-46)	802CE(634CE-1026CE)	0.23	ColouredWellington	AMHARA(0.16)BEB(0.18)IBS(0.2)	0.77	SOMALI	SOMALI(0.92)
GUMUZ*	16(8-32)	1474CE(1026CE-1698CE)	0.48	Nzebi_Gab	ANUAK(0.14)WOLAYTA(0.41)	0.52	ANUAK	WOLAYTA(0.11)SUDANESE(0.13)ANUAK(0.36)
Juhoansi*	52(43-63)	466CE(158CE-718CE)	0.19	Nama	GuiGhanaKgal(0.11)Nama(0.23)Karretjie(0.24)Khomani(0.31)	0.81	Xun	Khomani(0.15)Xun(0.74)
Khomani*	6(5-6)	1754CE(1754CE-1782CE)	0.14	GBR	CEU(0.24)GBR(0.7)	0.86	Nama	Karretjie(0.27)Nama(0.66)
Khwe*	18(17-20)	1418CE(1362CE-1446CE)	0.46	Xun	GuiGhanaKgal(0.12)Juhoansi(0.15)Nama(0.19)SEBantu(0.22)Xun(0.26)	0.54	Nzebi_Gab	Nzebi_Gab(0.99)
MSL*	75(56-97)	178BCE(794BCE-354CE)	0.33	GWD	Nzebi_Gab(0.1)GWD(0.75)	0.67	YRI	GWD(0.16)YRI(0.69)
SUDANESE	16(10-24)	1474CE(1250CE-1642CE)	0.17	YRI	GWD(0.11)Nzebi_Gab(0.12)LWK(0.16)YRI(0.3)	0.83	ANUAK	ANUAK(0.88)
SWBantu*	16(13-19)	1474CE(1390CE-1558CE)	0.23	ColouredColesberg	Nzebi_Gab(0.14)SEBantu(0.28)Nama(0.58)	0.77	Nzebi_Gab	Nzebi_Gab(0.91)
TYGRAY	56(51-60)	354CE(242CE-494CE)	0.38	TSI	AMHARA(0.18)TSI(0.27)IBS(0.28)	0.62	WOLAYTA	SUDANESE(0.16)OROMO(0.52)
WOLAYTA	44(37-51)	690CE(494CE-886CE)	0.34	ARICULTIVATOR	ARICULTIVATOR(0.52)	0.66	AMHARA	AMHARA(0.76)

Table S25: Details of admixture inference in sampled African populations where GLOBETROTTER infers a single date of admixture between two groups, when *excluding* the DRC as a potential surrogate. Results are shown for NullInd:1. The date of admixture is provided in generations ("gens") and years, with bootstrap 95% CIs given in parenthesis, and with generations converted to years using the formula: $1950 - (g + 1) \times 28$. The proportion (%.S1, %.S2) and best matching surrogate (best.S1, best.S2) for each inferred source of each admixture event are provided, as is the more detailed inferred composition of each source (S1 and S2) as a mixture of surrogates (only surrogates contributing >10% are shown). Those results marked with an asterisk (*) in the first column should be interpreted with caution due to discrepencies (i.e. differing inference in the type of event or non-overlapping date estimates) between Nullind:1 and Nullind:0 results.

Event1								
Target	gens	date	%S1	best.S1	S1>10%	%S2	best.S2	S2>10%
AFAR	48(42-53)	578CE(438CE-746CE)	0.2	TSI	IBS(0.31)TSI(0.36)	0.8	OROMO	SOMALI(0.2)TYGRAY(0.26)OROMO(0.4)
ANUAK	31(25-39)	1054CE(830CE-1222CE)	0.26	Nzebi_Gab	GUMUZ(0.1)ARICULTIVATOR(0.13)OROMO(0.13)YRI(0.16)	0.74	SUDANESE	SUDANESE(0.87)
ARIBLACKSMITH*	64(48-76)	130CE(206BCE-578CE)	0.42	WOLAYTA	ANUAK(0.17)OROMO(0.66)	0.58	ARICULTIVATOR	ARICULTIVATOR(0.93)
ARICULTIVATOR*	74(61-88)	150BCE(542BCE-214CE)	0.28	Nzebi_Gab	GUMUZ(0.14)ANUAK(0.17)ARIBLACKSMITH(0.22)	0.72	WOLAYTA	WOLAYTA(0.87)
Baka_Cam	12(11-13)	1586CE(1558CE-1614CE)	0.27	Baka_Gab	YRI(0.17)Baka_Gab(0.42)	0.73	Baka_Gab	Baka_Gab(0.89)
Batwa	28(26-32)	1138CE(1026CE-1194CE)	0.27	Nzebi_Gab	ANUAK(0.17)Bakiga(0.18)	0.73	Nzebi_Gab	LWK(0.21)Bakiga(0.33)Nzebi_Gab(0.35)
Karretjie	4(4-5)	1810CE(1782CE-1810CE)	0.18	GBR	GBR(0.94)	0.82	Nama	Nama(0.1)SEBantu(0.22)Khomani(0.58)
Nzebi_Gab	25(18-35)	1222CE(942CE-1418CE)	0.12	SEBantu	Bongo_GabE(0.22)SEBantu(0.36)Nzime_Cam(0.37)	0.88	SEBantu	Nzime_Cam(0.18)SEBantu(0.61)
Nzime_Cam	23(21-25)	1278CE(1222CE-1334CE)	0.15	Nzebi_Gab	Baka_Cam(0.21)Nzebi_Gab(0.76)	0.85	Nzebi_Gab	YRI(0.26)Nzebi_Gab(0.67)
YRI*	68(33-92)	18CE(654BCE-998CE)	0.23	Nzebi_Gab	GWD(0.21)MSL(0.25)Nzebi_Gab(0.28)	0.77	ESN	MSL(0.13)Nzebi_Gab(0.25)ESN(0.57)
**Event2**								
Target	gens	date	%S1	best.S1	S1>10%	%S2	best.S2	S2>10%
AFAR	48(42-53)	578CE(438CE-746CE)	0.35	ESOMALI	TSI(0.12)TYGRAY(0.18)AMHARA(0.19)SOMALI(0.37)	0.65	OROMO	AMHARA(0.21)TYGRAY(0.6)
ANUAK	31(25-39)	1054CE(830CE-1222CE)	0.23	YRI	LWK(0.15)SUDANESE(0.17)YRI(0.32)	0.77	SUDANESE	SUDANESE(0.76)
ARIBLACKSMITH*	64(48-76)	130CE(206BCE-578CE)	0.31	ANUAK	ANUAK(0.33)ARICULTIVATOR(0.33)	0.69	ARICULTIVATOR	AMHARA(0.27)ARICULTIVATOR(0.61)
ARICULTIVATOR*	74(61-88)	150BCE(542BCE-214CE)	0.32	Nzebi_Gab	ANUAK(0.2)WOLAYTA(0.32)	0.68	WOLAYTA	ARIBLACKSMITH(0.11)WOLAYTA(0.77)
Baka_Cam	12(11-13)	1586CE(1558CE-1614CE)	0.4	Baka_Gab	Bongo_GabS(0.12)Baka_Gab(0.78)	0.6	Baka_Gab	ESN(0.13)Baka_Gab(0.77)
Batwa	28(26-32)	1138CE(1026CE-1194CE)	0.49	Nzebi_Gab	Bakiga(0.13)LWK(0.15)Nzebi_Gab(0.3)	0.51	Bakiga	Nzebi_Gab(0.14)LWK(0.21)Bakiga(0.44)
Karretjie	4(4-5)	1810CE(1782CE-1810CE)	0.34	SEBantu	ColouredColesberg(0.43)SEBantu(0.49)	0.66	Khomani	Khomani(0.85)
Nzebi_Gab	25(18-35)	1222CE(942CE-1418CE)	0.46	SEBantu	Nzime_Cam(0.19)SEBantu(0.58)	0.54	SEBantu	YRI(0.19)Nzime_Cam(0.2)SEBantu(0.56)
Nzime_Cam	23(21-25)	1278CE(1222CE-1334CE)	0.14	YRI	YRI(0.76)	0.86	Nzebi_Gab	YRI(0.13)Nzebi_Gab(0.8)
YRI*	68(33-92)	18CE(654BCE-998CE)	0.22	ESN	GWD(0.15)MSL(0.25)ESN(0.48)	0.78	Nzebi_Gab	MSL(0.13)Nzebi_Gab(0.31)ESN(0.43)

Table S26: Details of admixture inference in sampled African populations where GLOBETROTTER infers multiple admixture events occurring at around the same time, when *excluding* the DRC as a potential surrogate. Results are shown for Nullind:1, and for each admixture event. The date of admixture is provided in generations ("gens") and years, with bootstrap 95% CIs given in parenthesis, and with generations converted to years using the formula: $1950 - (g + 1) \times 28$. The proportion (%.S1, %.S2) and best matching surrogate (best.S1, best.S2) for each inferred source of each admixture event are provided, as is the more detailed inferred composition of each source (S1 and S2) as a mixture of surrogates (only surrogates contributing >10% are shown). Those results marked with an asterisk (*) in the first column should be interpreted with caution due to discrepencies (i.e. differing inference in the type of event or non-overlapping date estimates) between Nullind:1 and Nullind:0 results.

Date1								
Target	gens	date	%S1	best.S1	S1>10%	%S2	best.S2	S2>10%
ColouredColesberg	6(4-8)	1754CE(1698CE-1810CE)	0.24	CEU	GBR(0.14)CEU(0.65)	0.76	SEBantu	SEBantu(0.37)Karretjie(0.47)
ColouredWellington	4(2-5)	1810CE(1782CE-1866CE)	0.45	SEBantu	Nama(0.1)Karretjie(0.13)SEBantu(0.5)	0.55	BEB	BEB(0.2)CEU(0.37)
GWD	9(4-15)	1670CE(1502CE-1810CE)	0.22	YRI	Nzebi_Gab(0.14)YRI(0.24)MSL(0.31)	0.78	YRI	Nzebi_Gab(0.13)YRI(0.32)MSL(0.35)
LWK*	15(12-17)	1502CE(1446CE-1586CE)	0.15	ANUAK	SUDANESE(0.24)ANUAK(0.44)	0.85	Nzebi_Gab	SEBantu(0.12)Bakiga(0.16)Nzebi_Gab(0.72)
Nama	4(1-4)	1810CE(1810CE-1894CE)	0.23	SWBantu	Karretjie(0.11)Khomani(0.18)SWBantu(0.61)	0.77	Khomani	Khomani(0.85)
SEBantu	21(16-25)	1334CE(1222CE-1474CE)	0.2	Karretjie	Juhoansi(0.1)GuiGhanaKgal(0.14)Khomani(0.31)Karretjie(0.42)	0.8	Nzebi_Gab	LWK(0.11)Nzebi_Gab(0.87)
Xun	3(1-6)	1838CE(1754CE-1894CE)	0.26	Nzebi_Gab	Juhoansi(0.13)Khwe(0.24)Nzebi_Gab(0.47)	0.74	Juhoansi	Nama(0.11)Khwe(0.15)Juhoansi(0.52)
**Date2**								
Target	gens	date	%S1	best.S1	S1>10%	%S2	best.S2	S2>10%
ColouredColesberg	15(9-40)	1503CE(802CE-1670CE)	0.39	SEBantu	Nzebi_Gab(0.13)GBR(0.14)SEBantu(0.61)	0.61	Karretjie	Khomani(0.15)Karretjie(0.52)
ColouredWellington	22(10-38)	1312CE(858CE-1642CE)	0.49	SEBantu	CEU(0.17)SEBantu(0.54)	0.51	BEB	CEU(0.11)BEB(0.15)
GWD	97(69-134)	789BCE(1830BCE-10BCE)	0.09	Nzebi_Gab	IBS(0.26)YRI(0.26)	0.91	YRI	Nzebi_Gab(0.13)YRI(0.31)MSL(0.37)
LWK*	55(46-64)	380CE(130CE-634CE)	0.22	ANUAK	AFAR(0.12)Bakiga(0.15)ANUAK(0.17)SUDANESE(0.17)	0.78	Nzebi_Gab	Bakiga(0.1)Nzebi_Gab(0.86)
Nama	42(17-57)	732CE(326CE-1446CE)	0.33	ColouredColesberg	SWBantu(0.31)Khomani(0.5)	0.67	Khomani	Karretjie(0.13)Khomani(0.79)
SEBantu	101(68-161)	910BCE(2586BCE-18CE)	0.26	Karretjie	Juhoansi(0.13)Khomani(0.18)GuiGhanaKgal(0.23)Karretjie(0.46)	0.74	Nzebi_Gab	Nzebi_Gab(0.96)
Xun	29(26-32)	1102CE(1026CE-1194CE)	0.2	Nzebi_Gab	Khwe(0.14)Nzebi_Gab(0.86)	0.8	Juhoansi	Nama(0.11)Khwe(0.17)Juhoansi(0.53)

Table S27: Details of admixture inference in sampled African populations where GLOBETROTTER infers multiple dates of admixture, when *excluding* the DRC as a potential surrogate. Results are shown for Nullind:1, and for each admixture event. The date of admixture is provided in generations ("gens") and years, with bootstrap 95% CIs given in parenthesis, and with generations converted to years using the formula: $1950 - (g + 1) \times 28$. The proportion (%.S1, %.S2) and best matching surrogate (best.S1, best.S2) for each inferred source of each admixture event are provided, as is the more detailed inferred composition of each source (S1 and S2) as a mixture of surrogates (only surrogates contributing >10% are shown). Those results marked with an asterisk (*) in the first column should be interpreted with caution due to discrepencies (i.e. differing inference in the type of event or non-overlapping date estimates) between Nullind:1 and Nullind:0 results.

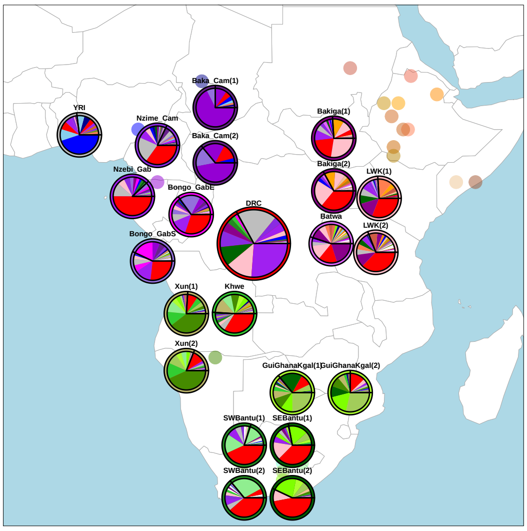

(a)

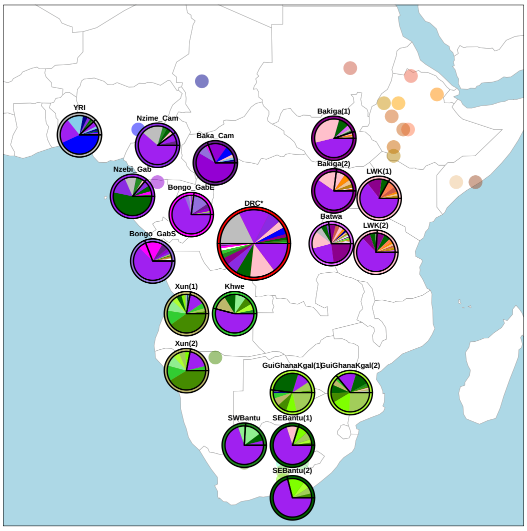

(b)

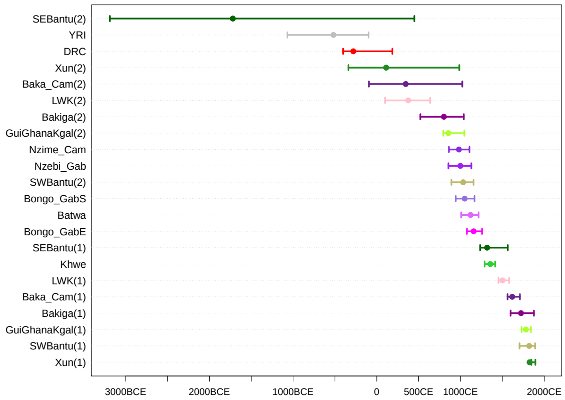

(c)

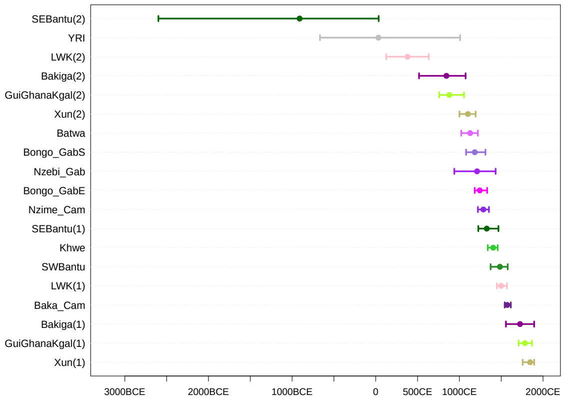

(d)

Figure S18: (a) GLOBETROTTER inferred admixing source contributions (using null.ind:1) in populations where the DRC were inferred to contribute ≥5%; other target populations are shown as translucent circles. (b) GLOBETROTTER results (using null.ind:1) for the same groups when *excluding* the DRC as a potential surrogate. Contributions are colored as in Figure S13. In both (a) and (b) contributions from non-African populations are given in white. For each inferred admixture event, the genetic make-up of each admixing source is shown, separated by dark black lines. Both events are shown for groups with multiple inferred events, with "(1)" denoting the more recent, or most strongly signalled, event. The inferred admixture into the DRC is provided in the enlarged pie as provided in Table S21 and Figure S16. (c-d) Inferred GLOBETROTTER dates (and 95% CIs) of all admixture events inferred for populations depicted in (a-b). Full details are provided in Tables S22-S27.

References

Acemoglu, D. and Robinson, J. *Why nations fail: The origins of power, prosperity, and poverty.* Crown Business, 2012.

Browning, B.L. and Browning, S.R. A fast, powerful method for detecting identity by descent. *The American Journal of Human Genetics*, 88(2):173–182, 2011.

Busby, G.B., Band, G., Si Le, Q., Jallow, M., Bougama, E., Mangano, V.D., Amenga-Etego, L.N., Enimil, A., Apinjoh, T., Ndila, C.M. et al. Admixture into and within sub-Saharan Africa. *eLife*, 5:e15266, 2016.

Bustin, E. *Lunda under Belgian rule: The politics of ethnicity.* Havard University Press Cambridge, 1975.

Cavalli-Sforza, L., Menozzi, P. and Piazza, A. *The History and Geography of Human Genes.* Princeton University Press, 1994.

Chacon-Duque, J.C., Adhikari, K., Fuentes-Guajardo, M., Mendoza-Revilla, J., Acuna-Alonzo, V., Lozano, R.B., Quinto-Sanchez, M., Gomez-Valdes, J., Martinez, P.E., Villamil-Ramirez, H. et al. Latin americans show wide-spread converso ancestry and the imprint of local native ancestry on physical appearance. *bioRxiv*, page 252155, 2018.

Chang, C.C., Chow, C.C., Tellier, L.C., Vattikuti, S., Purcell, S.M. and Lee, J.J. Second-generation plink: rising to the challenge of larger and richer datasets. *GigaScience*, 4(1):7, 2015.

Cockerham, C. and Weir, B. Estimation of inbreeding parameters in stratified populations. *Annals of Human Genetics*, 50:271–281, 1986.

de Filippo, C., Bostoen, K., Stoneking, M. and Pakendorf, B. Bringing together linguistic and genetic evidence to test the Bantu expansion. *Proceedings of the Royal Society of London B: Biological Sciences*, 279(1741):3256–3263, 2012.

Dedeken, N.C. *Chimères Baluba: Le Sud-Kasaï, 1960-1962 à feu et à sang.* Dedekun Editeur, Brussels, 1978.

Douglas, M. *The Lele of the Kasai*, volume 1. Routledge, 1963.

Douglas, M. Lele economy compared with the Bushong. *Perspectives on Africa: A Reader in Culture, History, and Representation*, 2:101, 1997.

Eggert, M.K. The central african rain forest: historical speculation and archaeological facts. *World Archaeology*, 24(1):1–24, 1992.

Ehret, C. Establishment of iron-working in eastern, central, and southern africa: Linguistic inferences on technological history. *Sprache und Geschichte in Afrika*, 16:125–175, 2001.

Excoffier, L., Laval, G. and Schneider, S. Arlequin (version 3.0): an integrated software package for population genetics data analysis. *Evolutionary Bioinformatics Online*, 1:47, 2005.

Guthrie, M. *Comparative Bantu: an introduction to the comparative linguistics and prehistory of the Bantu languages.*, volume 1 of *The comparative linguistics of the Bantu languages.* Gregg Press, Farnborough, 1971.

Hellenthal, G., Busby, G., Band, G., Wilson, J., Capelli, C., Falush, D. and Myers, S. A genetic atlas of human admixture history. *Science*, 343:747–751, 2014.

Jostins, L., Xu, Y., McCarthy, S., Ayub, Q., Durbin, R., Barrett, J. and Tyler-Smith, C. YFitter: Maximum likelihood assignment of Y chromosome haplogroups from low-coverage sequence data. *arXiv preprint arXiv:1407.7988*, 2014.

Karafet, T.M., Mendez, F.L., Meilerman, M.B., Underhill, P.A., Zegura, S.L. and Hammer, M.F. New binary polymorphisms reshape and increase resolution of the human y chromosomal haplogroup tree. *Genome Research*, 18(5):830–838, 2008.

Klieman, K.A. *"The pygmies were our compass": Bantu and Batwa in the history of west central Africa, early times to c. 1900 CE.* Heinemann London, 2003.

Lawson, D., Hellenthal, G., Myers, S. and Falush, D. Inference of population structure using dense haplotype data. *PLoS Genetics*, 8(1):e1002453, 2012.

Leslie, S., Winney, B., Hellenthal, G., Davison, D., Boumertit, A., Day, T., Hutnik, K., Royrvik, E., Cunliffe, B., Wellcome Trust Case Control Consortium 2 et al. The fine scale genetic structure of the British population. *Nature*, 519:309–314, 2015.

Li, S., Schlebusch, C. and Jakobsson, M. Genetic variation reveals large-scale population expansion and migration during the expansion of Bantu-speaking peoples. In *Proc. R. Soc. B*, volume 281, page 20141448. The Royal Society, 2014.

Lowes, S., Nunn, N., Robinson, J.A. and Weigel, J. The evolution of culture and institutions: Evidence from the Kuba Kingdom. *Econometrica Journal of the Econometric Society*, 85(4): 1065–1091, 2017.

Martens, D.S. *A history of European penetration and African reaction in the Kasai region of Zaire, 1880-1908.* PhD thesis, Simon Fraser University. Theses (Dept. of History), 1980.

McCulloch, M. *The Southern Lunda and Related Peoples:(Northern Rhodesia, Belgian Congo, Angola).*, volume 1. International African Institute, 1951.

Nei, M. *Molecular Evolutionary Genetics.* Columbia University Press, 1987.

Newbury, D. Contradictions at the heart of the canon: Jan vansina and the debate over oral historiography in africa, 1960–1985. *History in Africa*, 34:213–254, 2007.

Pagani, L., Kivisild, T., Tarekegn, A., Ekong, R., Plaster, C., Gallego-Romero, I., Ayub, Q., Mehdi, S., Thomas, M., Luiselli, D. et al. Ethiopian genetic diversity reveals linguistic stratification and complex influences on the Ethiopian gene pool. *The American Journal of Human Genetics*, 91(1):83–96, 2012.

Patin, E., Siddle, K.J., Laval, G., Quach, H., Harmant, C., Becker, N., Froment, A., Régnault, B., Lemée, L., Gravel, S. et al. The impact of agricultural emergence on the genetic history of African rainforest hunter-gatherers and agriculturalists. *Nature Communications*, 5, 2014.

Patin, E., Lopez, M., Grollemund, R., Verdu, P., Harmant, C., Quach, H., Laval, G., Perry, G.H., Barreiro, L.B., Froment, A. et al. Dispersals and genetic adaptation of Bantu-speaking populations in africa and north america. *Science*, 356(6337):543–546, 2017.

Peyrot, B. and Oslisly, R. Recherches récentes sur le paléoenvironnement et l'archéologie au gabon: 1982-1985. *l'Anthropologie*, 90(2):201–215, 1986.

Pour, N.A., Plaster, C.A. and Bradman, N. Evidence from Y-chromosome analysis for a late exclusively Eastern expansion of the Bantu-speaking people. *European Journal of Human Genetics*, 21(4):423–429, 2013.

Price, A.L., Weale, M.E., Patterson, N., Myers, S.R., Need, A.C., Shianna, K.V., Ge, D., Rotter, J.I., Torres, E., Taylor, K.D. et al. Long-range LD can confound genome scans in admixed populations. *The American Journal of Human Genetics*, 83(1):132–135, 2008.

Pruitt, W.F. *An Independent People: A History of the Sala Mpasu of Zaire and their Neighbors.* PhD thesis, Department of History Northwestern University, 1973.

Reefe, T.Q. *The Rainbow and the Kings: a history of the Luba empire to 1891.* University of California Press, 1981.

Reynolds, J., Weir, B.S. and Cockerham, C.C. Estimation of the coancestry coefficient: basis for a short-term genetic distance. *Genetics*, 105(3):767–779, 1983.

Schlebusch, C.M., Skoglund, P., Sjödin, P., Gattepaille, L.M., Hernandez, D., Jay, F., Li, S., De Jongh, M., Singleton, A., Blum, M.G. et al. Genomic variation in seven Khoe-San groups reveals adaptation and complex African history. *Science*, 338(6105):374–379, 2012.

Schütze, H., Manning, C.D. and Raghavan, P. *Introduction to Information Retrieval*, volume 39. Cambridge University Press, 2008.

Sheppard, W.H. *Presbyterian Pioneers in Congo.* Presbyterian Committee of Publication, Richmond VA, 1917.

The 1000 Genomes Project Consortium. A global reference for human genetic variation. *Nature*, 526(7571):68–74, 2015.

Tishkoff, S., Reed, F., Friedlaender, F., Ehret, C., Ranciaro, A., Froment, A., Hirbo, J., Awomoyi, A., Bodo, J., Doumbo, O. et al. The Genetic Structure and History of Africans and African Americans. *Science*, 324(5930):1035–1044, 2009.

Torday, E. *On the Trail of the Bushongo.* Seeley, Service & Company limited, 1925.

Torday, E. and Joyce, T. *Notes ethnographiques sur les peuples communément appelés Bakuba, ainsi que sur les peuplades apparentées, Les Bushongo.* Les Bushongo, Bruxelles: Ministère des Colonies, 1910.

van Dorp, L., Balding, D., Myers, S., Pagani, L., Tyler-Smith, C., Bekele, E., Tarekegn, A., Thomas, M., Bradman, N. and Hellenthal, G. Evidence for a common origin of blacksmiths and cultivators in the Ethiopian Ari within the last 4500 Years: Lessons for clustering-based inference. *PLoS Genetics*, 11(8):e1005397, 2015.

van Oven, M. PhyloTree Build 17: Growing the human mitochondrial DNA tree. *Forensic Science International: Genetics Supplement Series*, 5:e392–e394, 2015.

Vansina, J. *Introduction à l'ethnographie du Congo.* Number 1. Université Lovanium, 1966a.

Vansina, J. *Kingdoms of the Savanna.* University of Wisconsin Press, 1966b.

Vansina, J. *Man in Adaption: The Institutional Framework*, chapter A traditional legal system: the Kuba, pages 135–148. Aldine-Atherton, Chicago, 1971.

Vansina, J. *The children of Woot: a history of the Kuba peoples*. University of Wisconsin Press, 1978.

Vansina, J. Linguistic evidence for the introduction of ironworking into Bantu-speaking Africa. *History in Africa*, 33:321–361, 2006.

Vansina, J. *Being colonized: the Kuba experience in rural Congo, 1880–1960*. University of Wisconsin Press, 2010.

Verner, S.P. *Pioneering in central Africa*. Presbyterian Committee of Publication, 1903.

Weissensteiner, H., Pacher, D., Kloss-Brandstätter, A., Forer, L., Specht, G., Bandelt, H.J., Kronenberg, F., Salas, A. and Schönherr, S. HaploGrep 2: mitochondrial haplogroup classification in the era of high-throughput sequencing. *Nucleic Acids Research*, 44(W1):58–63, 2016.

Wharton, C.T. *The Leopard Hunts Alone*. Fleming H. Revell Company, 1927.

Yoder, J.C. *The Kanyok of Zaire: An institutional and ideological history to 1895*. Number 74. Cambridge University Press, 1992.
